# IKKβ is required for the formation of the NLRP3 inflammasome

**DOI:** 10.15252/embr.202050743

**Published:** 2021-08-17

**Authors:** Sambit K Nanda, Alan R Prescott, Clara Figueras‐Vadillo, Philip Cohen

**Affiliations:** ^1^ MRC Protein Phosphorylation and Ubiquitylation Unit University of Dundee Dundee UK; ^2^ Dundee Imaging Facility and Division of Cell Signalling and Immunology School of Life Sciences University of Dundee Dundee UK; ^3^ Present address: AstraZeneca Gaithersburg MD USA

**Keywords:** inflammasome, innate immunity, kinases, toll‐like receptors, trans‐golgi network, Immunology, Membranes & Trafficking, Signal Transduction

## Abstract

The rapid formation and activation of the NLRP3 inflammasome is induced by co‐stimulation with LPS and nigericin. It requires the LPS‐stimulated activation of IKKβ, which exerts its effects independently of *de novo* gene transcription, protein translation and other protein kinases activated by IKKβ. IKKβ is not required for the nigericin‐induced dispersion of the trans‐Golgi network (TGN), but to bring NLRP3 in proximity with TGN38. The nigericin‐induced dispersion of the Golgi is enhanced by co‐stimulation with LPS, and this enhancement is IKKβ‐dependent. Prolonged stimulation with LPS to increase the expression of NLRP3, followed by stimulation with nigericin, produced larger TGN38‐positive puncta, and the ensuing activation of the NLRP3 inflammasome was also suppressed by IKKβ inhibitors added prior to stimulation with nigericin. IKKβ therefore has a key role in recruiting NLRP3 to the dispersed TGN, leading to the formation and activation of the NLRP3 inflammasome.

## Graphical abstract

Formation of the NLRP3 inflammasome requires LPS to activate IKKβ and nigericin to disperse the trans‐Golgi network. IKKβ functions to recruit NLRP3 to the dispersed TGN promoting inflammasome formation and activation.

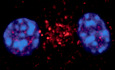

## Introduction

Signals that activate Toll‐like receptors (TLRs), such as components of microbial pathogens, induce the formation of multi‐protein complexes termed myddosomes (Motshwene *et al*, 2009; Lin *et al*, 2010), triggering the formation of ubiquitin oligomers that activate the “master” protein kinases of the innate immune system such as the transforming growth factor (TGF) β‐activated kinase 1 (TAK1, also called mitogen‐activated protein kinase kinase kinase 7 (MAP3K7)) and the canonical IκB kinase (IKK) complex (reviewed (Cohen & Strickson, 2017; Cohen *et al*, 2020)). The IKKβ component of the canonical IKK complex has several roles in this system. It switches on critical transcription factors, such as nuclear factor kappa‐light‐chain‐enhancer of activated B cells (NF‐κB) (Yaron *et al*, 1998; Spencer *et al*, 1999) and interferon regulatory factor 5 (IRF5) (Lopez‐Pelaez *et al*, 2014; Ren *et al*, 2014), and activates other protein kinases, including the IKK‐related kinases TBK1 (TANK‐binding kinase 1) and IKKɛ (Clark *et al*, 2011) and Tpl2 (also called MAP3K8) (Waterfield *et al*, 2004), and phosphorylates leucine‐rich repeat kinase 2 (LRRK2) (Dzamko *et al*, 2012). The role of TAK1 in this signalling network is to activate mitogen‐activated protein kinase kinases and to initiate the activation of IKKβ (Zhang *et al*, 2014). Together, the activation of TAK1 and IKKβ triggers the phosphorylation of a myriad of proteins that control the production, processing and secretion of the inflammatory mediators that combat microbial pathogens (Akira *et al*, 2006).

A TLR‐activating signal is also required to assemble another multi‐protein complex, the NLR family pyrin domain containing 3 (NLRP3) inflammasome, which comprises the proteins NLRP3, Apoptosis‐associated Speck‐like protein containing a CARD (ASC) protein and caspase‐1 (Lamkanfi & Dixit, 2014). However, activation of the NLRP3 inflammasome additionally requires a second signal, which can be a variety of molecules, such as extracellular ATP released when cells rupture, the antibiotic nigericin (a potassium ionophore derived from *Streptomyces hygroscopicus*), or urate or cholesterol crystals (Lamkanfi & Dixit, 2012; Rathinam *et al*, 2012). In contrast, other inflammasomes, such as the absent in melanoma 2 (AIM2) inflammasome, sense other viral and bacterial products, such as double‐stranded DNA (dsDNA) derived from these microbes (Fernandes‐Alnemri *et al*, 2009; Hornung *et al*, 2009; Fernandes‐Alnemri *et al*, 2010; Rathinam *et al*, 2010).

Inflammasomes comprise a sensor/receptor protein and a caspase and frequently one or more additional adaptor proteins. Both NLRP3 and AIM2 are sensors containing a Pyrin Domain (PYD), which undergo oligomerization to form a PYD platform that interacts with the PYD‐containing adaptor protein ASC, leading to the formation of ASC aggregates (Masumoto *et al*, 1999; Lu *et al*, 2014). The caspase activation and recruitment domain (CARD) of ASC can then recruit caspase‐1 through CARD/CARD interactions, leading to the dimerization and autocleavage of caspase‐1, which converts pro‐IL‐1β and pro‐IL‐18 to the secreted forms of these cytokines. Caspase‐1 can also induce pyroptosis by activating pore‐forming proteins termed gasdermins. This causes the cell membrane to rupture and release cytokines, such as IL‐1β and IL‐18, and other molecules that promote inflammation to combat infection (reviewed (Rathinam *et al*, 2012; Lamkanfi & Dixit, 2014)).

At one time, it was thought that the sole function of TLR signalling in activating the NLRP3 inflammasome was to induce the synthesis of pro‐IL‐1β and to increase the expression of inflammasome components, such as NLRP3. However, it is now clear that TLR signalling is also required for the rapid (30‐60 min) phase of caspase‐1 activation, which is independent of the synthesis of pro‐IL‐1β or the increased expression of NLRP3 (Schroder *et al*, 2012; Fernandes‐Alnemri *et al*, 2013; Lin *et al*, 2014). Nevertheless, our understanding of how TLR signalling facilitates the rapid activation of the NLRP3 inflammasome is still rudimentary.

Here, we report a novel role for IKKβ in the formation of the NLRP3 inflammasome.

## Results

### Pharmacological inhibitors of IKKβ prevent the activation of caspase‐1

**Figure 1:**
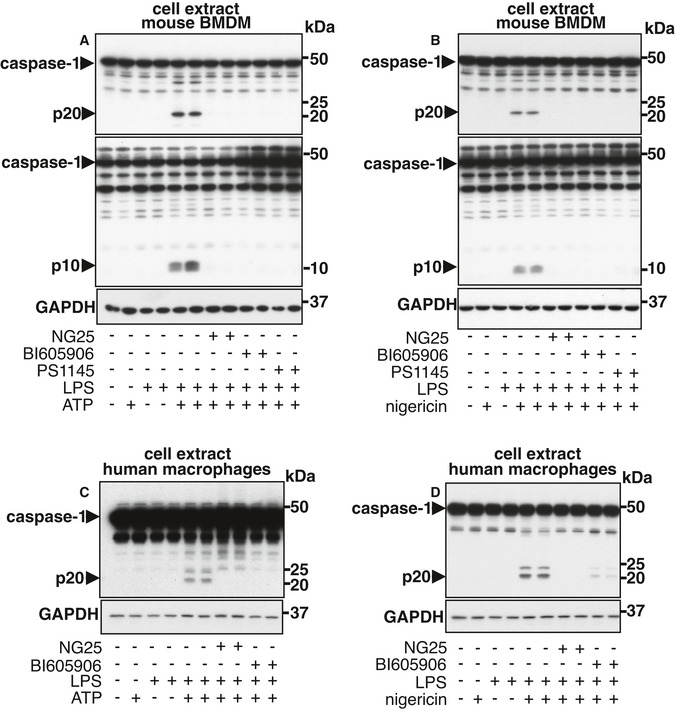
Inhibition of IKKβ or its activator TAK1 blocks the processing of caspase‐1 AWT BMDM were incubated for 1 h without (−) or with (+) the TAK1 inhibitor NG25 (2 µM), the IKKβ inhibitor BI605906 (5 µM) or the IKKβ inhibitor PS1145 (10 µM). The cells were then co‐stimulated for 30 min with 100 ng/ml LPS and 4 mM ATP. Cell lysates (10 µg protein) were denatured in SDS, subjected to SDS–PAGE and immunoblotted with the antibodies indicated.BAs in A, except that the cells were co‐stimulated for 1 h with 100 ng/ml LPS and 5 µM nigericin. Similar results were obtained in three independent experiments in A and B.C, DAs in A, B, except that primary human monocyte‐derived macrophages were used instead of mouse BMDM. Similar results were obtained in two independent experiments. Source data are available online for this figure.

**Figure EV1:**
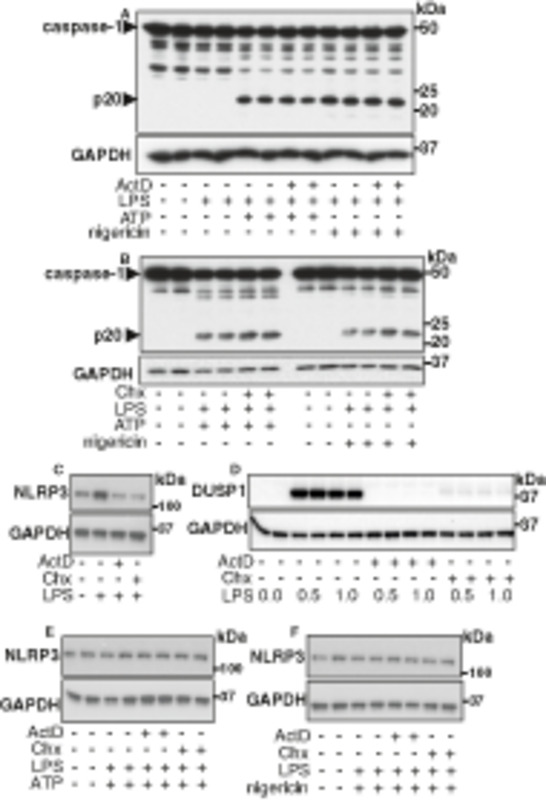
Inhibition of *de novo* transcription or protein synthesis does not affect the rapid activation of the NLRP3 inflammasome A, BWT BMDM were incubated for 30 min without (−) or with (+) 5 µg/ml actinomycin D (ActD) (A) or 10 µg/ml cycloheximide (Chx) (B). The cells were then co‐stimulated for 30 min with (+) or without (−) 100 ng/ml LPS and 4 mM ATP or for 1 h with 100 ng/ml LPS and 5 µM nigericin. Cell lysates (10 µg protein) were subjected to SDS–PAGE and immunoblotted with the antibodies indicated. Similar results were obtained in three independent experiments.CWT BMDM were incubated for 30 min without (−) or with (+) 5 µg/ml actinomycin D (ActD) or 10 µg/ml cycloheximide (Chx) and then stimulated for 4 h with (+) 100 ng/ml LPS or left unstimulated (−). Cell extract protein (10 µg) was subjected to SDS–PAGE and immunoblotted with anti‐NLRP3. GAPDH was used as a loading control. Similar results were obtained in two independent experiments.DAs in C, except that the cells were stimulated for the times indicated (hours) and immunoblotting was performed with anti‐DUSP1 (dual specificity phosphatase 1). Similar results were obtained in two independent experiments.E, FAs in A, B, except that immunoblotting was performed with anti‐NLRP3 and anti‐GAPDH. Similar results were obtained in two independent experiments. Source data are available online for this figure.

The co‐stimulation of bone marrow‐derived macrophages (BMDM) with the TLR4 activator lipopolysaccharide (LPS) and a second signal (ATP or nigericin) assembles the NLRP3 inflammasome leading to the activation of caspase‐1, which can be monitored by the appearance of the 20 and 10 kDa proteolytic fragments of caspase‐1 (hereafter called p20 and p10, respectively) (Fig 1A and B). In contrast, stimulation with LPS alone, or either ATP or nigericin alone, does not activate caspase‐1 (Fig 1A and B). This rapid activation of caspase‐1 is independent of *de novo* mRNA or protein synthesis, since prior treatment of the cells with actinomycin D (Fig EV1A) or cycloheximide (Fig EV1B) did not impair the activation of caspase‐1 induced by co‐stimulation with LPS and either ATP or nigericin. As expected, the same concentrations of actinomycin D or cycloheximide blocked the increased expression of NLRP3 induced by prolonged stimulation with LPS alone (Fig EV1C) and the rapid (0.5–1.0 h) induction of DUSP1 (dual specificity phosphatase 1) a protein that is not involved in activating the inflammasome (Fig EV1D). Co‐stimulation for 0.5 h with LPS and ATP or 1.0 h with LPS and nigericin did not increase the expression of NLRP3 and was unaffected by actinomycin D or cycloheximide (Fig EV1E and F).

**Figure EV2:**
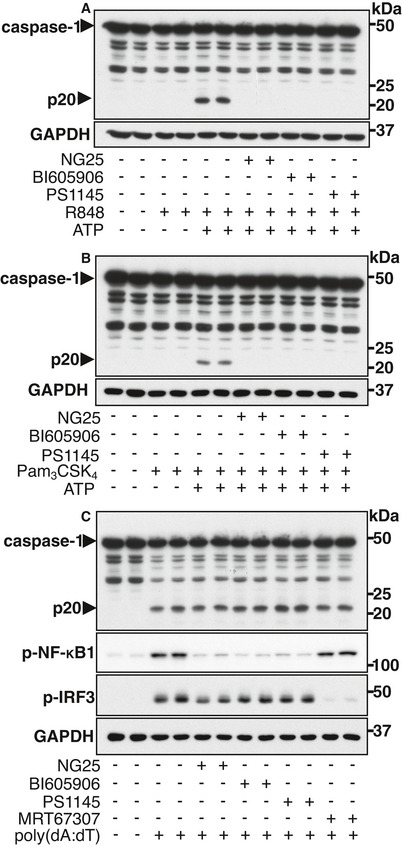
TAK1 or IKKβ inhibitors block activation of the NLRP3 inflammasome but not the AIM2 inflammasome BMDM were incubated for 1 h without (−) or with (+) 2 µM NG25, 5 µM BI605906 or 10 µM PS1145 and then stimulated for 30 min without (−) or with (+) 1 µg/ml R848 and 4 mM ATP. Cell lysates were subjected to SDS–PAGE and immunoblotted with the antibodies indicated. Similar results were obtained in two independent experiments.As in A, except that 1 µg/ml Pam_3_CSK_4_ was used instead of R848.As in A except that MRT67307 (2 µM), an inhibitor of the IKK‐related kinases TBK1 and IKKɛ was also included and the cells were stimulated by transfection for 2 h with 2 µg/ml poly(dA:dT) to activate the AIM2 inflammasome (+) or mock transfected (−). Similar results were obtained in two independent experiments. Source data are available online for this figure.

To investigate how co‐stimulation with LPS and either ATP or nigericin might activate the NLRP3 inflammasome, we studied the effects of many small molecule inhibitors of protein kinases that are known to become activated rapidly upon stimulation with LPS. Only three of these compounds, NG25 (Pauls *et al*, 2012), BI605906 (Clark *et al*, 2011) and PS1145 (Castro *et al*, 2003), suppressed the formation of p20 or p10 in mouse BMDM (Fig 1A and B) or in primary human macrophages (Fig 1C and D), or when LPS was replaced by the TLR7‐activating ligand R848 (Fig EV2A) or the TLR1/2‐activating ligand Pam_3_CSK_4_ (Fig EV2B).

BI605906 and PS1145 are structurally unrelated inhibitors of IKKβ and do not inhibit IKKα or the IKK‐related kinases (IKKɛ and TBK1). BI605906 is particularly selective (Clark *et al*, 2011). NG25 inhibits TAK1 (Dzamko *et al*, 2012), a protein kinase required to initiate the activation of IKKβ. Taken together, these results suggested that IKKβ activity was required for the rapid activation of the NLRP3 inflammasome.

We also investigated whether IKKβ was required to activate the AIM2 inflammasome because, like the NLRP3 inflammasome, the AIM2 inflammasome requires ASC to activate caspase‐1 (Srinivasula *et al*, 2002; Hornung *et al*, 2009). We found that the cytoplasmic delivery of poly(dA:dT) (a synthetic B‐form double‐stranded DNA that activates the AIM2 inflammasome) induced the processing of caspase‐1 to p20, but this was not prevented by either BI605906 or PS1145 (Fig EV2C), indicating that the effect of IKKβ was specific to the NLRP3 inflammasome.

### Genetic evidence that IKKβ is required to activate the NLRP3 inflammasome

**Figure 2:**
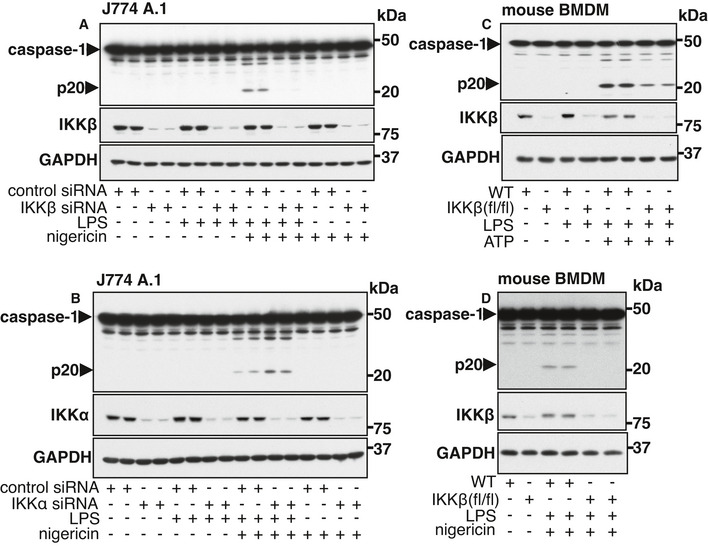
IKKβ but not IKKα is required for the activation of caspase‐1 A, BThe mouse macrophage J774 A.1 cell line was transfected with control siRNA or siRNA against IKKβ (A) or IKKα (B). 72 h post‐transfection, the cells were co‐stimulated for 60 min without (−) or with (+) 100 ng/ml LPS and/or 5 µM nigericin. Cell lysates (10 µg) were denatured in SDS, subjected to SDS–PAGE and immunoblotted with the antibodies indicated. Similar results were obtained in three (A) or two (B) independent experiments.C, DAs in (A, B) except that BMDM from IKKβ‐LysM‐Cre (flox/flox) (IKKβ (fl/fl)) or WT control mice were co‐stimulated without (−) or with (+) 100 ng/ml LPS and/or 4 mM ATP (C) or with 100 ng/ml LPS and 5 µM nigericin (D). Similar results were obtained in two independent experiments. Source data are available online for this figure.

To obtain independent evidence that IKKβ is required to activate the NLRP3 inflammasome, we reduced its level of expression in the J774 A.1 mouse macrophage cell line using RNA interference and showed that this treatment suppressed the formation of p20 induced by co‐stimulation with LPS and nigericin (Fig 2A). In contrast, reducing the expression of IKKα actually enhanced caspase‐1 processing (Fig 2B). Similar observations have been made in BMDM from knock‐in mice expressing a kinase‐inactive mutant of IKKα (Martin *et al*, 2014). Thus, IKKα restricts activation of the NLRP3 inflammasome.

We next studied the activation of the NLRP3 inflammasome in BMDM from IKKβ‐LysM‐Cre (flox/flox) mice, in which the expression of IKKβ is reduced, but not abolished (Fig 2C and D). Consistent with the RNA interference data, the formation of p20 induced by co‐stimulation with LPS and ATP (Fig 2C) or LPS and nigericin (Fig 2D) was reduced in primary BMDM from these mice.

### IKKβ activity is required for the activation of gasdermin D

**Figure 3:**
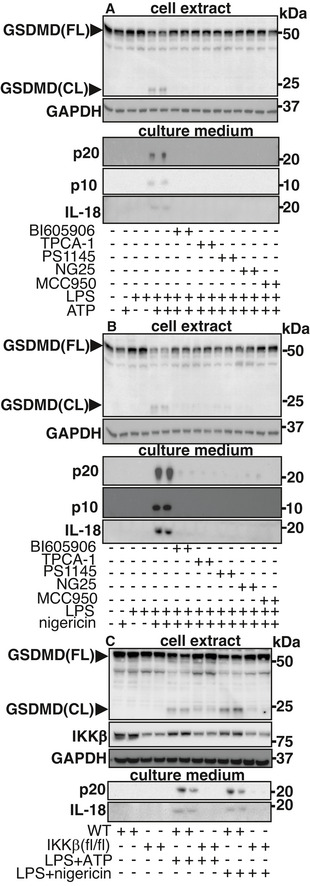
IKKβ activity is required for the cleavage of gasdermin D and the secretion of caspase‐1 fragments and IL‐18§ A, BWT BMDM were incubated for 1 h without (−) or with (+), the IKKβ inhibitors BI605906 (5 µM), TPCA‐1 (5 µM) or PS1145 (10 µM), the TAK1 inhibitor NG25 (2 µM) or the NLRP3 inhibitor MCC950 (1 µM). The cells were then co‐stimulated for 30 min without (−) or with (+) 100 ng/ml LPS and/or 4 mM ATP (A) and/or 5 µM nigericin (B). The cell culture medium was removed and the cells lysed. Protein in the culture medium was precipitated (see Materials and Methods), dissolved in SDS and subjected to SDS–PAGE, along with cell lysates. After transfer to PVDF membranes, immunoblotting was performed with antibodies recognizing both full length (FL) and cleaved (CL) gasdermin D (GSDMD), the p20 and p10 fragments of caspase‐1 and IL‐18. Similar results were obtained in two independent experiments.CAs in A, except that iBMDM from IKKβ‐CXCR3‐Cre (flox/flox) (IKKβ (fl/fl)) and control WT cells were used. Similar results were obtained in three independent experiments. Source data are available online for this figure.

One role of activated caspase‐1 is to cleave gasdermin D to the active N‐terminal fragment GSDMD(NT), which triggers pyroptosis (Kayagaki *et al*, 2015; Shi *et al*, 2015). GSDMD(NT) is a pore‐forming protein, which is required for the secretion of p20, p10 and IL‐18 (Broz & Dixit, 2016). We found that GSDMD was cleaved‡ during the rapid activation of the NLRP3 inflammasome and blocked by any of three structurally unrelated inhibitors of IKKβ or the TAK1 inhibitor NG25 (Fig 3A and B) and reduced in immortalized (iBMDM) from IKKβ‐CXCR3‐Cre (flox/flox) mice (Fig 3C). Consistent with the activation of gasdermin D, we found that co‐stimulation with LPS and ATP, or LPS and nigericin, induced the secretion of p20, p10 and IL‐18, which was prevented by the inhibition of IKKβ (Fig 3A and B) and reduced in IKKβ‐deficient iBMDM (Fig 3C). MCC950, an inhibitor of the NLRP3 inflammasome, which binds directly to NLRP3 (Coll *et al*, 2019; Tapia‐Abellan *et al*, 2019; Vande Walle *et al*, 2019), also prevented the secretion of p20, p10 and IL‐18 (Fig 3A and B), as expected. Importantly, the finding that the secretion of p20 and p10 was prevented by IKKβ inhibition (Fig 3A–C) excludes the possibility that IKKβ inhibition suppresses the levels of p20 and p10 in the cell extracts by accelerating their secretion.

### IKKβ does not regulate the activation of the NLRP3 inflammasome via IKK‐related kinases, Tpl2 or LRRK2

**Figure EV3:**
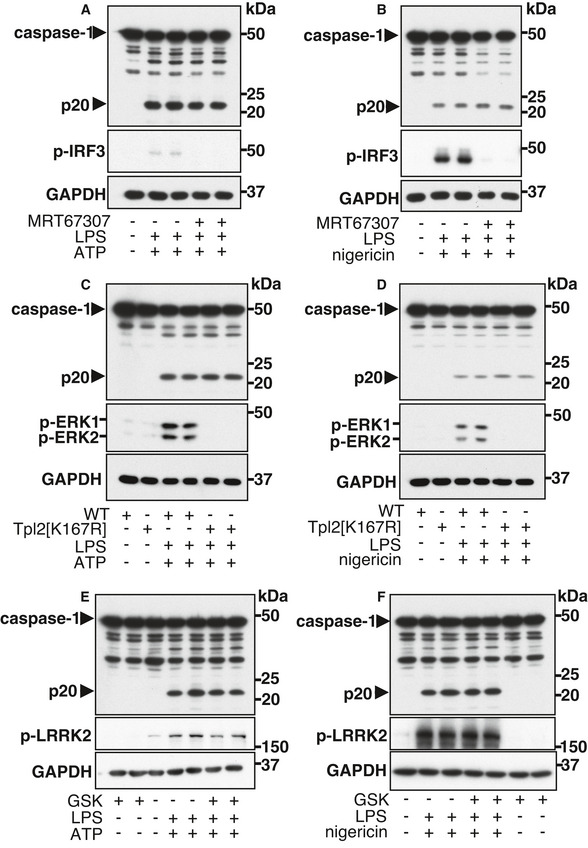
The inhibition of protein kinases activated by IKKβ does not affect the activation of caspase‐1 induced by the formation of the NLRP3 inflammasome A, BWT BMDM were incubated for 1 h without (−) or with (+) 2 µM of the TBK1/IKKɛ inhibitor MRT67307 and then stimulated for 30 min without (−) or with (+) 100 ng/ml LPS and/or 4 mM ATP (A) or 5 µM nigericin (B). Cell lysates (10 µg protein) were subjected to SDS–PAGE and immunoblotted with the antibodies indicated. Similar results were obtained in three independent experiments.C, DAs in A, B, except that BMDM from knock‐in mice expressing the catalytically inactive Tpl2[K167R] mutant or WT control mice were used. Similar results were obtained in two independent experiments.E, FAs in A, B except that the cells were incubated without (−) or with (+) 1 µM GSK2578215A (GSK), an inhibitor of LRRK2. Similar results were obtained in three independent experiments. Source data are available online for this figure.

IKKβ activates several other protein kinases, namely, the IKK‐related kinases (TBK1 and IKKɛ) (Clark *et al*, 2011), Tpl2 (also called COT (Cancer Osaka Thyroid)) (Beinke *et al*, 2004; Waterfield *et al*, 2004) and LRRK2 (Dzamko *et al*, 2012). It was therefore possible that IKKβ activates the NLRP3 inflammasome indirectly by first activating another protein kinase(s). To investigate the possible involvement of the IKK‐related kinases we used MRT67307, a potent inhibitor of TBK1 and IKKɛ (Clark *et al*, 2011). This compound blocked the TBK1‐catalysed phosphorylation of IRF3 (interferon regulatory factor 3), as expected, (Fig EV3A and B) (Clark *et al*, 2011), but not the processing of caspase‐1 induced by co‐stimulation with LPS and ATP (Fig EV3A), or LPS and nigericin (Fig EV3B).

To investigate whether Tpl2 had a role in activating the NLRP3 inflammasome, we performed experiments with BMDM from knock‐in mice expressing the catalytically inactive Tpl2[K167R] mutant. We found that the formation of p20 induced by co‐stimulation with LPS and ATP (Fig EV3C) or LPS and nigericin (Fig EV3D) was similar in BMDM from Tpl2[K167R] mice and wild‐type mice. A major role of Tpl2 is to activate the MAP kinase kinases (MEK1 and MEK2), which phosphorylate ERK1 and ERK2. As expected, the rapid LPS‐stimulated phosphorylation of ERK1 and ERK2 was suppressed in BMDM from Tpl2[K167R] mice (Fig EV3C and D).

The IKK family members phosphorylate LRRK2 at Ser910 and Ser935 *in vitro* and the phosphorylation of Ser935 is increased by stimulating BMDM with TLR‐activating ligands (Dzamko *et al*, 2012). We found that the formation of p20 induced by co‐stimulation with LPS and ATP (Fig EV3E) or LPS and nigericin (Fig EV3F) was unaffected by GSK2578215A, a potent and specific LRRK2 inhibitor (Reith *et al*, 2012). GSK2578215A did, however, suppress the basal, but not the LPS‐enhanced phosphorylation of LRRK2 at Ser935 (Dzamko *et al*, 2012) (Fig EV3E and F).

### IKKβ is required for the oligomerization of ASC

**Figure 4:**
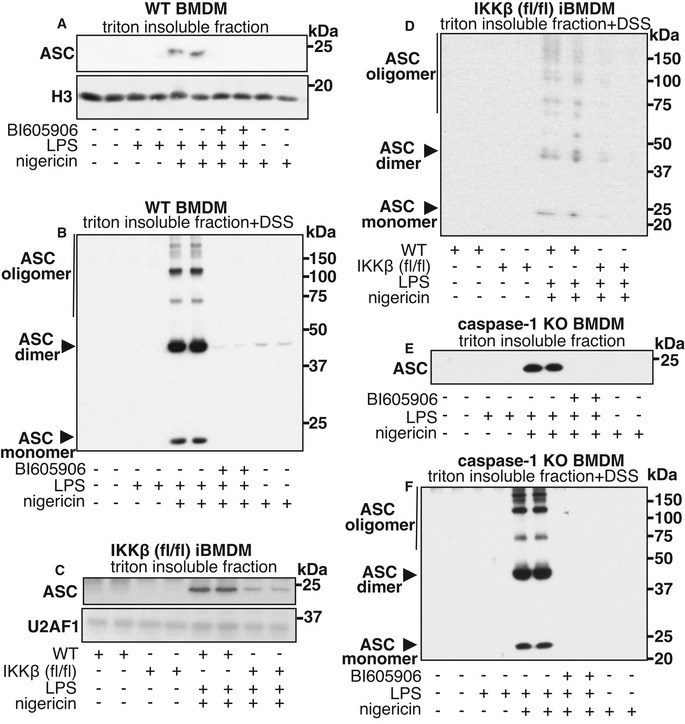
Oligomerization of ASC requires IKKβ activity and is independent of caspase‐1 AWT BMDM were incubated for 1 h without (−) or with (+) 5 µM BI605906 and then stimulated for 1 h with (+) 100 ng/ml LPS and/or 5 µM nigericin or left unstimulated (‐). The cells were lysed in buffer containing 1% (v/v) Triton X‐100, and the Triton X‐100‐soluble and Triton X‐100‐insoluble fractions were prepared as in Materials and Methods. The Triton X‐100‐insoluble fraction was dissolved SDS subjected to SDS–PAGE and immunoblotted with the antibodies indicated. The nuclear protein Histone H3 (H3) was used as a loading control in the Triton X‐100 insoluble fraction. Similar results were obtained in three independent experiments.BAs in A, except that the Triton X‐100‐insoluble fraction was first subjected to crosslinking for 45 min at 37°C with 2.0 mM DSS.C, DAs in A, B except that iBMDM from IKKβ‐CXCR3‐Cre (flox/flox) mice (IKKβ (fl/fl)) were used. The U2 small nuclear RNA auxiliary factor 1 (U2AF1) was used as a loading control. Similar results were obtained in two independent experiments.E, FAs in A, B except that BMDM from caspase‐1 KO mice were used. Similar results were obtained in two independent experiments. Source data are available online for this figure.

**Figure EV4:**
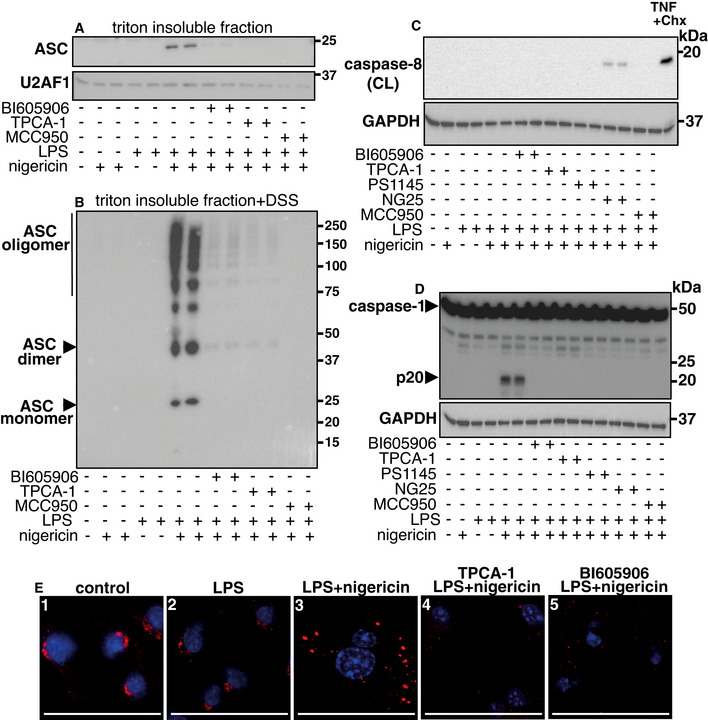
IKKβ stimulates the formation of the NLRP3 inflammasome and its activation is independent of caspase8 WT BMDM were incubated for 1 h without (−) or with (+) 5 μM BI605906, 5 μM TPCA‐1 or 1 μM MCC950. The cells were then stimulated for 1 h with (+) 100 ng/ml LPS and/or 5 μM nigericin, or left unstimulated (−). After cell lysis in the presence of 1% (v/v) Triton X‐100, the Triton X‐100‐insoluble fractions were prepared as in Fig 4, denatured in SDS, subjected to SDS–PAGE and immunoblotted with anti‐ASC or with an antibody against the U2 small nuclear RNA auxiliary factor 1 (U2AF1) as a loading control.As in A, except that the Triton X‐100‐insoluble fraction was first crosslinked by incubation for 45 min at 37°C with 2.0 mM DSS. (A, B) Similar results were obtained in two independent experiments.WT BMDM were incubated for 1 h with (+) or without (−) the IKKβ inhibitors BI605906 (5 μM), TPCA‐1 (5 μM) or PS1145 (10 μM), the TAK1 inhibitor NG25 (2 μM) or the NLRP3 inflammasome inhibitor MCC950 (1 μM). The cells were then stimulated with LPS (100 ng/ml) and/or 5 μM nigericin (+) or left unstimulated (‐). Cells were stimulated with TNF (10 ng/ml) and cycloheximide (Chx) (10 μg/ml), which was used as a positive control for a signal generating cleaved (CL) caspase‐8. Cells were lysed, cell extracts (10 µg protein) were subjected to SDS–PAGE and immunoblotted with antibodies recognizing caspase‐8(CL) (the cleavage product of caspase‐8) and GAPDH.As in C, except that the samples were immunoblotted for full length caspase‐1 (caspase‐1), the p20 fragment of caspase‐1 (p20) and GAPDH. Similar results were obtained in two independent experiments.WT BMDM were stimulated for 4 h without (Panel 1, control) or with (Panels 2–5) 100 ng/ml LPS and then incubated for 1 h without (Panels 1–3) or with 5 µM TPCA‐1(Panel 4) or 5 µM BI605906 (Panel 5) and then stimulated for 60 min with 5 µM nigericin (Panels 3–5) or without nigericin (Panels 1 and 2). The cells were fixed and processed for immunofluorescence using a rabbit polyclonal antibody against TGN38, which was visualized using a secondary antibody (red). Nuclei were counterstained with DAPI (blue). Images were acquired by sequential laser scanning on the confocal microscope. Similar results were obtained in three independent experiments, and representative images are shown. Data information: In all panels, scale bar = 50 µm. Source data are available online for this figure.

ASC has been reported to form Triton X‐100‐resistant filamentous aggregates during the activation of the NLRP3 inflammasome, an event that occurs prior to the activation of caspase‐1 (Masumoto *et al*, 1999; Lu *et al*, 2014). ASC is not detectable in the Triton X‐100‐insoluble fraction of unstimulated cells or in cells stimulated with LPS or nigericin alone, but significant amounts of ASC appear in this fraction after co‐stimulation with LPS and nigericin (Fig 4A). Cross‐linking of the Triton X‐100‐insoluble fraction with disuccinimidyl suberate (DSS), prior to denaturation in SDS, caused ASC to migrate as a dimer and form even larger aggregates (Fig 4B). The appearance of ASC in the Triton X‐100‐insoluble fraction was prevented by treatment with the IKKβ inhibitor BI605906 (Fig 4A and B) or with a different IKKβ inhibitor TPCA‐1 or the NLRP3 inhibitor MCC950 (Fig EV4A and B). Similar results were obtained in iBMDM from IKKβ‐deficient mice in which the translocation of ASC to the Triton X‐100‐insoluble fraction, and its oligomerization was reduced (Fig 4C and D).

The translocation of ASC to the Triton X‐100‐insoluble fraction and the formation of ASC oligomers was unimpaired in macrophages from caspase‐1 knock‐out (KO) mice, but prevented by the inhibition of IKKβ (Fig 4E and F). Taken together, these results indicate that IKKβ exerts its effects on ASC oligomerization prior to the recruitment of caspase‐1 into the NLRP3 inflammasome.

### IKKβ‐dependent co‐localization of NLRP3 with Trans‐Golgi Network (TGN) 38 protein

**Figure 5:**
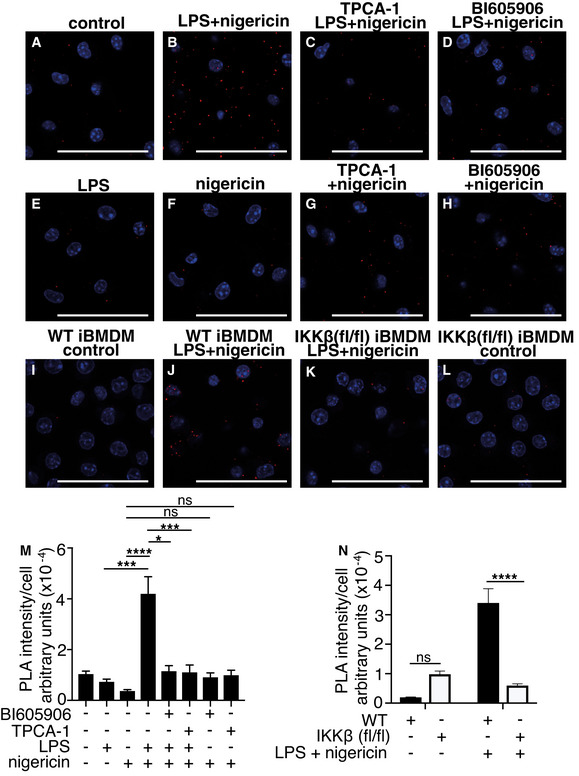
IKKβ induce interaction between NLRP3 and TGN38 A–HWT BMDM were incubated for 1 h without or with the IKKβ inhibitors TPCA‐1 (5 µM) (C, G) or BI605906 (5 µM) (D, H). The cells were then stimulated for 1 h with 100 ng/ml LPS (E) or 5 µM nigericin (F, G, H) or co‐stimulated with 100 ng/ml LPS and 5 µM nigericin (B, C, D) or left unstimulated (control) (A). The cells were fixed and processed for PLA using a rabbit polyclonal antibody against TGN38 and a mouse monoclonal antibody against NLRP3. A PLA signal is seen only if the NLRP3 antibody and the TGN38 antibody come into close proximity. (A‐H) The panel images show the PLA signal (red) and DAPI staining for nuclei (blue). Images were acquired by sequential laser scanning on a confocal microscope. Similar results were obtained in three independent experiments, and representative images are shown. Scale bar = 50 µm.I–LAs in A‐H, except that IKKβ inhibitors were omitted and iBMDM from IKKβ‐CXCR3‐Cre (flox/flox) (IKKβ (fl/fl)) or WT mice were co‐stimulated for 1 h with 100 ng/ml LPS and 10 µM nigericin or left unstimulated (control). Scale bar = 50 µm.MThe graph shows the mean ± SEM of the average intensity of the PLA signal per cell. The data were acquired from 20 different fields in A–H and from two separate experiments. The statistical significance was calculated using Kruskal–Wallis test with Dunn’s multiple comparison; *denotes *P* < 0.05, ***P < 0.001 and ****P < 0.0001; ns, not‐significant.NAs in M, except that the PLA signal per cell was acquired from 10 different fields in I–L, and statistical significance was calculated using two‐way ANOVA with Sidak’s multiple comparison; ****P < 0.0001; ns, not‐significant. Similar results were obtained in a second independent experiment. The graph shows the mean ± SEM. Source data are available online for this figure.

The recruitment of NLRP3 to the TGN is an early event leading to the aggregation of NLRP3 and the formation and activation of the NLRP3 inflammasome (Chen & Chen, 2018). To investigate whether IKKβ had a role in this process, we used a proximity ligation assay (PLA) to investigate whether IKKβ activity was required to bring NLRP3 into proximity with TGN38, a marker of the TGN. The experiment employed two different antibodies, one recognizing NLRP3 and the other TGN38, and a positive PLA signal was seen only if NLRP3 and TGN38 interacted. The co‐stimulation of primary macrophages with LPS and nigericin did indeed bring NLRP3 into proximity with TGN38 compared to unstimulated cells (Fig 5A and B), which was prevented if IKKβ was inhibited (Fig 5C and D). In contrast, stimulation with LPS or nigericin in the absence (Fig 5E and F) or presence of IKKβ inhibitors (Fig 5G and H) had no effect. Similar results were obtained in immortalized macrophages (iBMDM) where co‐stimulation with LPS and nigericin brought NLRP3 and TGN38 into proximity in WT (Fig 5I and J) but not IKKβ‐deficient cells (Fig 5K and L). The quantitation of the results obtained from many fields in primary (Fig 5M) and immortalized (Fig 5N) BMDM is also presented.

### IKKβ enhances TGN38 dispersal during co‐stimulation with LPS and nigericin

The dispersion of the TGN induced by nigericin is thought to enable the formation of ionic bonding between the polybasic region of NLRP3 and negatively charged phosphatidylinositol‐4‐phosphate molecules that have been exposed by dispersal of the TGN, causing NLRP3 to aggregate into multiple puncta and induce formation and activation of the inflammasome (Chen & Chen, 2018). Puncta formation in primary macrophages has been studied previously by prolonged stimulation with LPS to induce high levels of NLRP3 expression, followed by stimulation with nigericin. This procedure produces a strong signal, but does not permit the individual roles of LPS and nigericin in puncta formation to be investigated. We therefore studied the role of IKKβ activity in puncta formation during the rapid transcription‐independent activation of the NLRP3 inflammasome.

**Figure 6:**
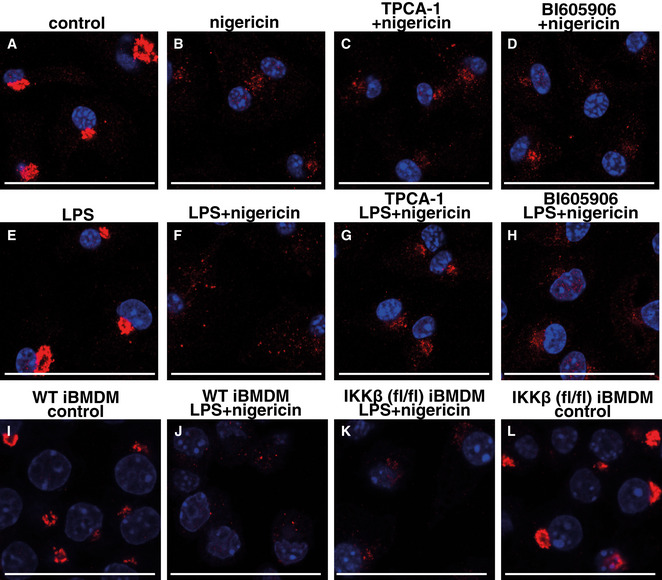
IKKβ induces the formation of brighter TGN38‐positive puncta after co‐stimulation with LPS and nigericin A–HThe experiments were performed as in Fig 5A–H, except that the cells were fixed and prepared for immunofluorescence staining using a rabbit polyclonal TGN38 antibody, which was visualized using a secondary antibody (red). Nuclei were counterstained with DAPI (blue). Images were acquired by sequential laser scanning on a confocal microscope. Similar results were observed in many fields and obtained in three independent experiments. Representative images are shownI–LAs in A‐H, except that iBMDM from IKKβ‐CXCR3‐Cre (flox/flox) (IKKβ (fl/fl)) or WT mice were co‐stimulated for 1 h with 100 ng/ml LPS and 10 µM nigericin or left unstimulated. Similar results were obtained in two independent experiments. Data information: In all panels, scale bar = 50 µm.

The TGN is intact in unstimulated primary BMDM with TGN38 displaying the expected perinuclear location (Fig 6A). Treatment with nigericin alone caused dispersion of the TGN and the formation of multiple small TGN38‐positive puncta, which mostly retained a perinuclear location (Fig 6B). The effect of nigericin was unaffected by IKKβ inhibition (Fig 6C and D). LPS alone had no effect compared to unstimulated cells (compare Fig 6E with 6A) but co‐stimulation with LPS and nigericin consistently generated puncta that were located more distantly from the perinuclear region (Fig 6F) than those generated by nigericin alone (Fig 6B). IKKβ inhibitors prevented the effect of co‐stimulation, the location of the puncta being similar to those seen after stimulation with nigericin alone (compare Fig 6G and H with Fig 6B). Similar results were observed in immortalized BMDM (iBMDM) from WT mice, where co‐stimulation with LPS and nigericin caused the appearance of puncta distant from the perinuclear region (Fig 6I and J), which were not observed in iBMDM from IKKβ‐deficient (fl/fl) mice (Fig 6K and L).

### Caspase‐8 is not activated during the rapid activation of the NLRP3 inflammasome

A much slower spontaneous activation of the NLRP3 inflammasome taking place in the absence of any TLR‐activating ligand has been observed in TAK1‐deficient or IKKβ‐deficient BMDM and in WT BMDM treated with the TAK1 inhibitor 5Z‐7‐oxozeanol or the IKKβ inhibitor ML120B. In this pathway, which only begins after about 4 h, inflammasome activation is a consequence of the failure to maintain a low basal level of TNF‐signalling, enabling RIPK1 to activate cell death pathways leading to the activation of caspase‐8 (Greten *et al*, 2007; Sanjo *et al*, 2019; Malireddi *et al*, 2020). We found that the rapid activation of the NLRP3 inflammasome did not induce any activation of caspase‐8 (Fig EV4C lanes 5 and 6) although it stimulated formation of the p20 fragment of caspase‐1 as expected (Fig EV4D). In contrast, incubation of BMDM with the TAK1 inhibitor NG25, followed by co‐stimulation with LPS and nigericin, did induce caspase‐8 activation (Fig EV4C, lanes 13 and 14), but the formation of p20 was blocked (Fig EV4D). Incubation with three structurally unrelated IKKβ inhibitors, followed by co‐stimulation with LPS and nigericin, did not induce any activation of caspase‐8 (Fig EV4C) and prevented the formation of p20 induced by co‐stimulation (Fig EV4D). Taken together, our results exclude the involvement of caspase‐8 in the rapid activation of the NLRP3 inflammasome.

### IKKβ is required for the formation of TGN38‐positive puncta induced by prolonged stimulation with LPS followed by stimulation with nigericin

We also studied the formation of the NLRP3 inflammasome in macrophages stimulated with LPS for several hours, enabling IKKβ to mediate its transcriptional effects, such as the increased expression of NLRP3 (Fig EV1C), as well its transcription‐independent effects. Subsequent stimulation with nigericin induced the formation of TGN38‐positive puncta (Fig EV4E, compare panels 1–3), but not if IKKβ inhibitors were added after stimulation with LPS but prior to stimulation with nigericin (Fig EV4E, panels 4 and 5). The TGN‐positive puncta formed (Fig EV4E, panel 3) were much larger than those produced during the rapid activation of the NLRP3 inflammasome (Fig 6F). This difference may be explained by the enhanced expression of NLRP3 during prolonged stimulation with LPS (Fig EV1C) causing more NLRP3 molecules to interact with one another and so induce the coalescence of many small TGN38‐positive punta.

### IKKβ activity is required to activate the NLRP3 inflammasome, even after the transcriptional upregulation of NLRP3

**Figure EV5:**
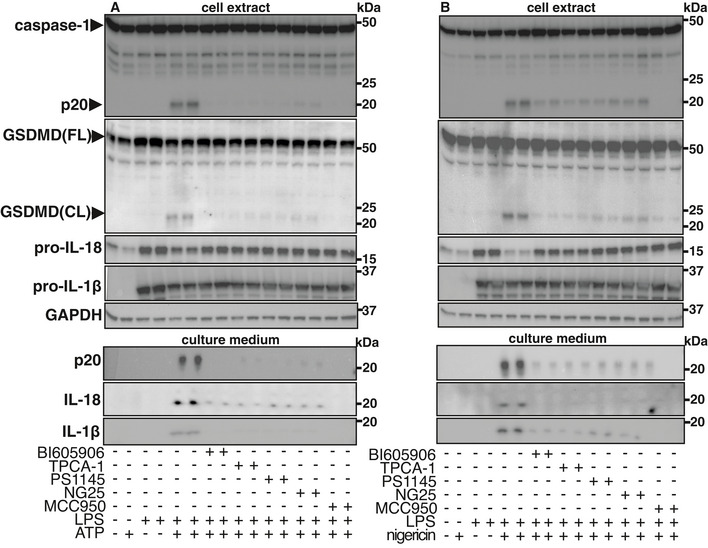
Inhibition of IKKβ and TAK1 prevents inflammasome activation after transcriptional upregulation of NLRP3 WT BMDM were either incubated for 4 h (+) with LPS (100 ng/ml) or left untreated (−) and then incubated for 1 h without (−) or with (+) BI605906 (5 µM) or TPCA‐1 (5 µM) or PS1145 (10 µM) or NG25 (2 µM) or MCC950 (1 µM). The cells were then stimulated for 30 min with (+) 4 mM ATP, the culture medium removed, the cells lysed and the cell lysates (10 µg protein) denatured in SDS. The protein in the cell culture medium was precipitated (see Materials and Methods) and also dissolved in SDS. The cell extracts and samples from the cell culture medium were subjected to SDS–PAGE, transferred to PVDF membranes and immunoblotted with antibodies recognizing full length (FL) or cleaved (CL) gasdermin D (GSDMD), the p20 fragment of caspase‐1, IL‐18 and IL‐1β.As in A, except that the cells were stimulated with nigericin (5 µM, 1 h) instead of ATP. Similar results were obtained in two independent experiments. Source data are available online for this figure.

A major role of the NLRP3 inflammasome is to stimulate the cleavage of pro‐IL‐1β and pro‐IL‐18 and the secretion of these cytokines (Rathinam *et al*, 2012). Only the secretion of IL‐18 could be studied during the rapid activation of the NLRP3 inflammasome because pro‐IL‐1β is not expressed under basal conditions. As expected, prolonged stimulation of BMDM with LPS not only increased the expression of NLRP3 (Fig EV1C) and pro‐IL‐18, but also the expression of pro‐IL‐1β. Prolonged stimulation with LPS, followed by stimulation with ATP or nigericin also led to the formation of p20 and its secretion, and to the generation of the active N‐terminal cleavage product of gasdermin D (GSDMD(CL)¶) (Fig EV5A and B). The secretion of IL‐1β and IL‐18 (Fig EV5A and B) and the formation of p20 and GSDMD(CL)^¶^ were suppressed by the inhibition of IKKβ or TAK1 or by the NLRP3 inhibitor MCC950 (Fig EV5A and B). These results were similar to those observed when studying the rapid activation of the NLRP3 inflammasome.

## Discussion

Understanding how inflammasomes are activated is of considerable importance, given their pivotal role in protection against microbial infection and in the pathogenesis of inflammatory diseases (Guo *et al*, 2015). Here, we present pharmacological and genetic evidence that IKKβ is essential for the rapid formation of the NLRP3 inflammasome and subsequent activation of caspase‐1 and gasdermin D and hence the secretion of IL‐18 in BMDM (Figs 1, 2, 3). The IKKβ substrates whose phosphorylation is needed to activate the NLRP3 inflammasome are distinct from other known physiological substrates of this protein kinase, which include the IKK‐related kinases (TBK1 and IKKɛ), Tpl2 and LRRK2 (Fig EV3). An involvement of the transcription factors NF‐κB and IRF5, which are activated by IKKβ‐dependent phosphorylation events (see Introduction), is also excluded because the rapid activation of the NLRP3 inflammation does not require *de novo* gene transcription (Fig EV1).

It has been a mystery as to why the rapid activation of the NLRP3 inflammasome requires two signals, signal 1 frequently being a TLR‐activating ligand, such as LPS, and signal 2 a variety of structurally unrelated molecules, including extracellular ATP and nigericin. It is established that a key role of signal 2 is to trigger the disassembly of the Trans‐Golgi Network (TGN), the dispersed TGN then acting as a scaffold for the recruitment of NLRP3, which interacts with the phosphatidylinositol 4‐phosphate (PtdIns4P) exposed on the surface of the dispersed TGN (Chen & Chen, 2018). Here, we have established that a key role of signal 1 is to activate IKKβ, but the key substrates of IKKβ in this pathway whose phosphorylation stimulates an interaction between NLRP3 and the dispersed TGN have yet to be identified. Proteins whose phosphorylation facilitates the interaction of NLRP3 with PtdIns4P or a PtdIns4P‐binding protein(s) would be potential candidates, but PtdIns4P may not be the only molecule that is essential for the formation of the NLRP3 inflammasome. The role of IKKβ could therefore be PtdIns4P‐independent. However, the possibility that IKKβ is required for the nigericin‐induced dispersion of the TGN has been excluded. Importantly, the requirement for IKKβ provides a unifying mechanism that can explain why the rapid activation of the NLRP3 inflammasome and caspase‐1 is impaired to varying degrees in macrophages deficient in other proteins that are needed for, or contribute to the TLR‐dependent activation of IKKβ, such as IRAK4, IRAK1 (Fernandes‐Alnemri *et al*, 2013; Lin *et al*, 2014), IRAK1/IRAK2 (Martin *et al*, 2014) or the components of LUBAC (Rodgers *et al*, 2014; Gurung *et al*, 2015).

The activation of the AIM2 inflammasome, which does not induce the formation of TGN38‐positive puncta (Chen & Chen, 2018), is also unaffected by the inhibition of IKKβ, even though, like the NLRP3 inflammasome, activation of the AIM2 inflammasome leads to the oligomerization of ASC and the activation of caspase‐1 (Fig EV2C). It has been reported that activation of the AIM2 and NLRP3 inflammasomes is impaired in BMDM from IKKɛ deficient mice and that IKKɛ exerts these effects by phosphorylating ASC (Martin *et al*, 2014). Here, we found that the TBK1/IKKɛ inhibitor MRT67307 did not affect activation of the AIM2 and NLRP3 inflammasomes, although MRT67307 prevented the TBK1/IKKɛ‐catalysed phosphorylation of IRF3 at Ser396 as expected (Fig EV3A and B).

Although the present study was largely focused on the rapid activation of the NLRP3 inflammasome that is independent of *de novo* transcription or translation, we also performed experiments in which BMDM were first stimulated for 4 h with LPS to induce the transcriptional upregulation of NLRP3 and then stimulated for 1 h with nigericin in the continued presence of LPS, to activate the NLRP3 inflammasome. The inhibition of IKKβ prior to stimulation with nigericin prevented the activation of caspase‐1 and gasdermin D, as well as the secretion of IL‐18 and IL‐1β (Fig EV5). These experiments indicate that, following the LPS‐stimulated transcriptional upregulation of NLRP3 and IL‐1β, IKKβ activity is still needed to activate caspase‐1 and gasdermin D, presumably acting via the same mechanism that mediates the rapid activation of the NLRP3 inflammasome.

After submission of this manuscript, a paper was published reporting that pharmacological inhibition of IKKβ prevents the nigericin‐induced secretion of IL‐1β in the transformed human monocyte cell line THP1, in which pro‐IL‐1β expression had been induced by prolonged stimulation with phorbol myristate acetate (PMA) (Unterreiner *et al*, 2021). In the absence of PMA, prolonged stimulation with nigericin in the presence of a caspase‐1 inhibitor induced the formation of ASC specks (a readout of NLRP3 assembly) which was not blocked by IKKβ inhibition, and nor did IKKβ inhibition prevent the nigericin‐induced formation of caspase‐1 p20. Instead, IKKβ inhibition was reported to prevent the nigericin‐induced reduction in the level of pro‐caspase‐1. It was concluded that IKKβ inhibition dampens the nigericin‐induced activation of the NLRP3 inflammasome but the underlying molecular mechanism was not investigated (Unterreiner *et al*, 2021). In contrast, our paper was largely focused on studying the rapid transcription‐independent activation of the NLRP3 inflammasome in primary macrophages, which requires co‐stimulation with both a TLR ligand and nigericin, neither agonist alone having any effect (Figs 1, 2, 3). We establish using IKKβ‐deficient cells and IKKβ inhibitors that LPS‐stimulated activation of IKKβ is required for NLRP3 inflammasome assembly (Fig 4) and hence is also required for subsequent caspase‐1 activation and caspase‐1 p20 formation. We also show that IKKβ activity is required for the interaction of NLRP3 with the dispersed TGN, which is thought to be a key event in NLRP3 inflammasome assembly (Chen & Chen, 2018). However, our results do not exclude the possibility that IKKβ might contribute to caspase‐1 activation by an additional mechanism that is unrelated to its role in promoting NLRP3 assembly.

## Materials and Methods

### Inhibitors, agonists and chemicals

The sources of the TAK1 inhibitor NG25 (Dzamko *et al*, 2012; Tan *et al*, 2015), the IKKβ inhibitor BI605906 (Clark *et al*, 2011), the TBK1/IKKɛ inhibitor MRT67307 (Clark *et al*, 2011) and the LRRK2 inhibitor GSK2578215A (Reith *et al*, 2012) have been described. The IKKβ inhibitors PS1145 (Castro *et al*, 2003) and TPCA‐1 (Podolin *et al*, 2005) were from Sigma and Calbiochem, respectively. The NLRP3 inhibitor MCC950 (Coll *et al*, 2015) was obtained from Selleckchem. The TLR ligands Pam_3_CSK_4_ and R848 and the inflammasome agonists ATP, nigericin and poly(dA:dT) were from InvivoGen. LPS (lipopolysaccharide; *Escherichia coli* 055:B5) was from Alexis Biochemicals (ALX‐581‐001). A stock ATP solution (200 mM) was prepared in endotoxin‐free water (Sigma), and the pH of the solution was adjusted to 7.4 using NaOH. Actinomycin D and cycloheximide were from Sigma, disuccinimidyl suberate (DSS) from Thermo Fisher Scientific and murine TNF‐α (315‐01a) from PeproTech.

### Antibodies

Rabbit monoclonal antibodies recognizing p105/NF‐κB1 phosphorylated at Ser933 (18E6), ERK1 and ERK2 phosphorylated at the Thr‐Glu‐Tyr motif in the activation loop (D13.14.4E), IRF3 phosphorylated at Ser396 (4D4G), the cleaved form of caspase‐8 (D5B2) and all forms of glyceraldehyde 3‐phosphate dehydrogenase (GAPDH) (14C10) were from Cell Signalling Technology (CST). Rabbit polyclonal antibodies recognizing all forms of IKKα (2682, CST) and IKKβ (2684, CST), all forms of mouse IL‐18 (5180R from BioVision), U2 small nuclear RNA auxilliary Factor 1 (HPA044833 from Sigma), the p10 fragment of mouse caspase‐1 (M20 from Santa Cruz technologies) and histone 3 (H3) (ab18521 from Abcam) were purchased from the companies indicated in parentheses. A rabbit polyclonal antibody recognizing all forms of ASC (AL177), mouse monoclonal antibodies recognizing the p20 fragment of mouse caspase‐1 and full length mouse caspase‐1(Casper‐1), human caspase‐1 (Bally‐1) and all forms of NLRP3 (cryo‐2), as well as a guinea pig polyclonal antibody recognizing all forms of mouse gasdermin D (IN110) were from Adipogen. Mouse monoclonal antibodies recognizing all forms of IKKβ (10AG2 from Merck‐Millipore) and DUSP1 (E‐6 from Santa Cruz technologies) were purchased from the suppliers indicated in parentheses. A goat polyclonal antibody against mouse IL‐1β was from R&D (AF‐401). The rabbit and mouse secondary antibodies conjugated to horseradish peroxidase (HRP) were from CST, whereas the HRP‐conjugated guinea pig secondary antibody was from Abcam. A rabbit monoclonal antibody recognizing LRRK2 phosphorylated at Ser935 was provided by Dario Alessi, MRC Protein Phosphorylation and Ubiquitylation Unit, University of Dundee, UK. The TGN38 antibody, specifically recognizing mouse TGN38 was a gift from Matthew Seaman, Cambridge Institute for Medical Research, Cambridge, UK.

### Mice

BMDM were generated from the bone marrow of C57BL/6J wild‐type (WT) mice, Tpl2[K167R] knock‐in mice, caspase‐1 KO mice, IKKβ LysM‐Cre (flox/flox) mice and IKKβ CXCR3‐Cre (flox/flox) mice. The bone marrow was generously provided by the following scientists: Tpl2[K167R] knock‐in mice (Lopez‐Pelaez *et al*, 2011) (Susana Alemany, Biomedical Research Institute, Madrid, Spain), IKKβ LysM‐Cre (flox/flox) mice (Stefan Frantz, University of Wurzburg, Germany), IKKβ CXCR3‐Cre (flox/flox) mice (Manolis Pasparakis, University of Cologne, Germany) and caspase‐1 KO mice (Richard Flavell, Yale University, USA). WT C57BL/6J mice (Charles River, UK) were provided with free access to food (R&M3 pelleted irradiated diet) and water. Animals were kept in individually ventilated cages at 21°C, 45–65% relative humidity and a 12 h/12 h light/dark cycle under specific‐pathogen‐free conditions in accordance with UK and European Union regulations. Experiments on mice were approved by the University of Dundee ethical review board under a UK Home Office project license.

### Cells

Primary BMDM (Pauls *et al*, 2013) and primary human macrophages (Clark *et al*, 2012) were prepared as described. The mouse J774 A.1 macrophage cell line and iBMDM were maintained in DMEM supplemented with 100 units/ml penicillin and 100 μg/ml streptomycin, 1 mM sodium pyruvate, Glutamax (2 mM l‐alanyl‐l‐glutamine dipeptide and 0.085% NaCl), 10 mM HEPES buffer, 50 µM 2‐mercaptoethanol and 10% heat‐inactivated FCS (LabTech). All cell lines used in this study were negative for the presence of mycoplasma.

Outdated buffy coat from anonymous human blood donors were kindly provided by the Scottish National Blood Transfusion Services, Edinburgh. Peripheral blood mononuclear cells were isolated using Ficoll, and the monocytes were purified using anti‐CD14 magnetic beads (Miltenyi Biotec). Monocytes were differentiated into macrophages by incubation for 6 days in complete RPMI medium supplemented with 100 units/ml penicillin and 100 μg/ml streptomycin, 1 mM sodium pyruvate, Glutamax (2 mM l‐alanyl‐l‐glutamine dipeptide and 0.085% NaCl), 10 mM HEPES buffer, non‐essential amino acids, 50 µM 2‐mercaptoethanol and 10% heat‐inactivated FCS (LabTech) containing 100 ng/ml recombinant human M‐CSF (R&D).

### Immortalization of primary BMDMs

The conditioned supernatant collected from the CREJ2 cell line carrying the J2 retrovirus was used for immortalization. Bone marrow cells were first cultured for 7 days to generate primary BMDM (Pauls *et al*, 2013) from WT or IKKβ‐CXCR3‐Cre (flox/flox) mice. BMDM were then infected by incubating the cells in 30% L929 conditioned supernatant and 70% J2 retrovirus supernatant. One day later, the medium was removed and the BMDM were reinfected with fresh 30% L929 conditioned supernatant/70% J2 retrovirus containing supernatant. When the BMDM started to proliferate, forming cell clusters, the cells were passaged in fresh DMEM containing 25% L929 supernatant until the cells were confluent and then repassaged twice a week, decreasing the concentration of L929 supernatant by 5% each time. Immortalization was deemed to be complete when the iBMDM grew in the absence of L929 conditioned medium.

### Cell stimulation

Macrophages were incubated for 1 h with or without inhibitors and then co‐stimulated with inflammasome activators as specified in the figure legends. In some experiments, cells were stimulated for 4 h with LPS (100 ng/ml) to induce the transcriptional upregulation of NLRP3, pro‐IL‐1β and pro‐IL‐18 and then incubated for 1 h with inhibitors prior to activation of the NLRP3 inflammasome. To activate the AIM2 inflammasome macrophages were stimulated with poly(dA:dT) (2 μg/ml), which was introduced into the cells using Lipofectamine 2000 (Invitrogen) according to the manufacturer’s instructions.

### Cell lysis, preparation of cell extracts, Triton X‐100‐insoluble fraction and immunoblotting

Following stimulation with agonists, cells were rinsed with ice‐cold phosphate‐buffered saline (PBS), lysed in 1% (w/v) SDS containing the nuclease benzonase (50 units/ml) to hydrolyse DNA and then incubated for 5 min at 95°C. Alternatively, to prepare Triton X‐100‐soluble cell extracts, cells were washed twice in ice‐cold PBS, lysed in 50 mM Tris–HCl pH 7.5, 1 mM EDTA, 1 mM EGTA, 50 mM sodium fluoride, 5 mM sodium pyrophosphate, 1 mM sodium orthovanadate, 10 mM sodium 2‐glycerophosphate, 0.27 M sucrose, 1% (v/v) Triton X‐100, 1 mM dithiothreitol (DTT), 1 mM phenylmethylsulphonyl fluoride (PMSF), containing 1 mM benzamidine, and a protease inhibitor mixture (1 tablet per 50 ml buffer) (Roche; 11 873 580 001). After centrifugation for 15 min at 13,000 × *g* at 4°C, the supernatant, termed the Triton X‐100‐soluble fraction, was removed and its protein concentration quantified by the Bradford method. The Triton X‐100 soluble and insoluble fractions were incubated in 1% (w/v) SDS, and benzonase (50 units/ml) was added to the insoluble fraction. The samples (10 µg protein) were separated by SDS‐PAGE, transferred to PVDF membranes and immunoblotted with the antibodies specified in the figure legends.

### Cross‐linking of ASC

The Triton‐insoluble pellets were washed three times with ice‐cold PBS and then resuspended in 0.5 ml of PBS. The resuspended pellets were crosslinked by incubation for 45 min at 37°C with 2 mM disuccinimidyl suberate (Thermo Fisher Scientific), quenched using 50 mM Tris–HCl pH 7.5 and centrifuged for 15 min at 10,000 × *g*. The pellets were denatured in 1% (w/v) SDS, subjected to SDS–PAGE and analysed by immunoblotting.

### Precipitation of proteins from the cell culture medium

In order to detect the fragments of caspase‐1, IL‐1β and IL‐18 secreted into the cell culture medium, the proteins were concentrated using methanol chloroform precipitation (Jakobs *et al*, 2013) and redissolved in 1% (w/v) SDS to 20% the original volume of the culture medium.

### RNA‐mediated interference

J774 A.1 cells (3 × 10^6^) were transfected with 0.4 nmol siRNA using a Nucleofector II device (Lonza) with the Amaxa Cell Line Nucleofector V kit (Lonza), using program T020. The siRNAs for mouse IKKα, IKKβ and Cy3‐labelled negative control were purchased from Invitrogen (Life Technologies). After transfection for 24 h, the cells were replated in duplicate at 1 × 10^5^ cells/well. After a further 48 h, the cells were stimulated with inflammasome activators as detailed in the figure legends.

### Immunofluorescence and confocal microscopy

BMDM were seeded onto glass coverslips placed inside 24‐well (4 × 10^5^ cells per well) tissue culture plates and then treated with inhibitors and stimulated with ligands as specified in the figure legends. For PLA and immunostaining, cells were fixed for 10 min at ambient temperature with 4% (w/v) paraformaldehyde in PBS. Excess paraformaldehyde was quenched by incubating the fixed cells for 15 min at 21°C in 0.1 M glycine, followed by permeabilization with 0.5% (w/v) saponin in PBS. Saponin was included at all subsequent steps. For immunofluorescence, non‐specific signals were blocked for 60 min with 10% (v/v) normal donkey serum and the fixed cells incubated overnight at 4°C with rabbit polyclonal TGN38 primary antibody (1:200), followed by incubation with Alexa Fluor 594 (red) donkey anti‐rabbit (A32754 from Invitrogen) or Alexa Fluor 488 (green) donkey anti‐rabbit (A21206 from Invitrogen) secondary antibodies (1:300) to visualize the primary antibody. The nuclei were stained with DAPI, and coverslips were mounted onto glass slides using Vectashield Antifade Mounting Medium (H‐1000 from Vector Laboratories). Images were acquired on a Zeiss LSM 710 or LSM 880 confocal microscope using a x63 Plan‐Apochromat objective (NA 1.4). Each fluorescent channel was collected independently to prevent spectral bleed‐through, and the optical section thickness was set to 0.8 µm for all channels. For every treatment within an experiment 10 images were taken from the coverslip, and areas were selected by uniform random sampling. The fields of view and plane of focus were chosen using the DAPI channel without viewing the TGN38 signal. Images presented in the Figures were chosen as representative of the 10 images collected.

### Proximity ligation assays (PLA)

These assays employed two different antibodies, one recognizing NLRP3 and the other TGN38, a positive PLA signal only being seen when NLRP3 and TGN38 come into close proximity. PLA was performed using a Duolink Detection Kit with Texas Red signal amplification (Merck Sigma‐Aldrich). The PLA assay was carried out as described above for immunofluorescence detection, up to the stage of permeabilization. Primary antibody and probe incubation, ligation and amplification reactions were then carried out following the protocol provided with the kit. Cells were examined with a confocal microscope (objective × 63 Plan‐Apochromat objective (NA 1.4), Zeiss LSM 710 or 880) and Texas Red dots identified as objects with an intensity greater than 6 standard deviations from the mean image intensity in Velocity (Quorum Technologies Inc, Ontario). Nuclei (DAPI) were identified using the automatic method (Otsu’s method) (Otsu, 1979) using an offset of −42. Holes were filled in identified nuclei and all DAPI objects with an area < 5 mm^2^ were excluded. 10 images were collected for each treatment, each image containing 20–60 cells. The summed pixel intensity for the Texas Red dots in each image (sum of the sums) was divided by the number of nuclei to give the summed intensity per cell. For every treatment within an experiment 10 images were taken from the coverslip, areas were selected by uniform random sampling (random start point with uniform distances between fields of view). The fields of view and plane of focus were chosen using the DAPI channel without viewing the PLA signal (both by manual, eyepiece viewing and confocal imaging). All 10 images were quantified as described above.

### Statistical analysis

Quantitative data are represented as mean ± SEM. Shapiro–Wilk test was employed to determine if the data have normal distribution. The statistical significance of differences between experimental groups was calculated with GraphPad Prism Software using two‐way ANOVA (analysis of variance) followed by Sidak’s multiple comparison or one‐way ANOVA followed by Kruskal–Wallis test with Dunn’s multiple comparison. Differences in means were considered significant if *P* < 0.05.

## Author contributions

SKN: Conceptualization, formal analysis of data, investigation, validation, methodology, visualization, funding acquisition, project administration, supervision, writing‐original draft, review and editing. ARP: acquisition and formal analysis of microscopy data, investigation, methodology, visualization and writing microscopy methodology. CF‐V: investigation. PC: conceptualization, supervision, funding acquisition, project management, writing‐original draft, review and editing.

## Conflict of interest

Sambit Kumar Nanda is presently an employee of AstraZeneca and has stock ownership and/or stock options or interests in the company. The other authors declare that they have no conflict of interest.

## Supporting information



Expanded View Figures PDF

Source Data for Expanded View

Source Data for Figure 1

Source Data for Figure 2

Source Data for Figure 3

Source Data for Figure 4

Source Data for Figure 5

## Data Availability

No primary data sets have been generated and therefore not deposited.

## Footnotes

^‡^
Correction added on 5 October 2021, after first online publication: “GSDMD(NT) was generated” has been corrected to “GSDMD was cleaved”.

^§^
Correction added on 5 October 2021, after first online publication: “GSDMD(NT)” has been corrected to “GSDMD(CL)” in panels A‐C. Additionally “cleaved (NT) gasdermin D (GSDMD)” has been corrected to “cleaved (CL) gasdermin D (GSDMD)” in the legend of panel A,B.

^¶^
Correction added on 5 October 2021, after first online publication: “(GSDMD(NT))” has been corrected to “(GSDMD(CL))”.
