# Supplementary material for: IKKβ is required for the formation of the NLRP3 inflammasome
Source: EMBO Rep. 2021 Aug 17;22(10):e50743. doi: 10.15252/embr.202050743 (PMC8490994; doi:10.15252/embr.202050743)
Supplement: Supplementary file 1 — Expanded View Figures PDF [file EMBR-22-e50743-s006.pdf]

Expanded View Figures

Figure EV1. Inhibition of *de novo* transcription or protein synthesis does not affect the rapid activation of the NLRP3 inflammasome.

A, B WT BMDM were incubated for 30 min without (−) or with (+) 5 μg/ml actinomycin D (ActD) (A) or 10 μg/ml cycloheximide (Chx) (B). The cells were then co-stimulated for 30 min with (+) or without (−) 100 ng/ml LPS and 4 mM ATP or for 1 h with 100 ng/ml LPS and 5 μM nigericin. Cell lysates (10 μg protein) were subjected to SDS–PAGE and immunoblotted with the antibodies indicated. Similar results were obtained in three independent experiments.

C WT BMDM were incubated for 30 min without (−) or with (+) 5 μg/ml actinomycin D (ActD) or 10 μg/ml cycloheximide (Chx) and then stimulated for 4 h with (+) 100 ng/ml LPS or left unstimulated (−). Cell extract protein (10 μg) was subjected to SDS–PAGE and immunoblotted with anti-NLRP3. GAPDH was used as a loading control. Similar results were obtained in two independent experiments.

D As in C, except that the cells were stimulated for the times indicated (hours) and immunoblotting was performed with anti-DUSP1 (dual specificity phosphatase 1). Similar results were obtained in two independent experiments.

E, F As in A, B, except that immunoblotting was performed with anti-NLRP3 and anti-GAPDH. Similar results were obtained in two independent experiments.

Source data are available online for this figure.

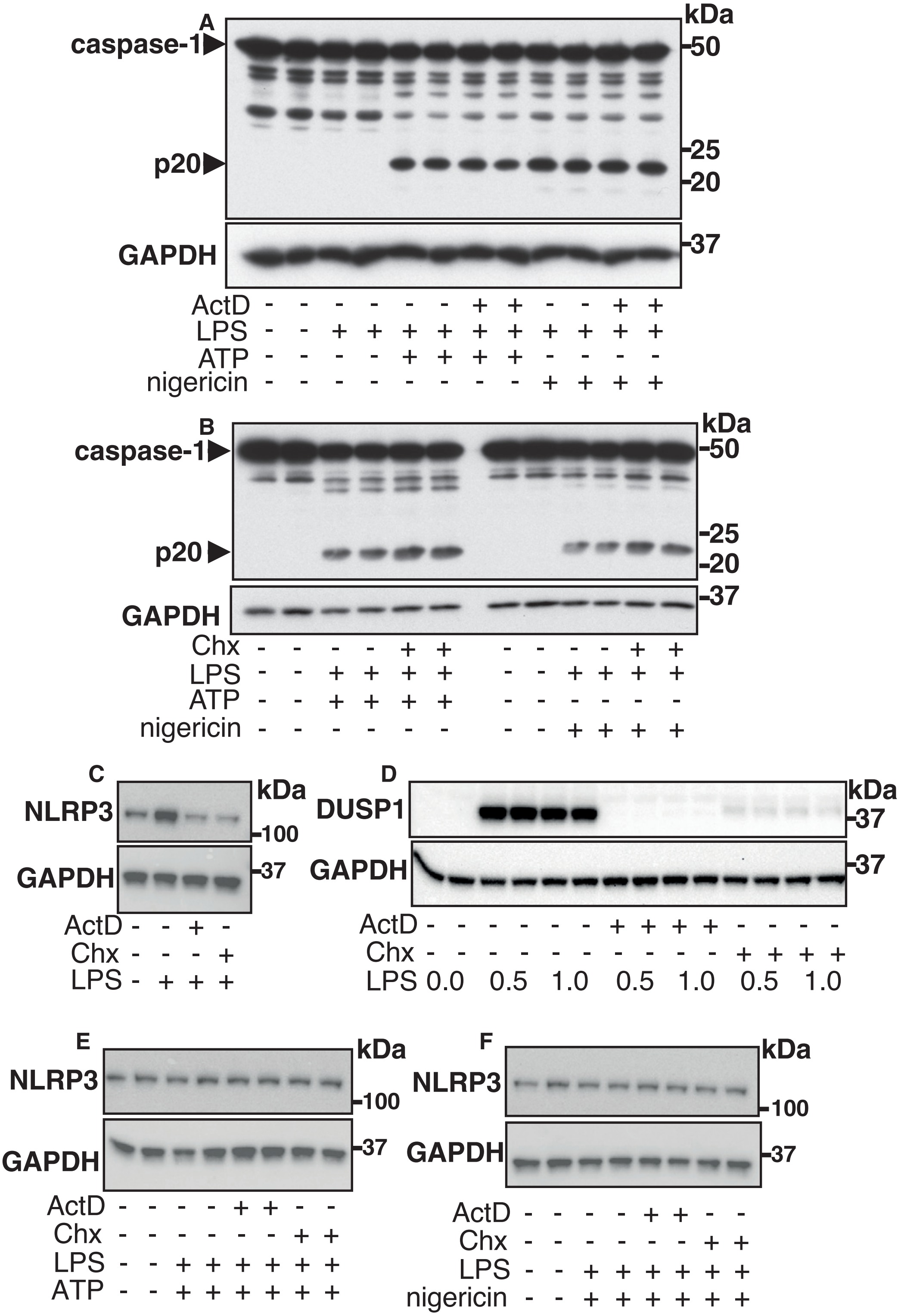

Figure EV1.

 © 2021 The Authors

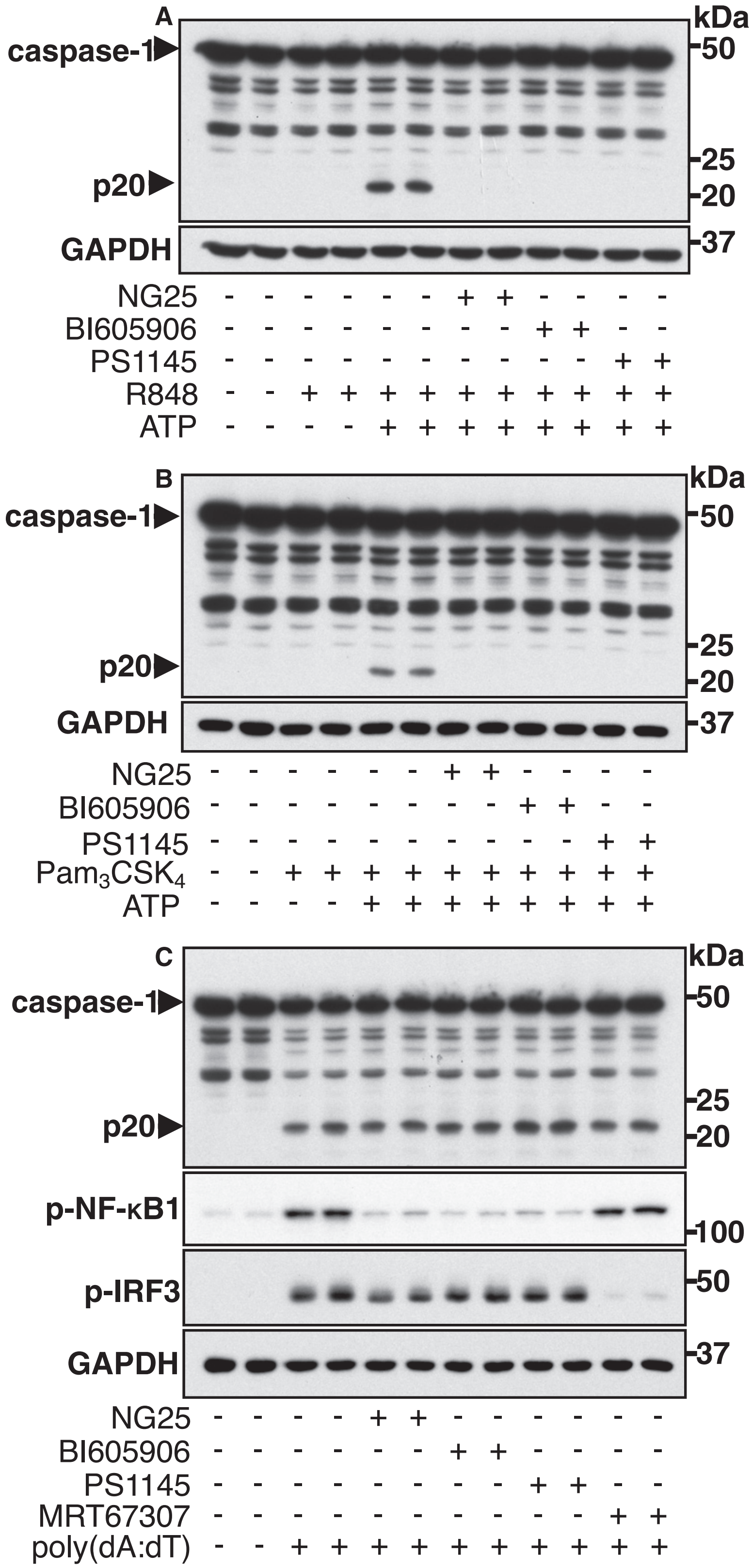

Figure EV2. TAK1 or IKKβ inhibitors block activation of the NLRP3 inflammasome but not the AIM2 inflammasome.

A BMDM were incubated for 1 h without (−) or with (+) 2 μM NG25, 5 μM BI605906 or 10 μM PS1145 and then stimulated for 30 min without (−) or with (+) 1 μg/ml R848 and 4 mM ATP. Cell lysates were subjected to SDS–PAGE and immunoblotted with the antibodies indicated. Similar results were obtained in two independent experiments.

B As in A, except that 1 μg/ml Pam_3CSK_4 was used instead of R848.

C As in A except that MRT67307 (2 μM), an inhibitor of the IKK-related kinases TBK1 and IKKε was also included and the cells were stimulated by transfection for 2 h with 2 μg/ml poly(dA:dT) to activate the AIM2 inflammasome (+) or mock transfected (−). Similar results were obtained in two independent experiments.

Source data are available online for this figure.

Figure EV3. The inhibition of protein kinases activated by IKKβ does not affect the activation of caspase-1 induced by the formation of the NLRP3 inflammasome.

A, B WT BMDM were incubated for 1 h without (−) or with (+) 2 μM of the TBK1/IKKε inhibitor MRT67307 and then stimulated for 30 min without (−) or with (+) 100 ng/ml LPS and/or 4 mM ATP (A) or 5 μM nigericin (B). Cell lysates (10 μg protein) were subjected to SDS–PAGE and immunoblotted with the antibodies indicated. Similar results were obtained in three independent experiments.

C, D As in A, B, except that BMDM from knock-in mice expressing the catalytically inactive Tpl2[K167R] mutant or WT control mice were used. Similar results were obtained in two independent experiments.

E, F As in A, B except that the cells were incubated without (−) or with (+) 1 μM GSK2578215A (GSK), an inhibitor of LRRK2. Similar results were obtained in three independent experiments.

Source data are available online for this figure.

 © 2021 The Authors

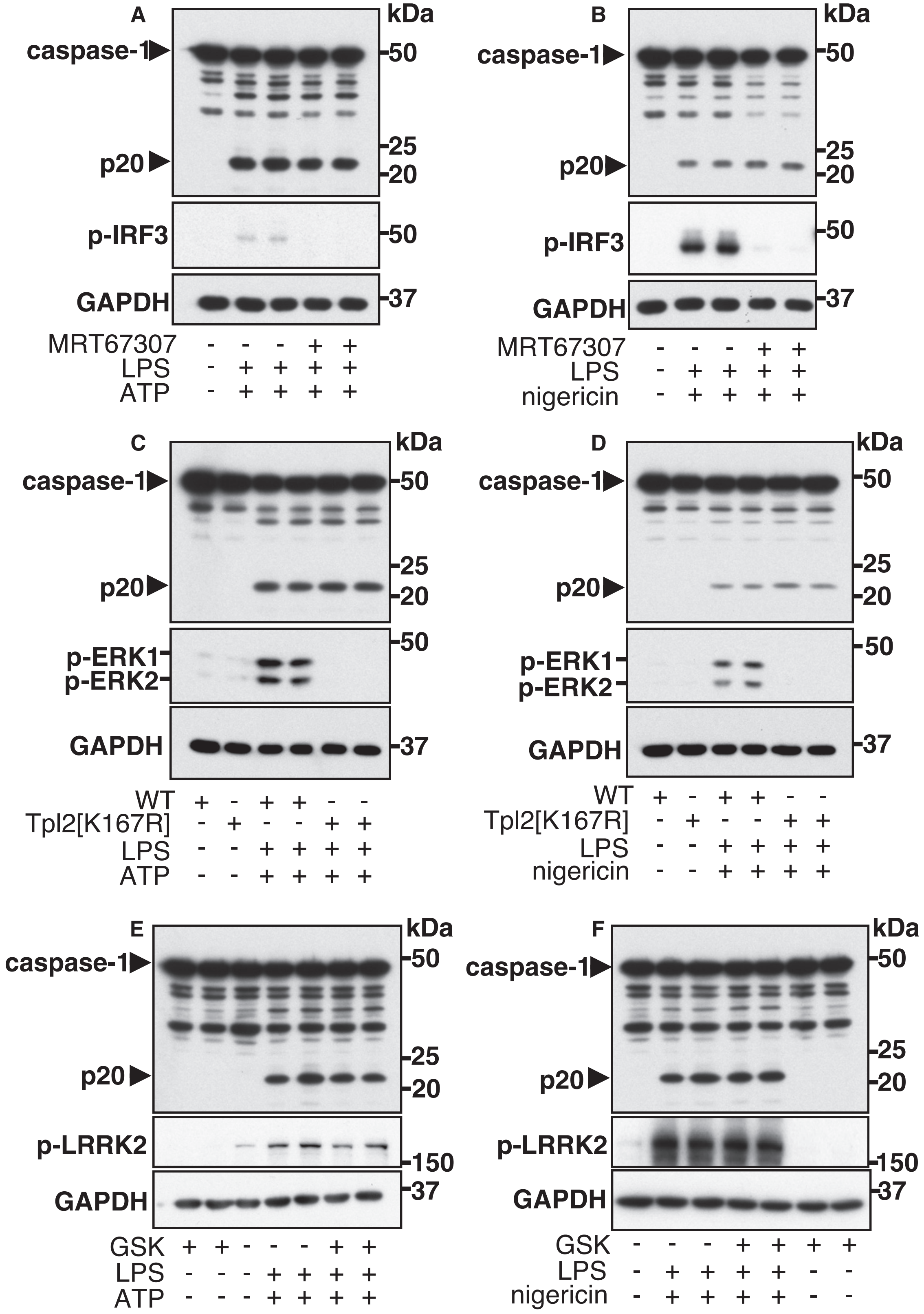
A
kDa
caspase-1
p20
p-IRF3
GAPDH
50
25
20
50
37
MRT67307 - - - + +
LPS - + + + +
ATP - + + + +
B
kDa
caspase-1
p20
p-IRF3
GAPDH
MRT67307 - - - + +
LPS - + + + +
nigericin - + + + +
C
p-ERK1
p-ERK2
WT + - + + - -
Tpl2[K167R] - + - - + +
LPS - - + + + +
ATP - - + + + +
D
WT + - + + - -
Tpl2[K167R] - + - - + +
LPS - - + + + +
nigericin - - + + + +
E
p-LRRK2
150
GSK + + - - - + +
LPS - - - + + + +
ATP - - - + + + +
F
GSK - - - + + + +
LPS - + + + + - -
nigericin - + + + + - -

Figure EV3.

Figure EV4. IKKβ stimulates the formation of the NLRP3 inflammasome and its activation is independent of caspase8.

A WT BMDM were incubated for 1 h without (−) or with (+) 5 μM BI605906, 5 μM TPCA-1 or 1 μM MCC950. The cells were then stimulated for 1 h with (+) 100 ng/ml LPS and/or 5 μM nigericin, or left unstimulated (−). After cell lysis in the presence of 1% (v/v) Triton X-100, the Triton X-100-insoluble fractions were prepared as in Fig 4, denatured in SDS, subjected to SDS–PAGE and immunoblotted with anti-ASC or with an antibody against the U2 small nuclear RNA auxiliary factor 1 (U2AF1) as a loading control.

B As in A, except that the Triton X-100-insoluble fraction was first crosslinked by incubation for 45 min at 37°C with 2.0 mM DSS. (A, B) Similar results were obtained in two independent experiments.

C WT BMDM were incubated for 1 h with (+) or without (−) the IKKβ inhibitors BI605906 (5 μM), TPCA-1 (5 μM) or PS1145 (10 μM), the TAK1 inhibitor NG25 (2 μM) or the NLRP3 inflammasome inhibitor MCC950 (1 μM). The cells were then stimulated with LPS (100 ng/ml) and/or 5 μM nigericin (+) or left unstimulated (-). Cells were stimulated with TNF (10 ng/ml) and cycloheximide (Chx) (10 μg/ml), which was used as a positive control for a signal generating cleaved (CL) caspase-8. Cells were lysed, cell extracts (10 μg protein) were subjected to SDS–PAGE and immunoblotted with antibodies recognizing caspase-8(CL) (the cleavage product of caspase-8) and GAPDH.

D As in C, except that the samples were immunoblotted for full length caspase-1 (caspase-1), the p20 fragment of caspase-1 (p20) and GAPDH. Similar results were obtained in two independent experiments.

E WT BMDM were stimulated for 4 h without (Panel 1, control) or with (Panels 2–5) 100 ng/ml LPS and then incubated for 1 h without (Panels 1–3) or with 5 μM TPCA-1(Panel 4) or 5 μM BI605906 (Panel 5) and then stimulated for 60 min with 5 μM nigericin (Panels 3–5) or without nigericin (Panels 1 and 2). The cells were fixed and processed for immunofluorescence using a rabbit polyclonal antibody against TGN38, which was visualized using a secondary antibody (red). Nuclei were counterstained with DAPI (blue). Images were acquired by sequential laser scanning on the confocal microscope. Similar results were obtained in three independent experiments, and representative images are shown.

Data information: In all panels, scale bar = 50 μm.
Source data are available online for this figure.

 © 2021 The Authors

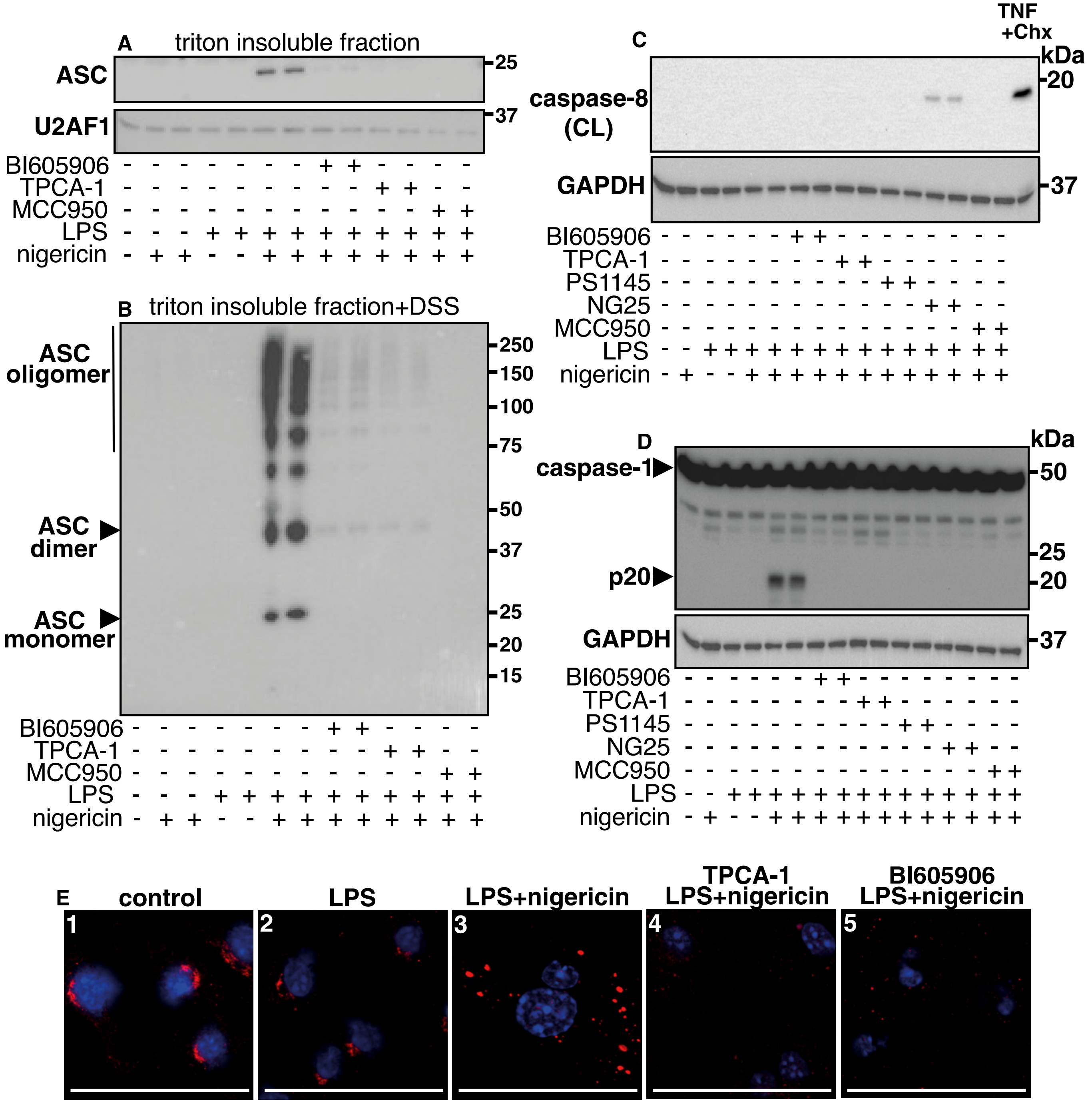
A triton insoluble fraction
ASC
U2AF1
BI605906
TPCA-1
MCC950
LPS
nigericin
B triton insoluble fraction+DSS
ASC oligomer
ASC dimer
ASC monomer
C
TNF +Chx
kDa
caspase-8 (CL)
GAPDH
PS1145
NG25
D
caspase-1
p20
E
control
LPS
LPS+nigericin
TPCA-1 LPS+nigericin
BI605906 LPS+nigericin

Figure EV4.

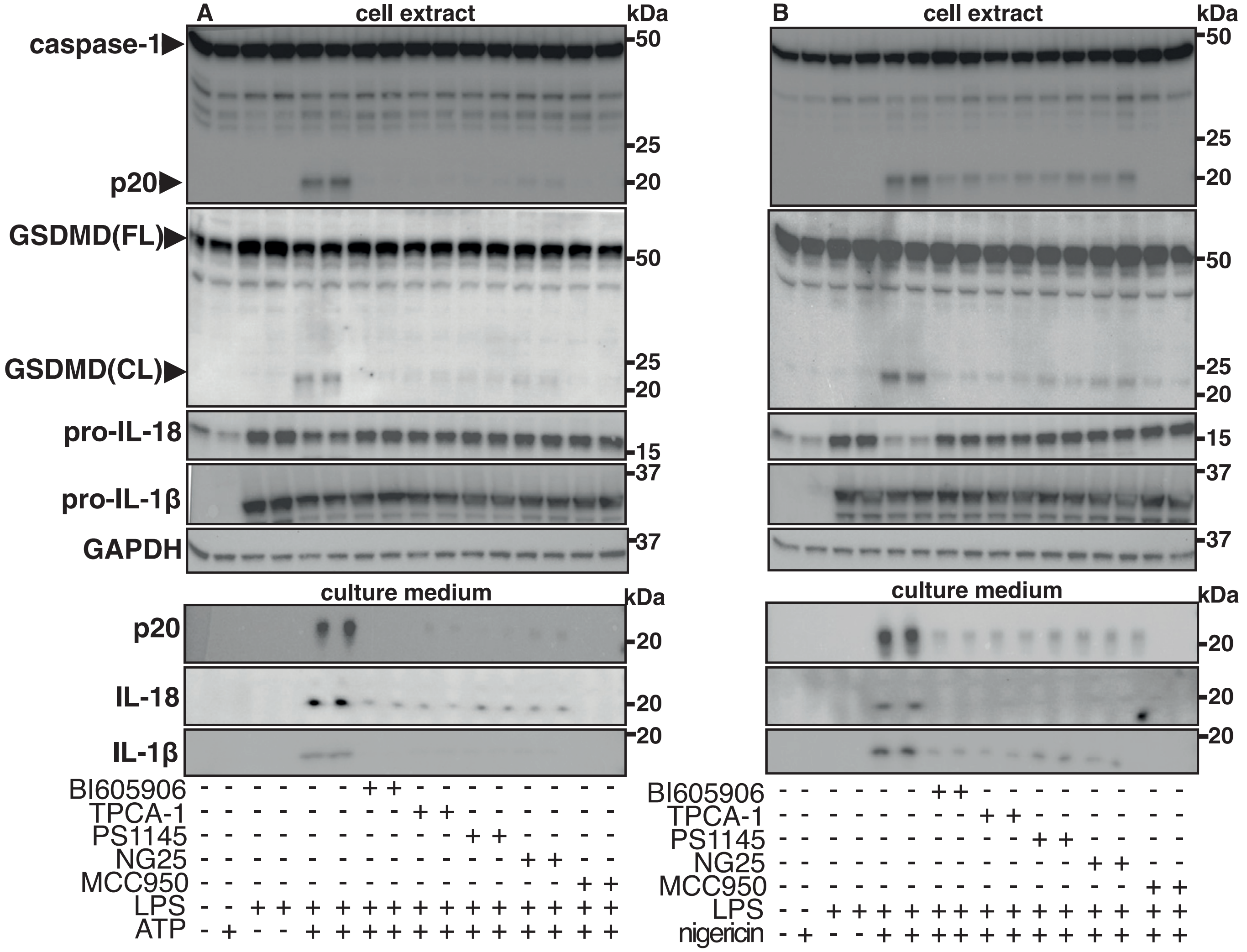

Figure EV5. Inhibition of IKKβ and TAK1 prevents inflammasome activation after transcriptional upregulation of NLRP3.

A WT BMDM were either incubated for 4 h (+) with LPS (100 ng/ml) or left untreated (−) and then incubated for 1 h without (−) or with (+) BI605906 (5 μM) or TPCA-1 (5 μM) or PS1145 (10 μM) or NG25 (2 μM) or MCC950 (1 μM). The cells were then stimulated for 30 min with (+) 4 mM ATP, the culture medium removed, the cells lysed and the cell lysates (10 μg protein) denatured in SDS. The protein in the cell culture medium was precipitated (see Materials and Methods) and also dissolved in SDS. The cell extracts and samples from the cell culture medium were subjected to SDS–PAGE, transferred to PVDF membranes and immunoblotted with antibodies recognizing full length (FL) or cleaved (CL)[#] gasdermin D (GSDMD), the p20 fragment of caspase-1, IL-18 and IL-1β.

B As in A, except that the cells were stimulated with nigericin (5 μM, 1 h) instead of ATP. Similar results were obtained in two independent experiments.

Source data are available online for this figure.

[#]Correction added on 5 October 2021, after first online publication: "GSDMD(NT)" has been corrected to "GSDMD(CL)" in panel A. Additionally "cleaved (NT) gasdermin D (GSDMD)" has been corrected to "cleaved (CL) gasdermin D (GSDMD)" in the legend of panel A.

 © 2021 The Authors
