# Supplementary material for: IKKβ is required for the formation of the NLRP3 inflammasome
Source: EMBO Rep. 2021 Aug 17;22(10):e50743. doi: 10.15252/embr.202050743 (PMC8490994; doi:10.15252/embr.202050743)

Figure EV5

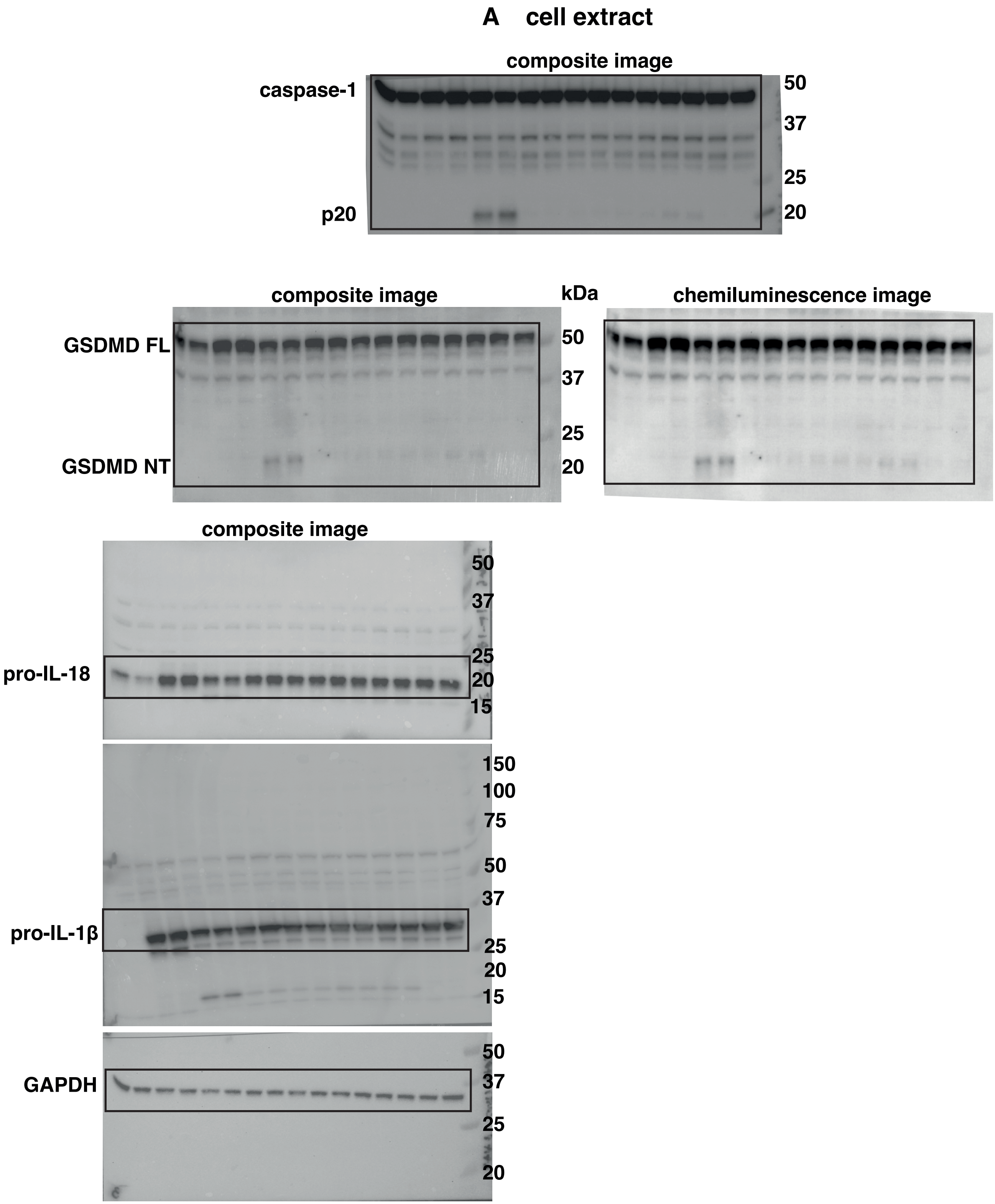
A cell extract
composite image
caspase-1
p20
50
37
25
20
composite image
kDa
chemiluminescence image
GSDMD FL
GSDMD NT
50
37
25
20
composite image
pro-IL-18
50
37
25
20
15
pro-IL-1β
150
100
75
50
37
25
20
15
GAPDH
50
37
25
20

Figure EV5

A culture medium

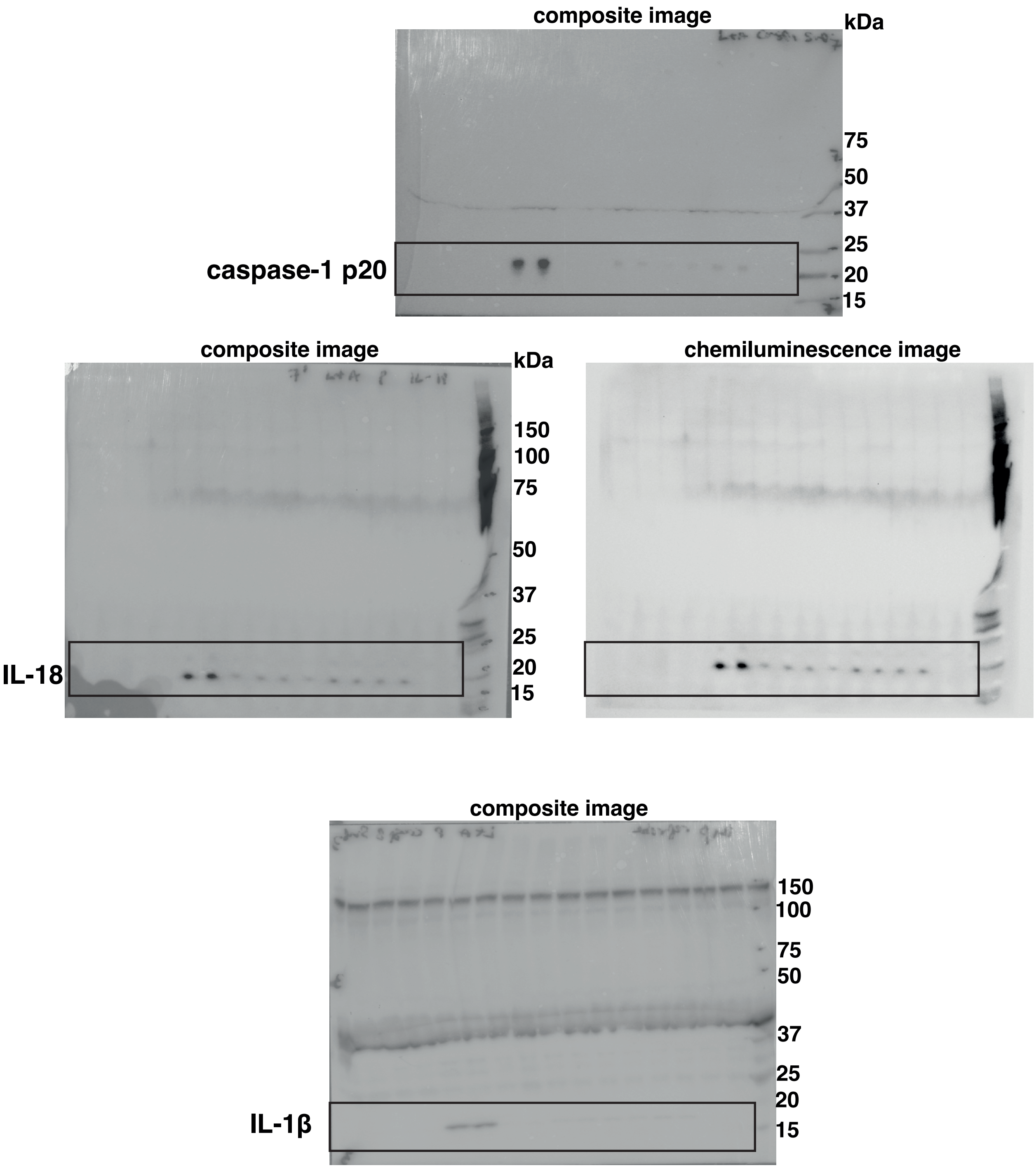

Figure EV5

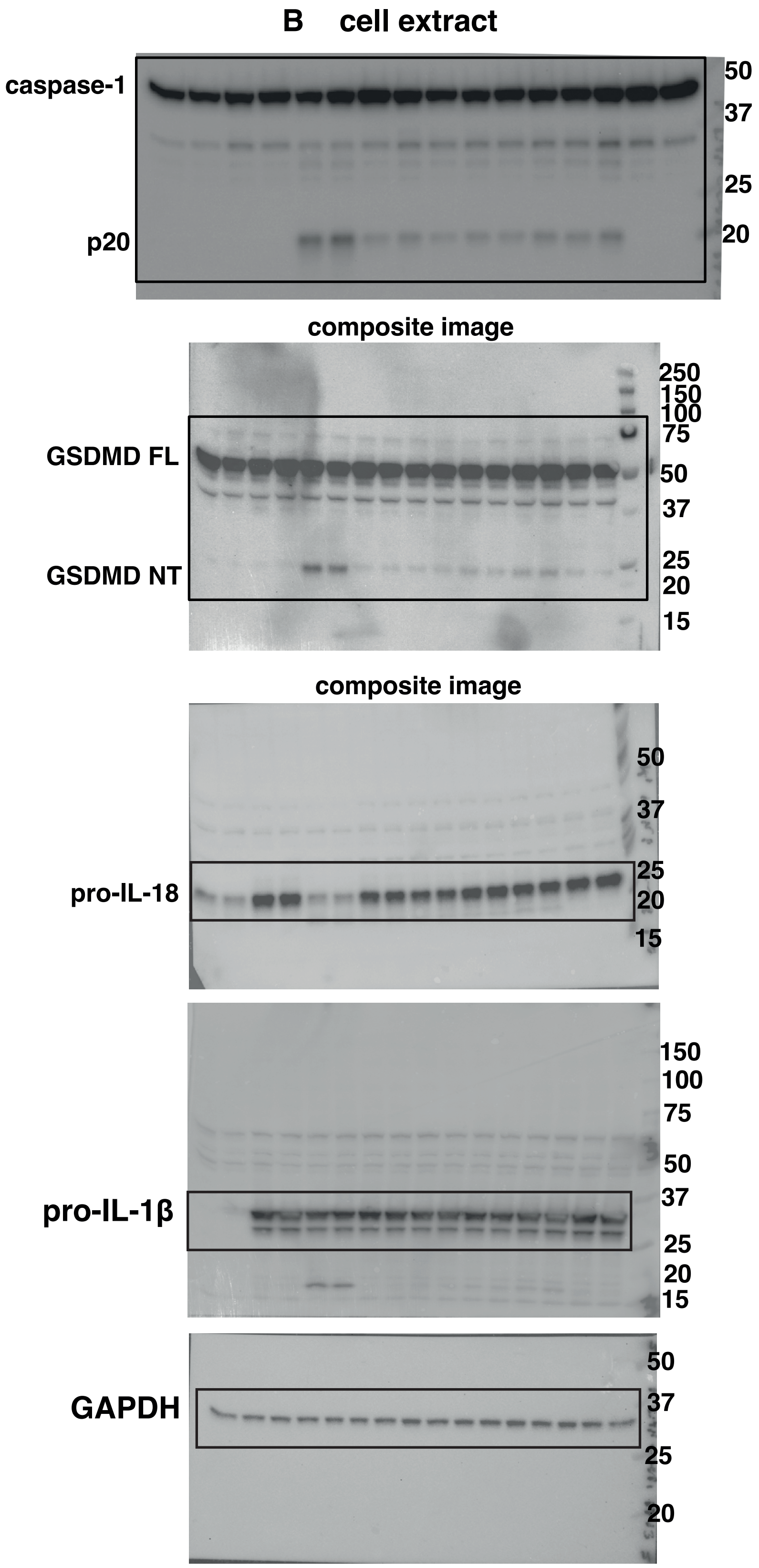
B
cell extract
caspase-1
p20
50
37
25
20
composite image
GSDMD FL
GSDMD NT
250
150
100
75
50
37
25
20
15
composite image
pro-IL-18
50
37
25
20
15
pro-IL-1β
150
100
75
50
37
25
20
15
GAPDH
50
37
25
20

Figure EV5

B culture medium

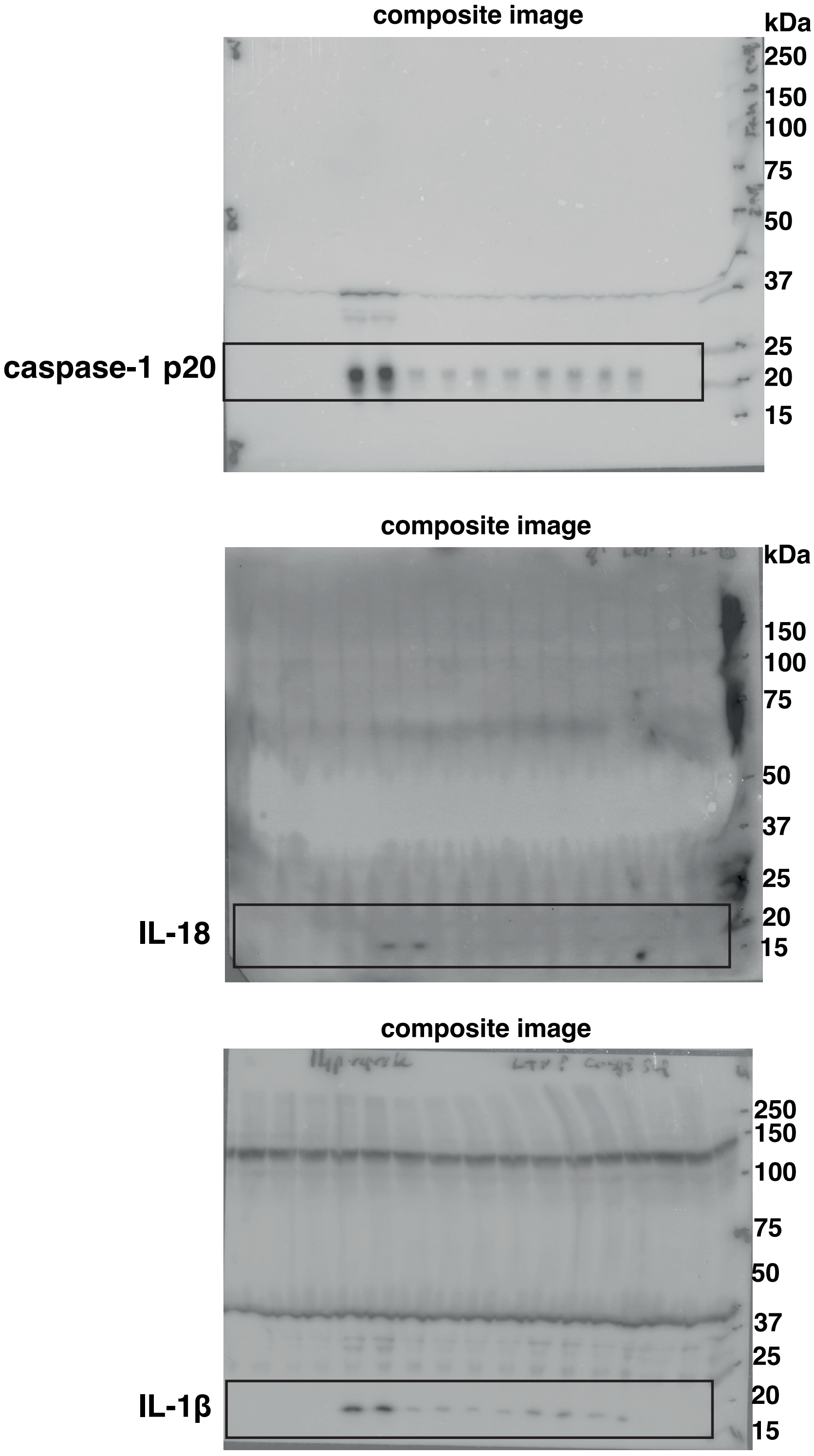

Supplement: Supplementary file 2 — Source Data for Expanded View [file EMBR-22-e50743-s007.zip › embr202050743-sup-0011-SDataFigEV5.pdf]
