# Supplementary figures and images for: IKKβ is required for the formation of the NLRP3 inflammasome
Source: EMBO Rep. 2021 Aug 17;22(10):e50743. doi: 10.15252/embr.202050743 (PMC8490994; doi:10.15252/embr.202050743)

**Figure EV1**

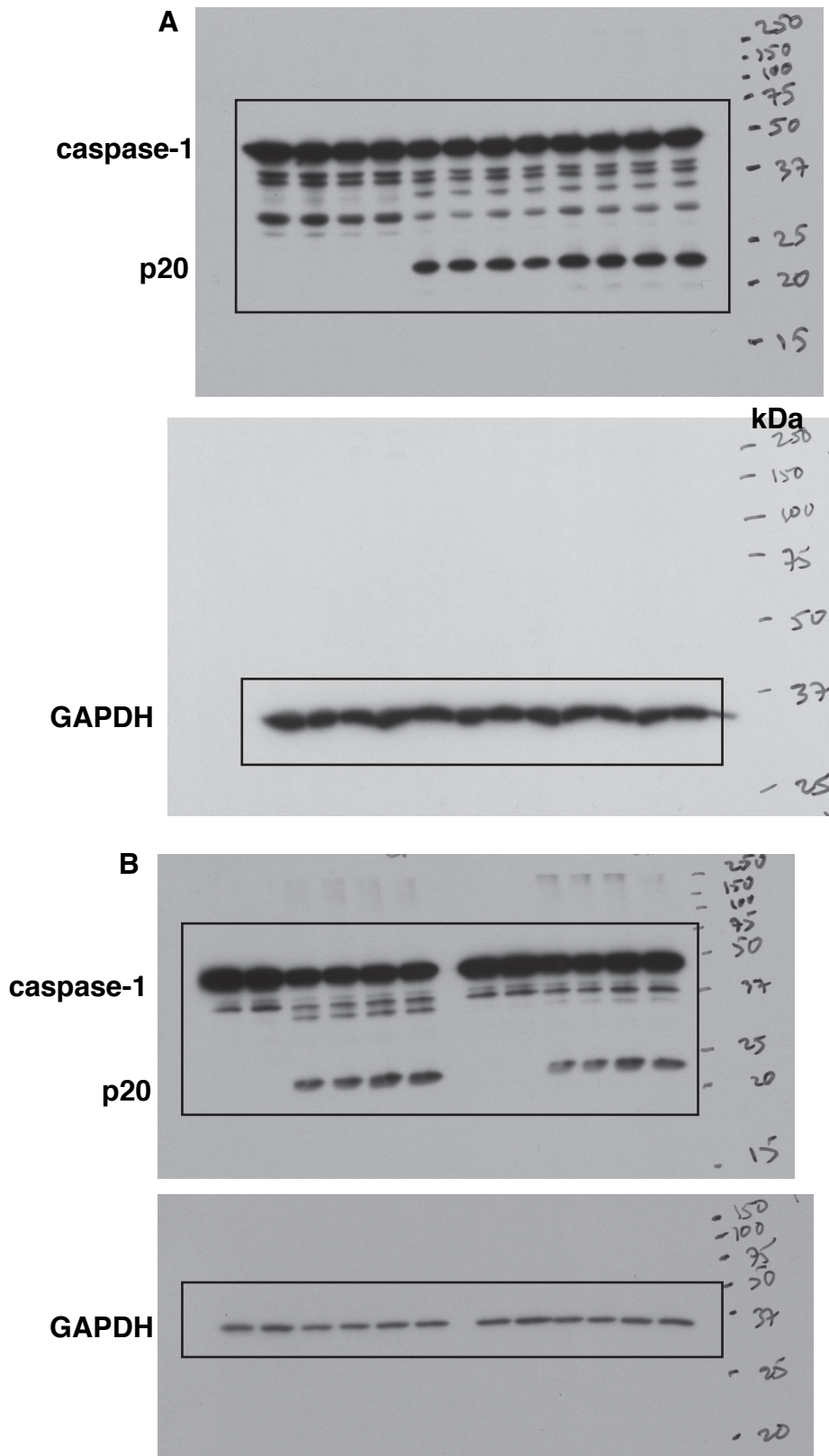

**Figure EV1**

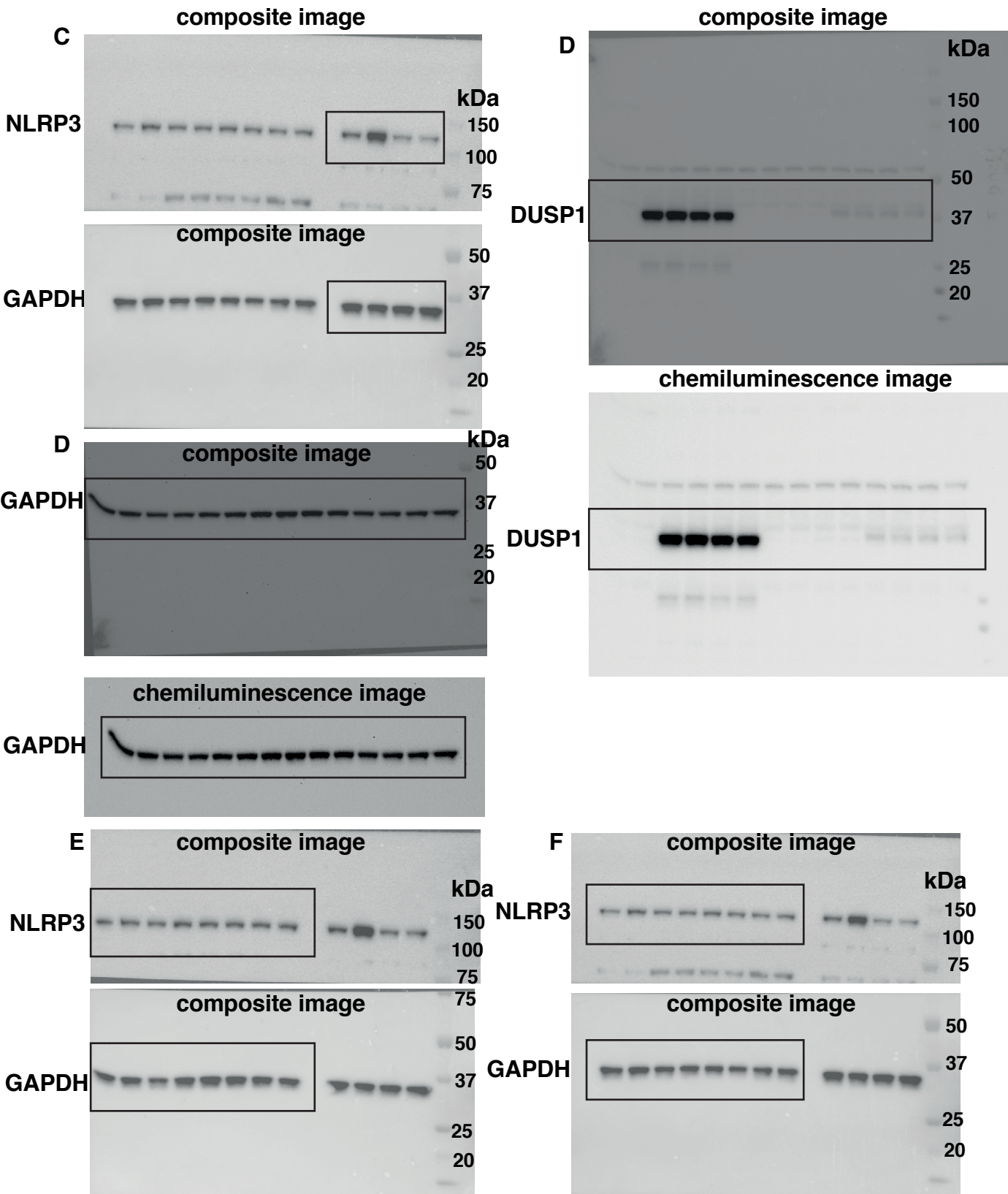

Supplement: Supplementary file 2 — Source Data for Expanded View [file EMBR-22-e50743-s007.zip › embr202050743-sup-0007-SDataFigEV1.pdf]

# Figure EV2

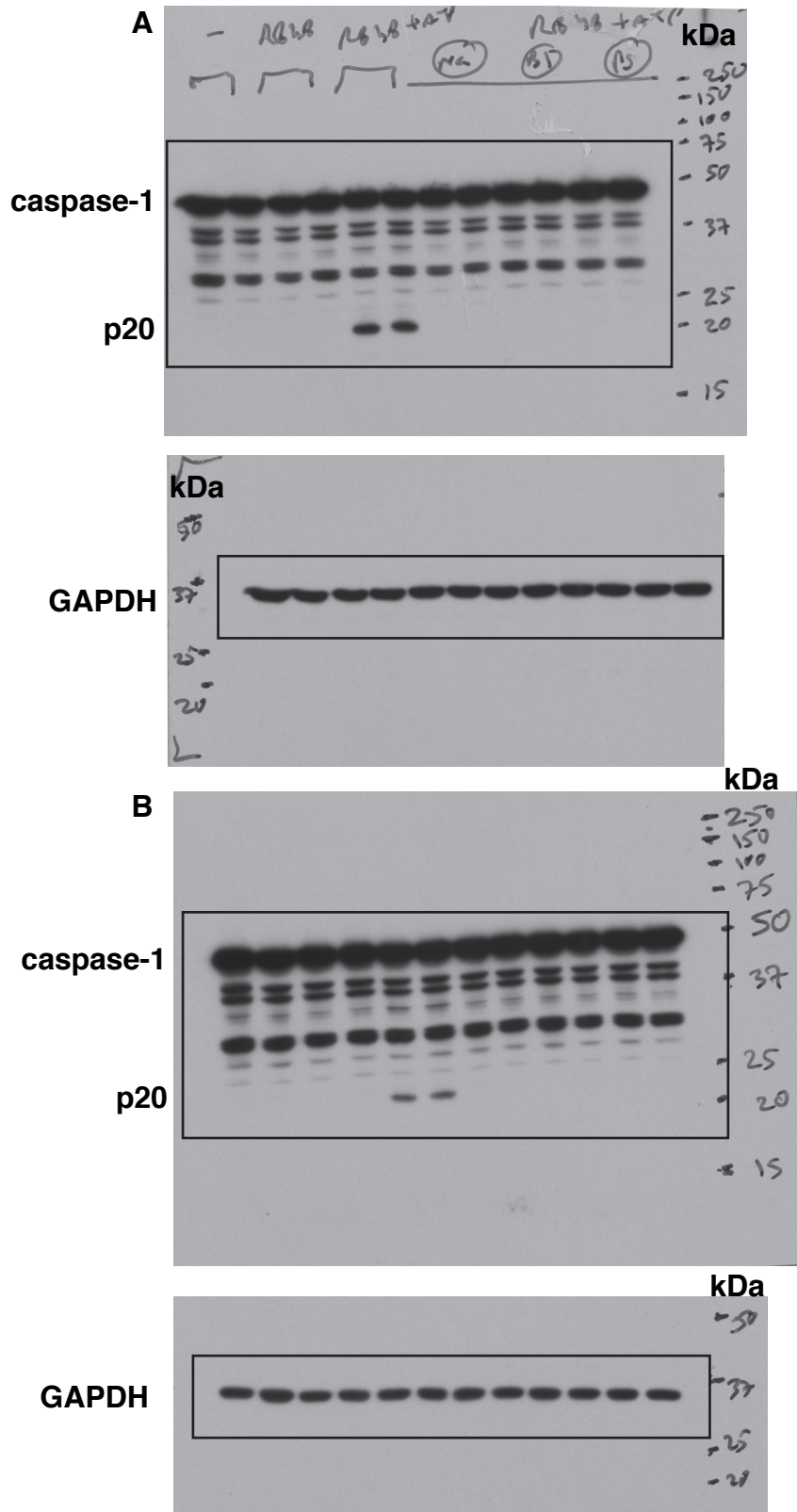

# Figure EV2

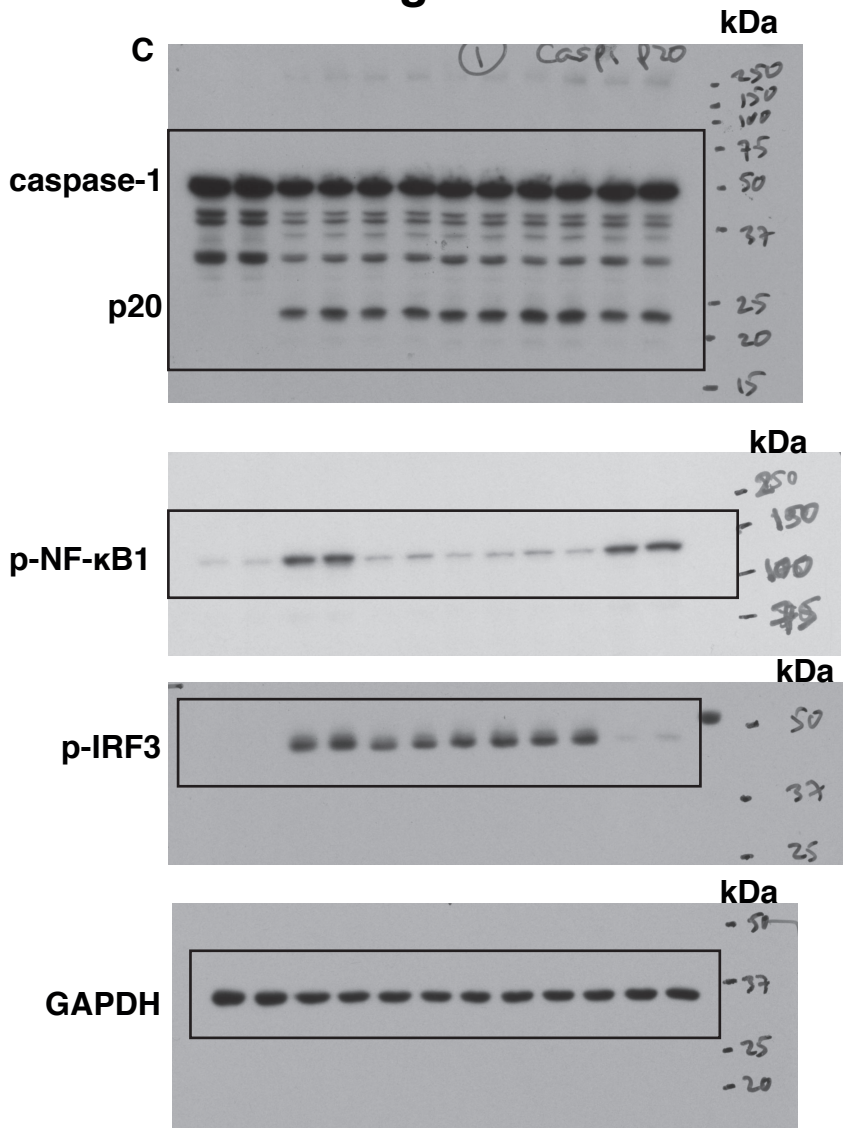

Supplement: Supplementary file 2 — Source Data for Expanded View [file EMBR-22-e50743-s007.zip › embr202050743-sup-0008-SDataFigEV2.pdf]

# Figure EV3

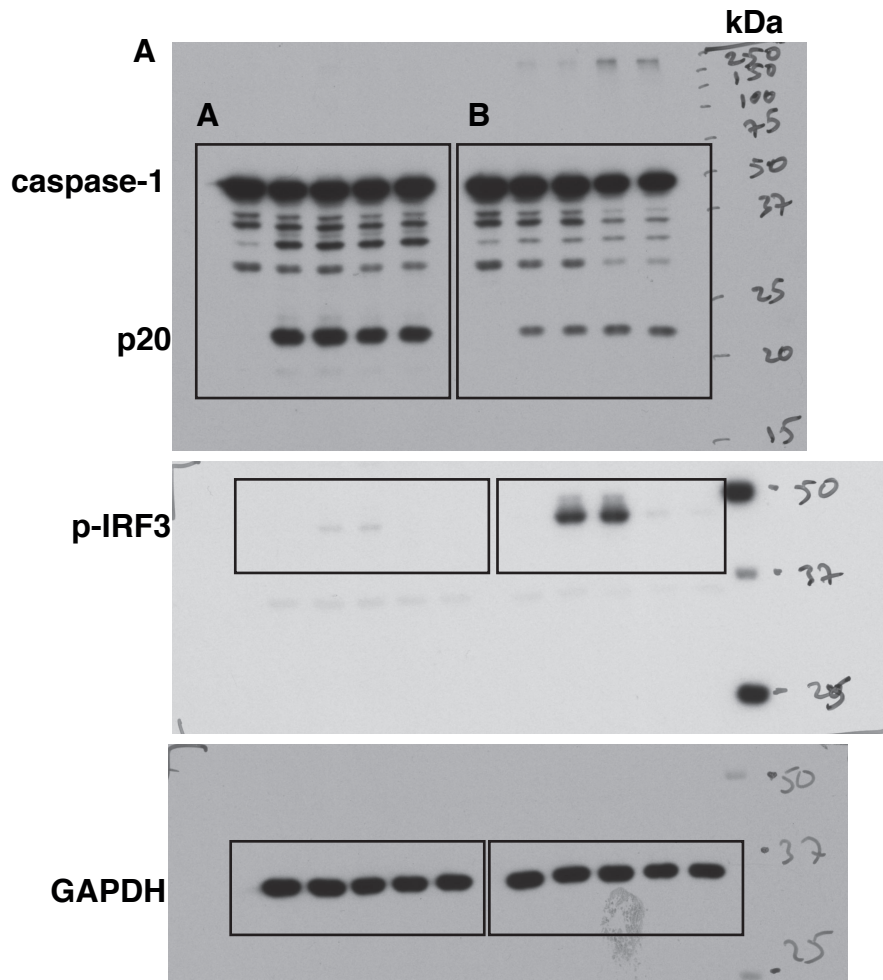

## Figure EV3

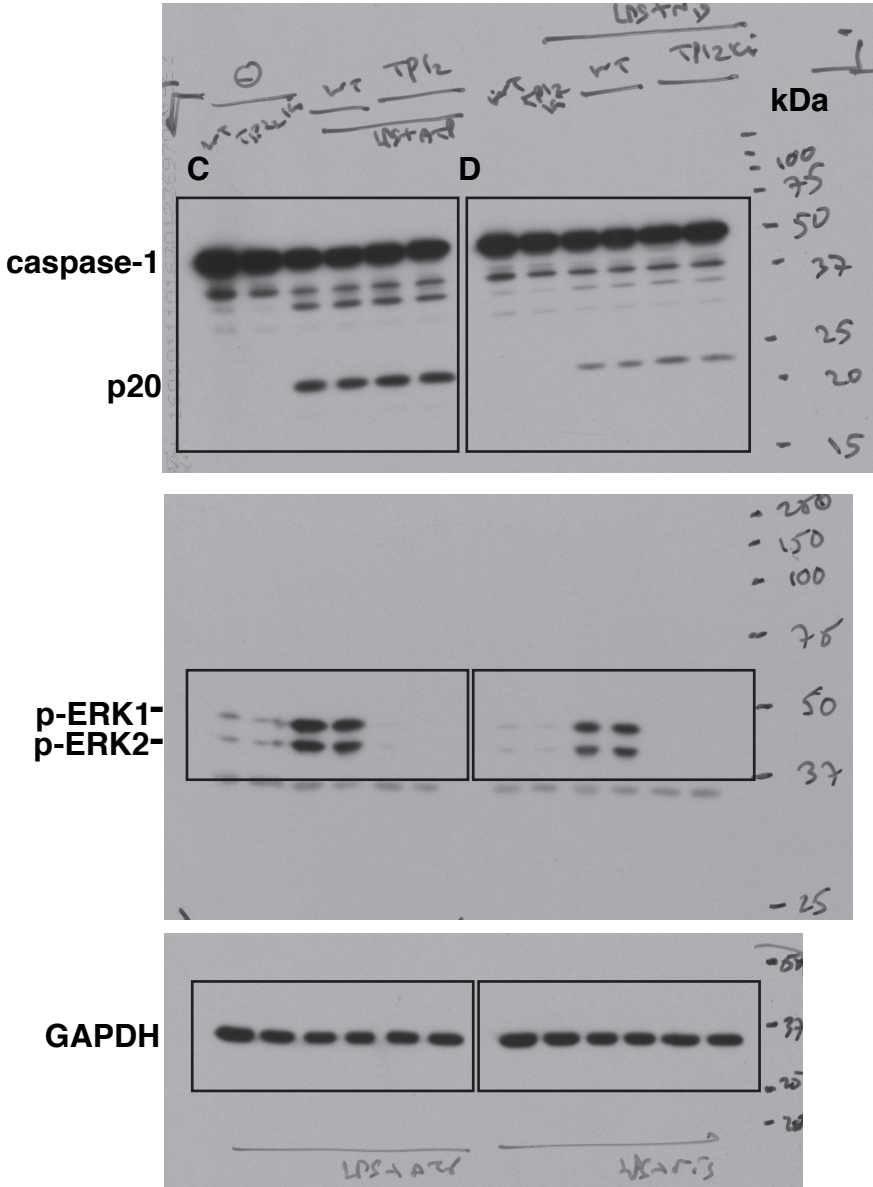

# Figure EV3

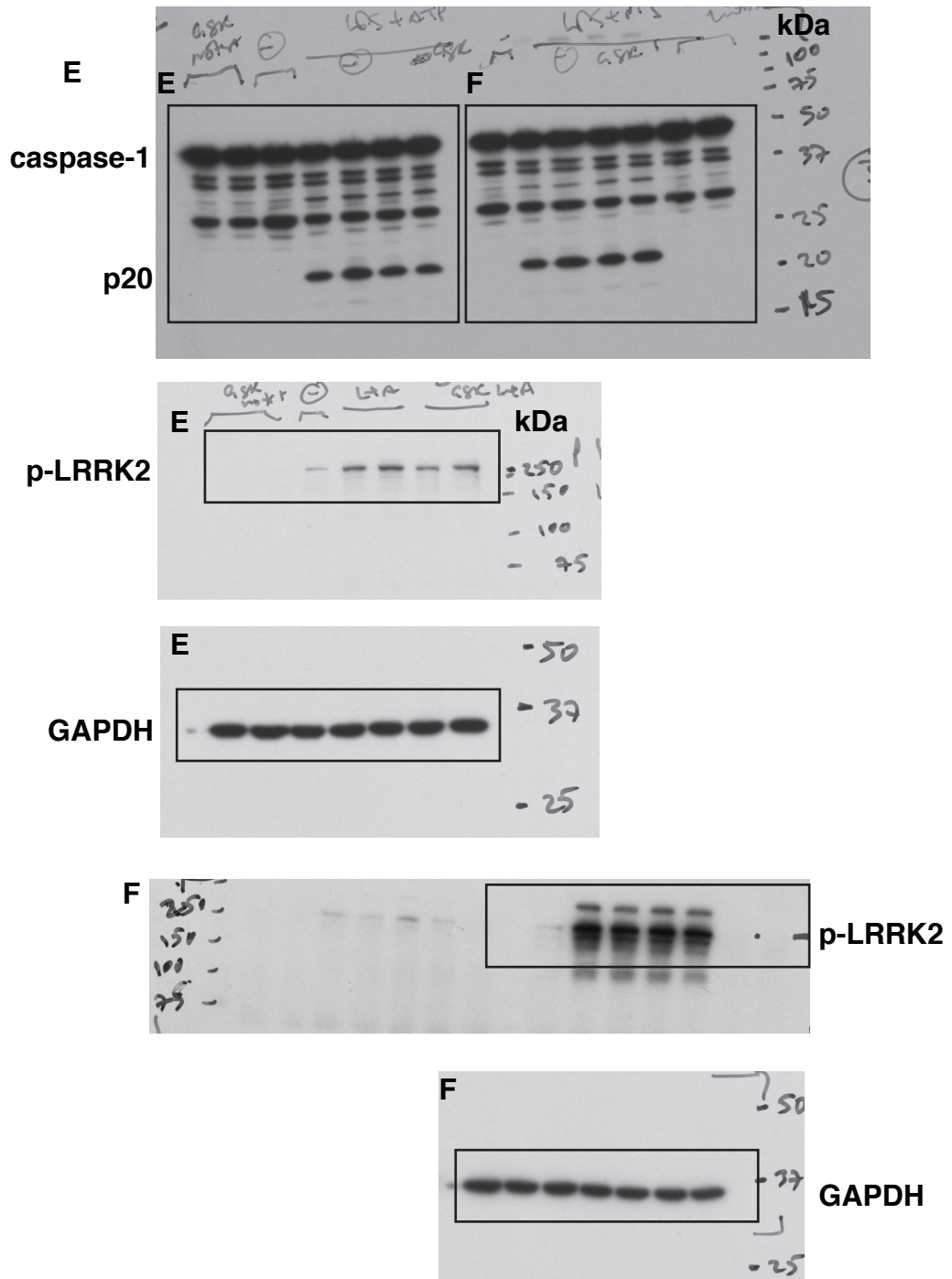

Supplement: Supplementary file 2 — Source Data for Expanded View [file EMBR-22-e50743-s007.zip › embr202050743-sup-0009-SDataFigEV3.pdf]

# Figure EV4

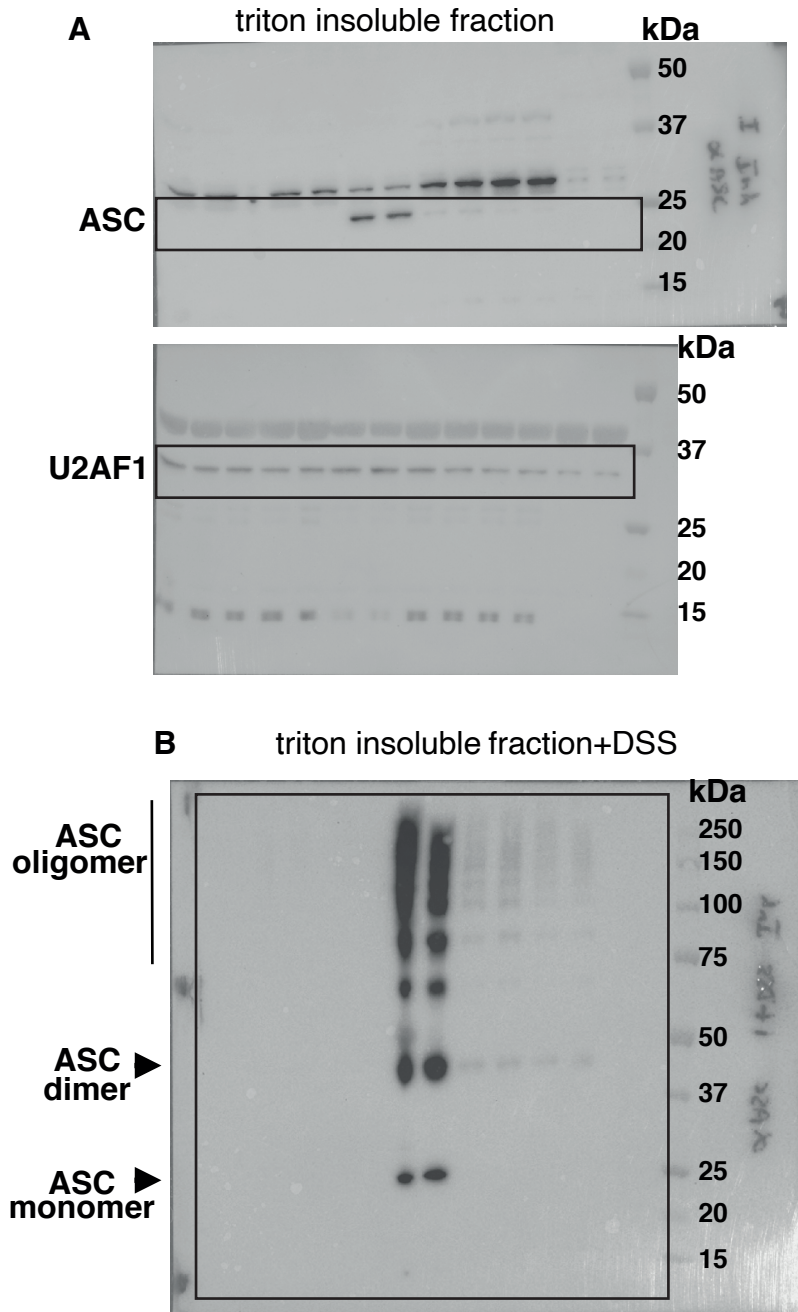

**Figure EV4**

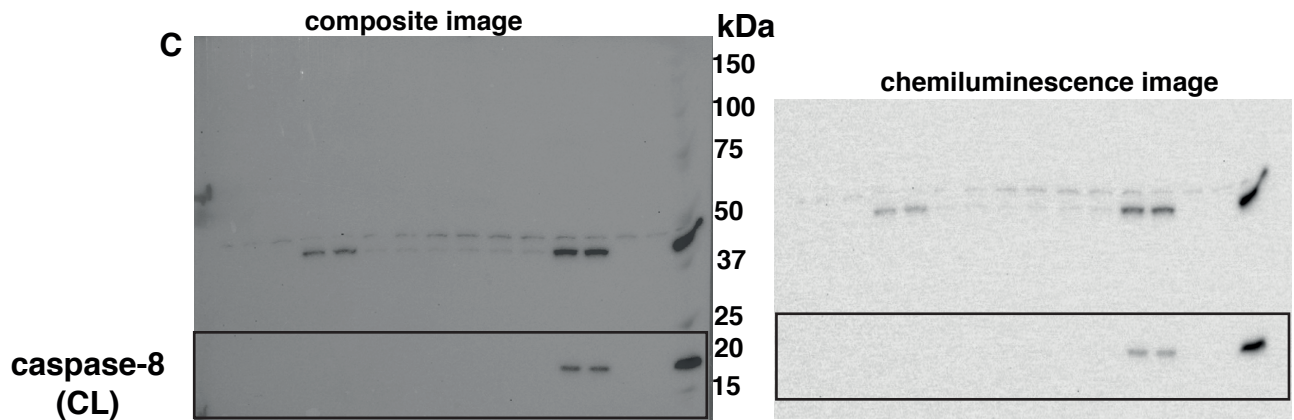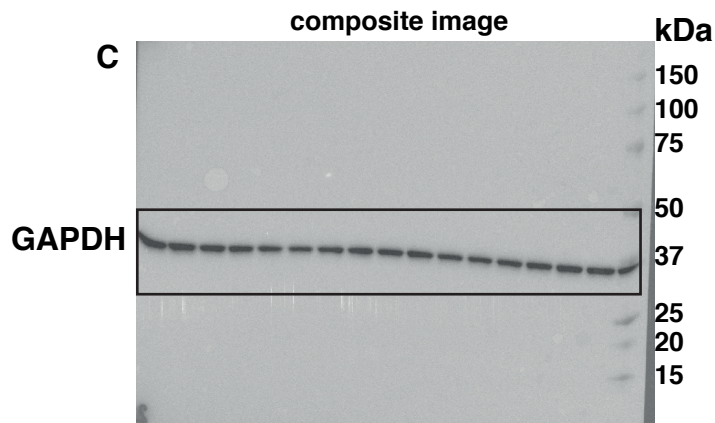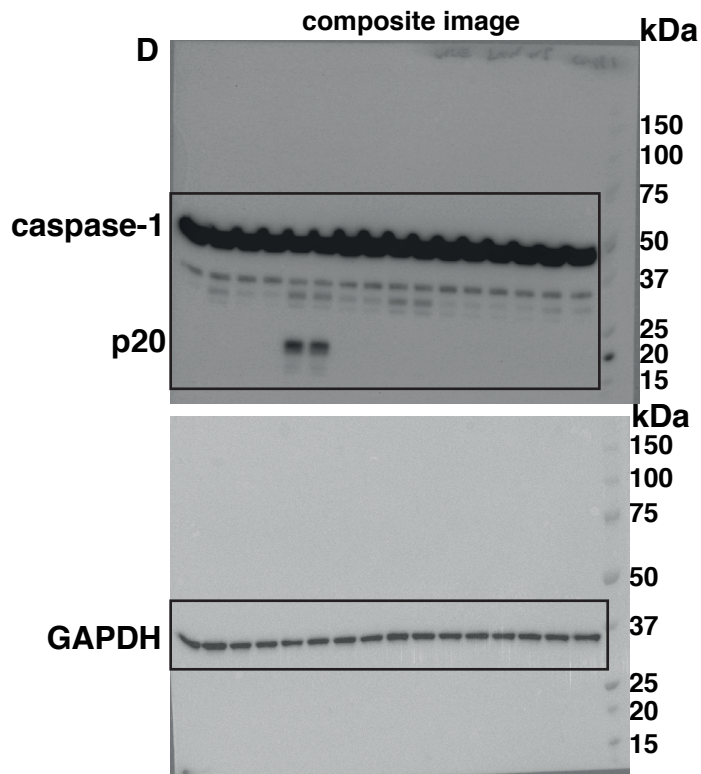

Supplement: Supplementary file 2 — Source Data for Expanded View [file EMBR-22-e50743-s007.zip › embr202050743-sup-0010-SDataFigEV4.pdf]

# Figure 1

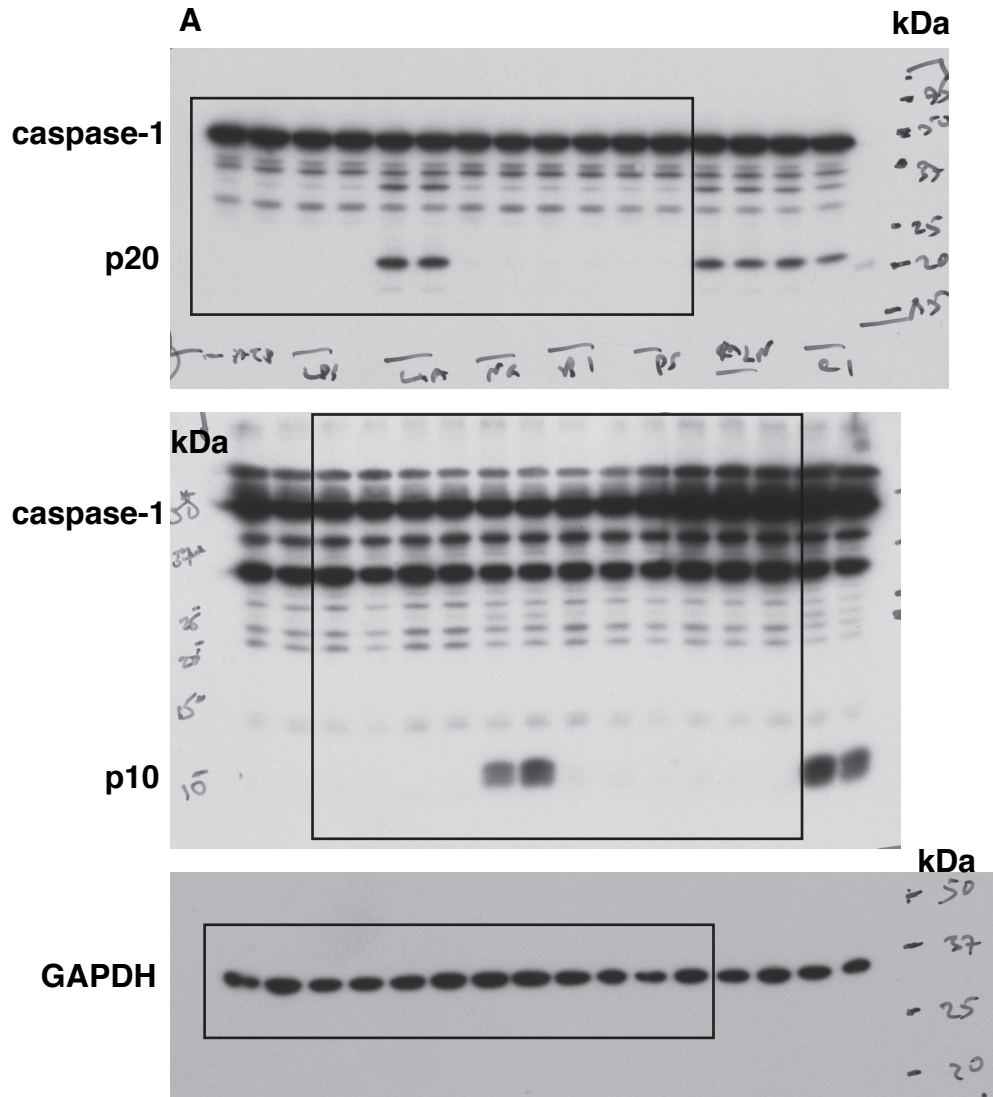

# Figure 1

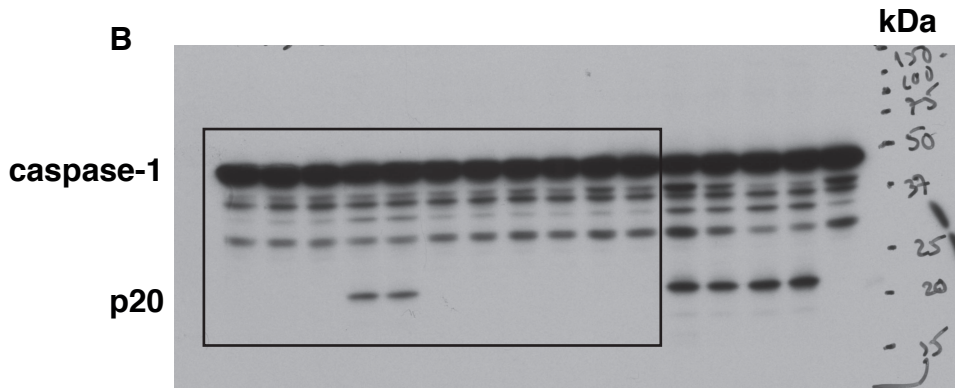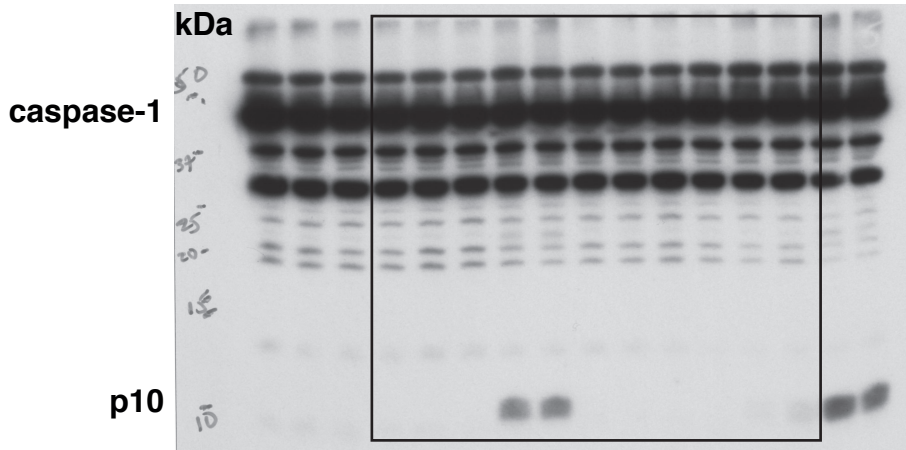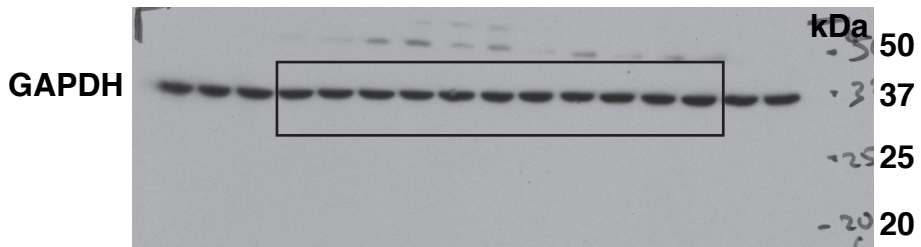

# Figure 1

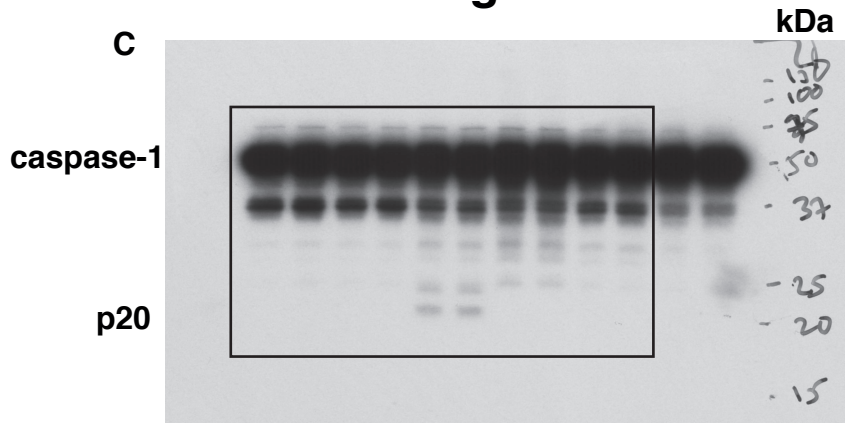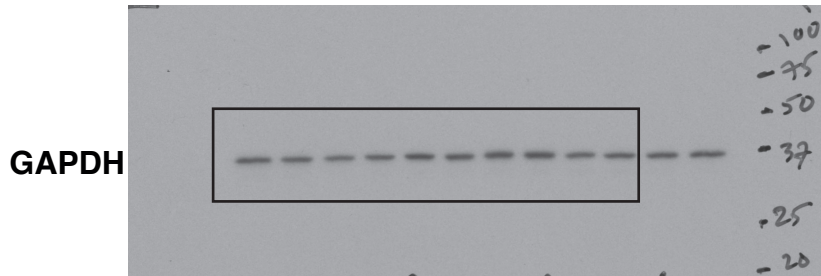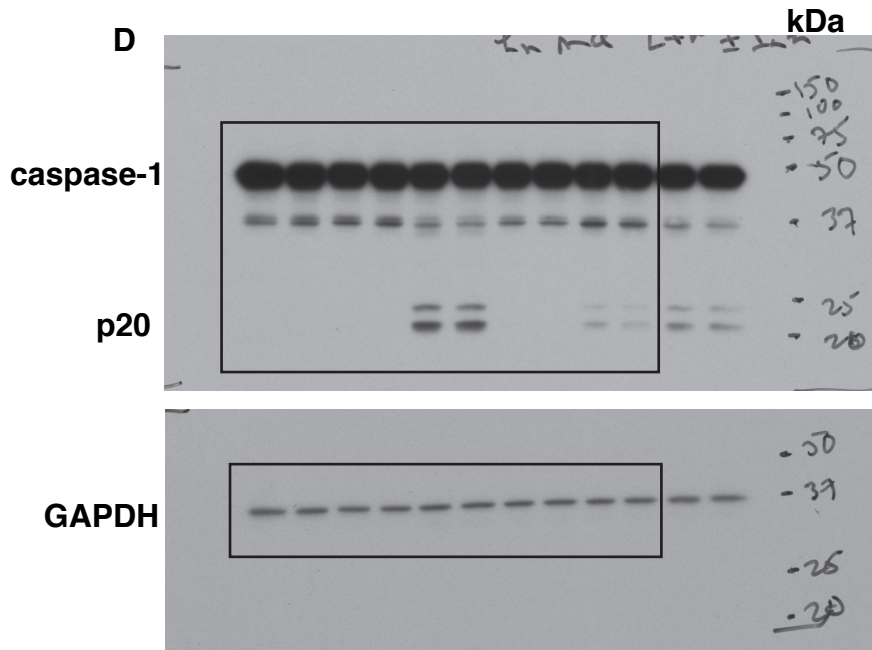

Supplement: Supplementary file 3 — Source Data for Figure 1 [file EMBR-22-e50743-s008.pdf]

# Figure 2

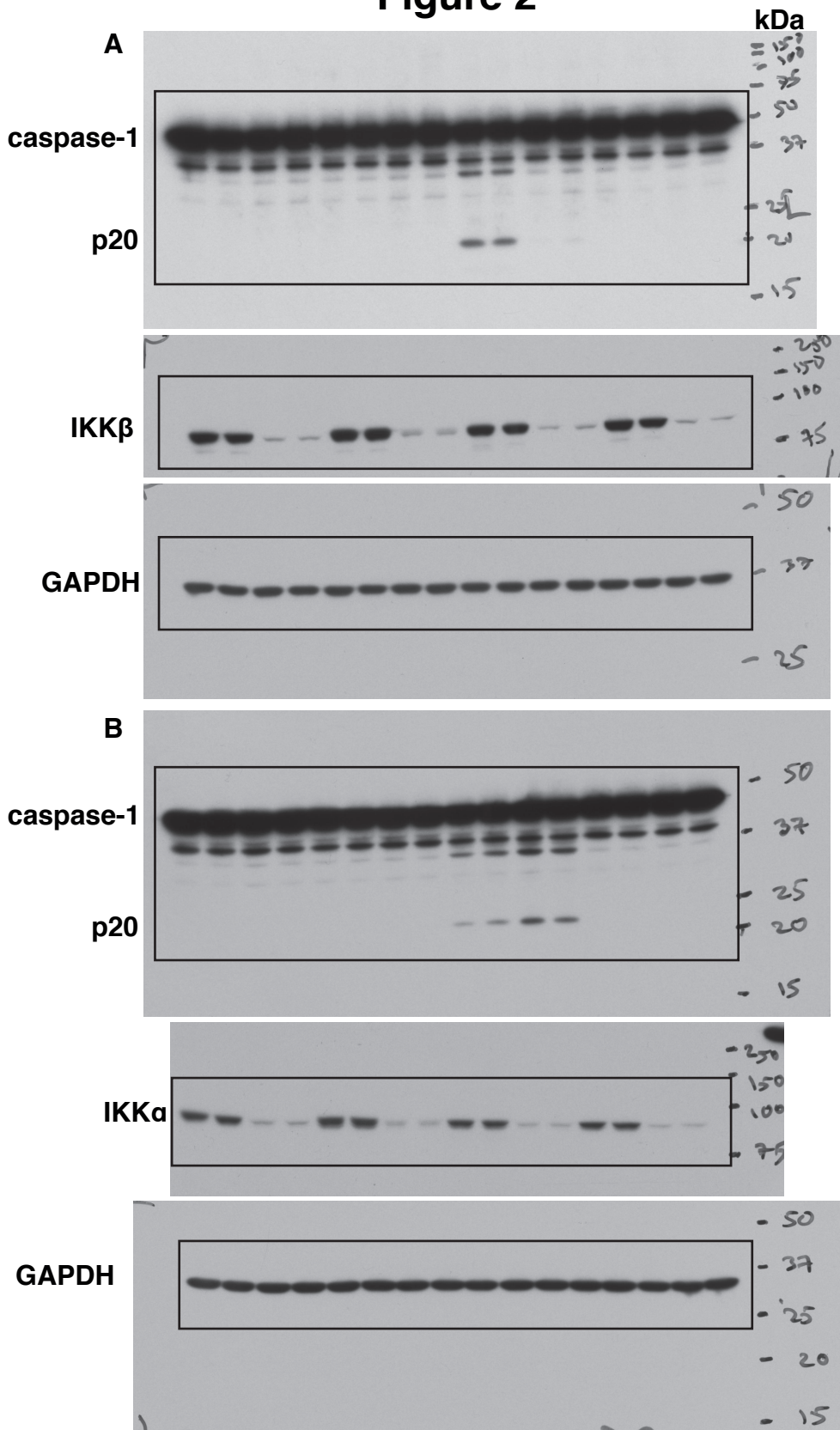

# Figure 2

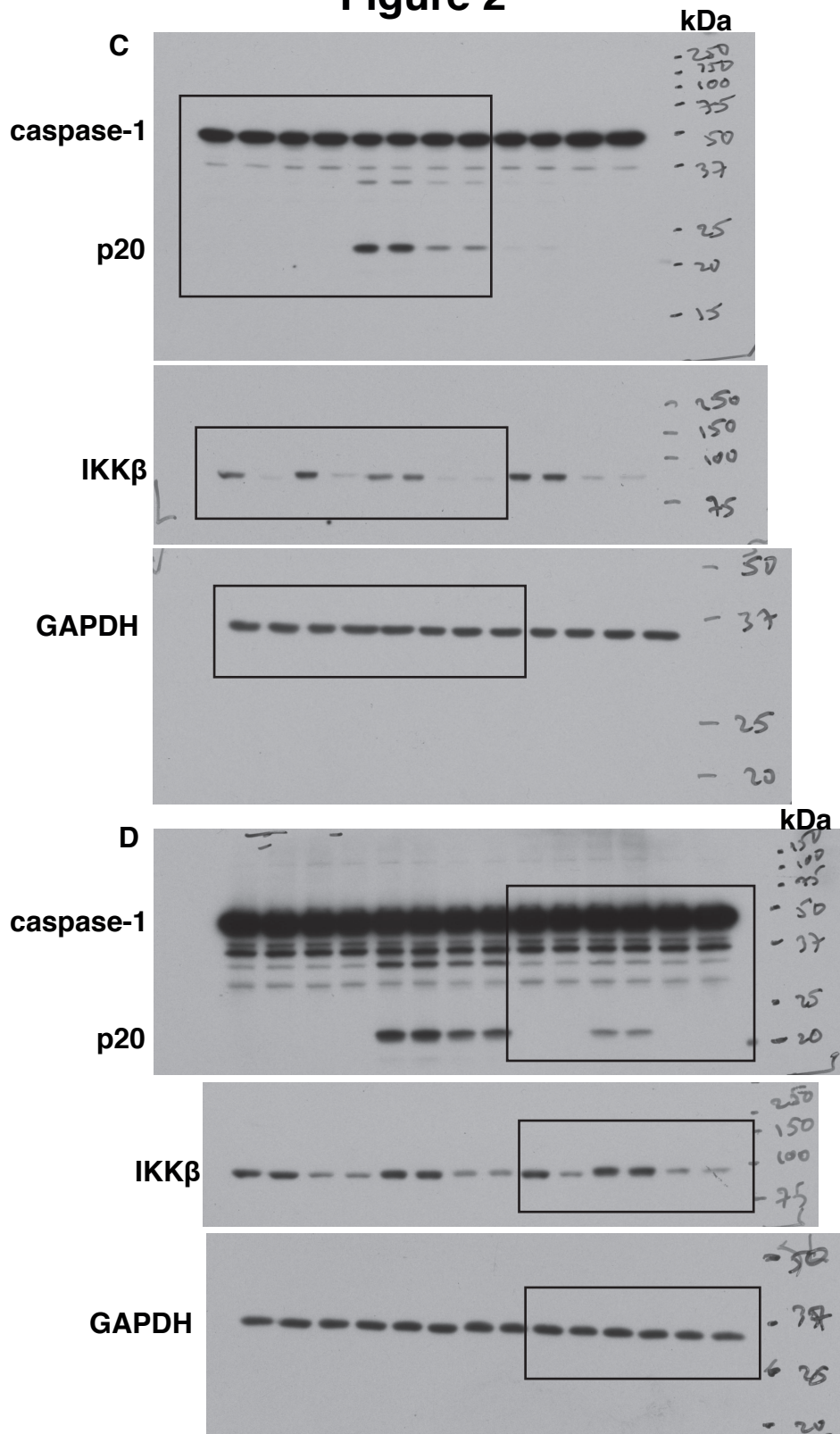

Supplement: Supplementary file 4 — Source Data for Figure 2 [file EMBR-22-e50743-s005.pdf]
