# Supplementary material for: IKKβ is required for the formation of the NLRP3 inflammasome
Source: EMBO Rep. 2021 Aug 17;22(10):e50743. doi: 10.15252/embr.202050743 (PMC8490994; doi:10.15252/embr.202050743)
Supplement: Supplementary file 5 — Source Data for Figure 3 [file EMBR-22-e50743-s002.pdf]

Figure 3

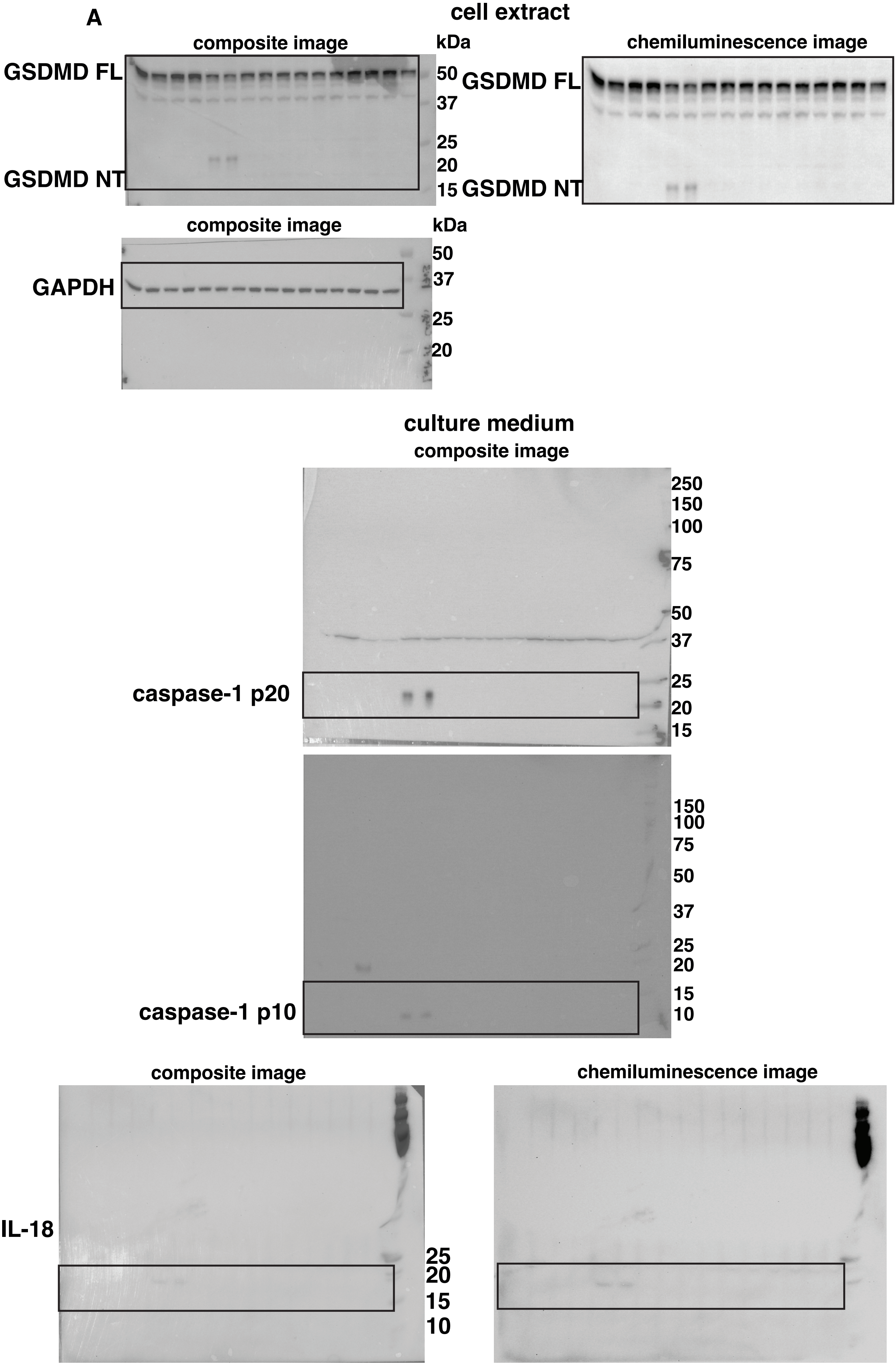
A
cell extract
composite image
kDa
GSDMD FL
GSDMD NT
50
37
25
20
15
chemiluminescence image
GSDMD FL
GSDMD NT
composite image
kDa
GAPDH
50
37
25
20
culture medium
composite image
250
150
100
75
50
37
25
20
15
caspase-1 p20
150
100
75
50
37
25
20
15
10
caspase-1 p10
composite image
chemiluminescence image
IL-18
25
20
15
10

Figure 3

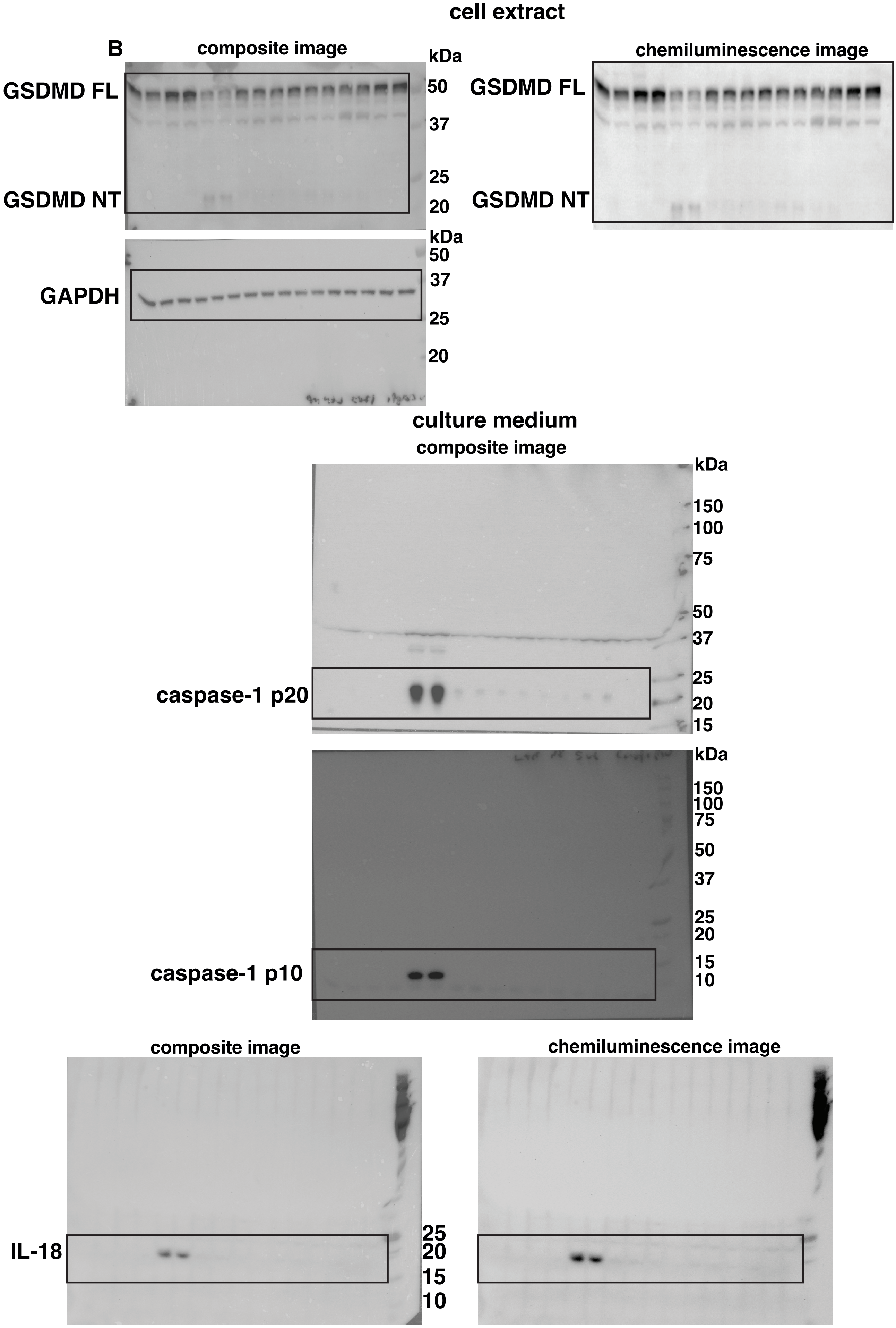
cell extract
B
composite image
chemiluminescence image
kDa
GSDMD FL
GSDMD NT
50
37
25
20
GAPDH
culture medium
caspase-1 p20
caspase-1 p10
150
100
75
15
10
IL-18

Figure 3

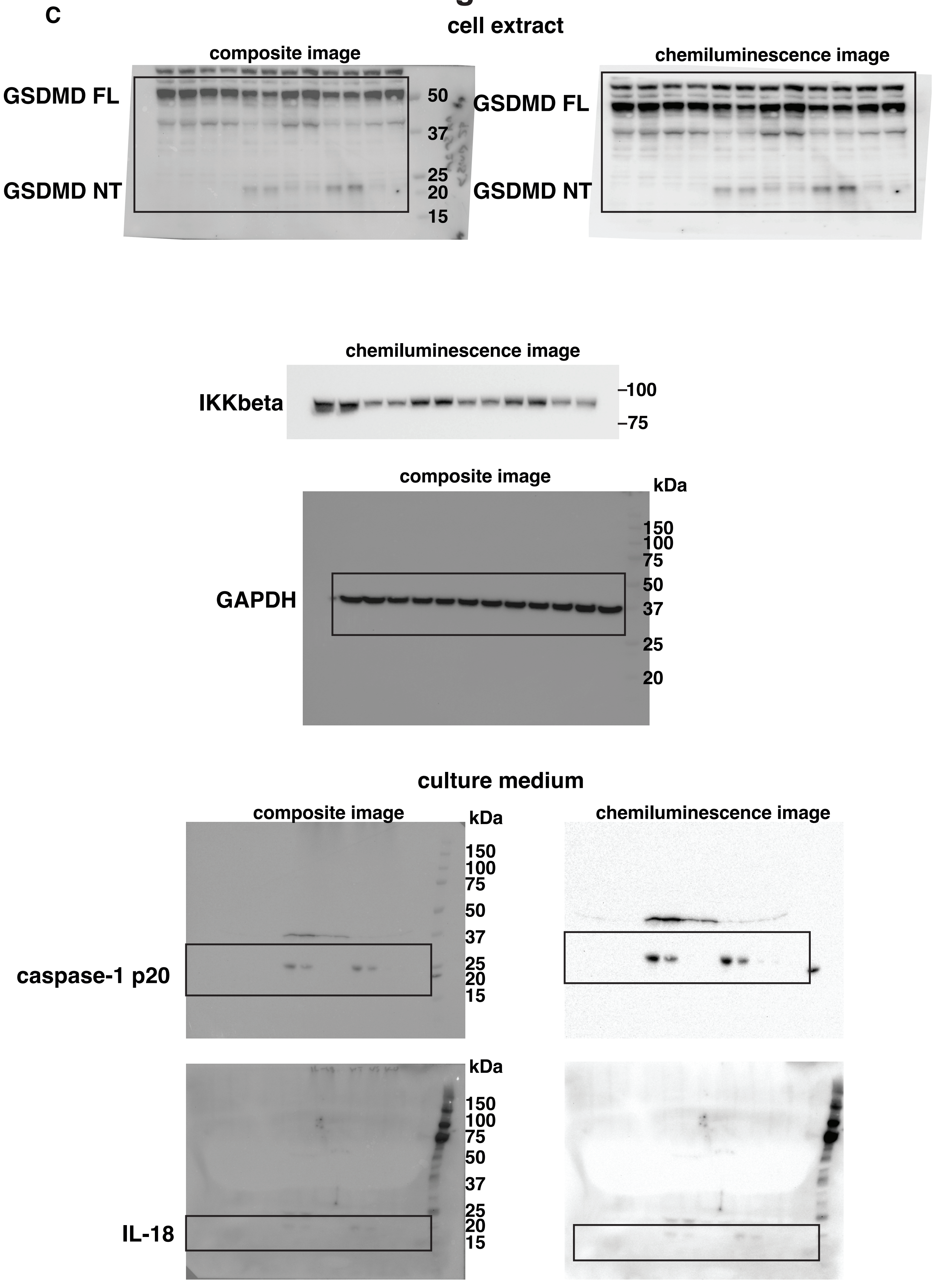

C
cell extract
composite image
chemiluminescence image
GSDMD FL
GSDMD NT
50
37
25
20
15
GSDMD FL
GSDMD NT
chemiluminescence image
IKKbeta
–100
–75
composite image
kDa
GAPDH
150
100
75
50
37
25
20
culture medium
composite image
kDa
150
100
75
50
37
25
20
15
caspase-1 p20
chemiluminescence image
kDa
150
100
75
50
37
25
20
15
IL-18
