# Supplementary material for: IKKβ is required for the formation of the NLRP3 inflammasome
Source: EMBO Rep. 2021 Aug 17;22(10):e50743. doi: 10.15252/embr.202050743 (PMC8490994; doi:10.15252/embr.202050743)

Figure 4

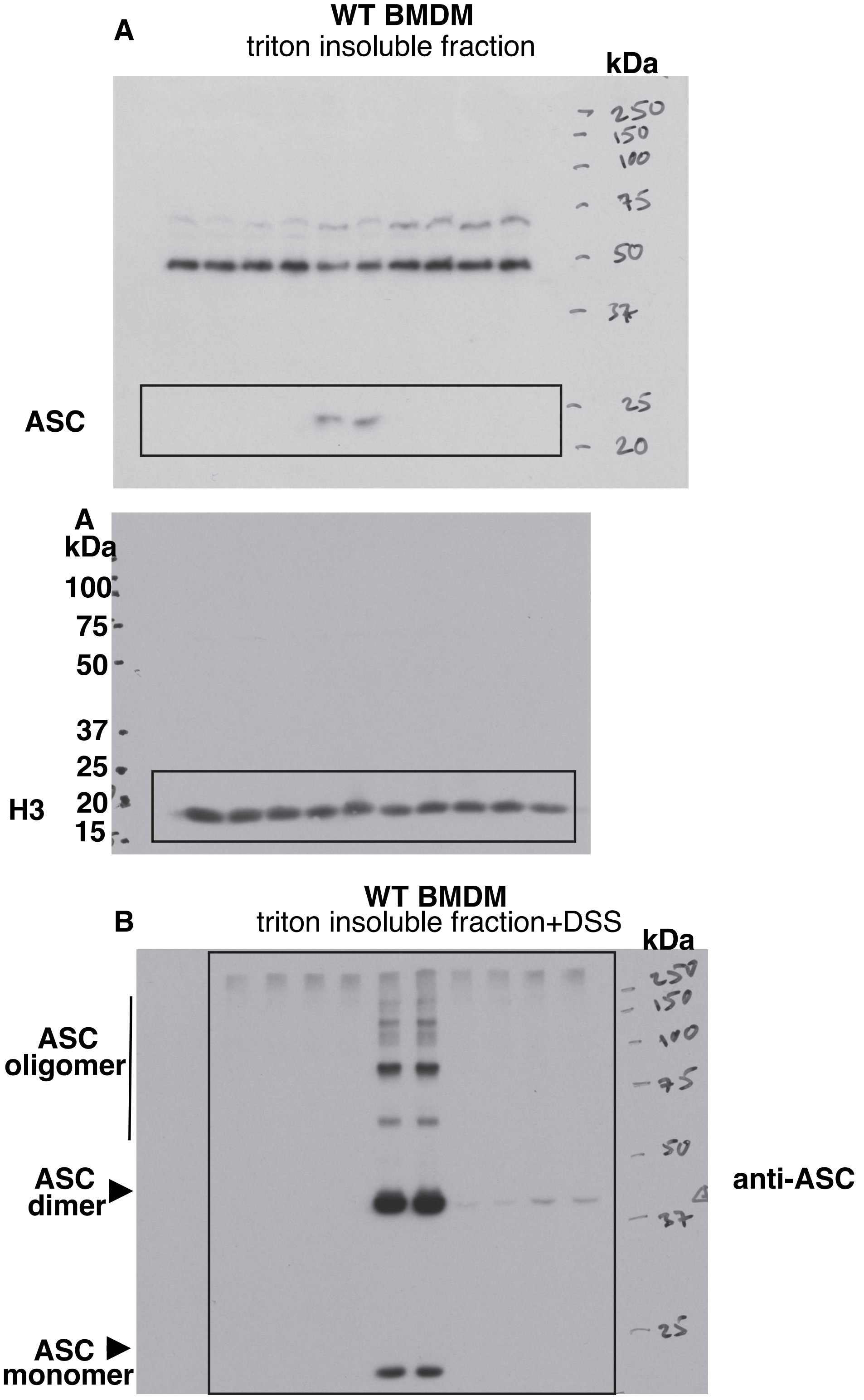
A
WT BMDM
triton insoluble fraction
kDa
250
150
100
75
50
37
25
20
ASC
A
kDa
100
75
50
37
25
20
15
H3
B
WT BMDM
triton insoluble fraction+DSS
kDa
250
150
100
75
50
37
25
ASC oligomer
ASC dimer
ASC monomer
anti-ASC

Figure 4

IKKβ (fl/fl) iBMDM
triton insoluble fraction

C

composite image

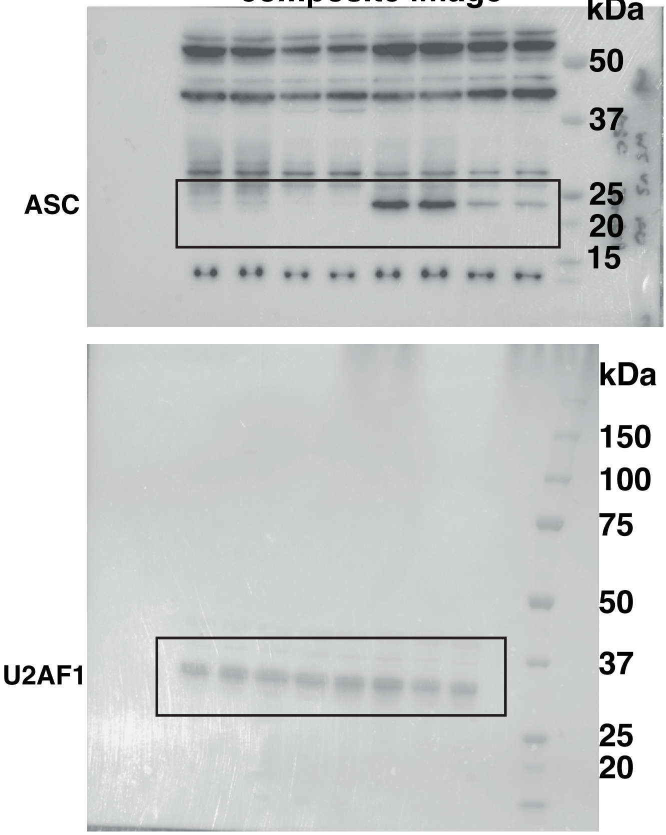

IKKβ (fl/fl) iBMDM
D triton insoluble fraction+DSS

composite image **chemiluminescence image**

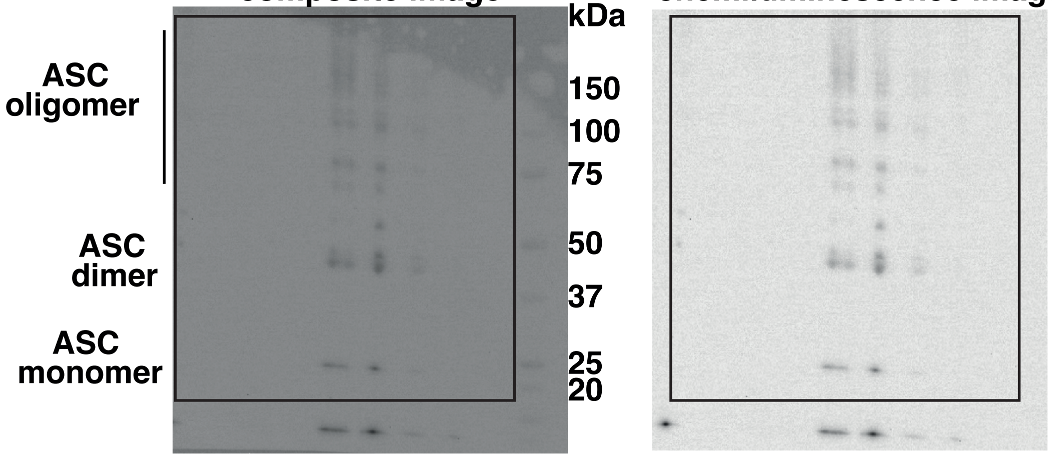

Figure 4

E

caspase-1 KO BMDM
triton insoluble fraction

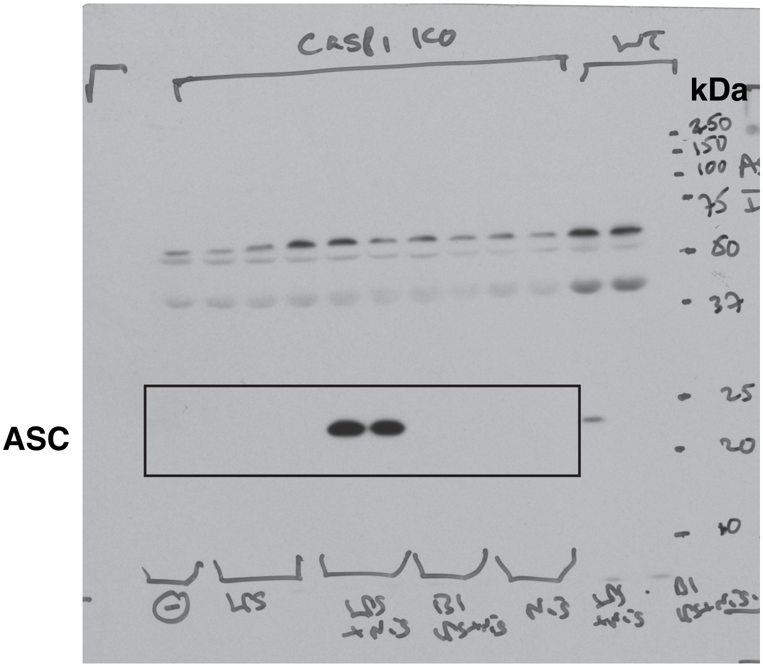

F

caspase-1 KO BMDM
triton insoluble+DSS

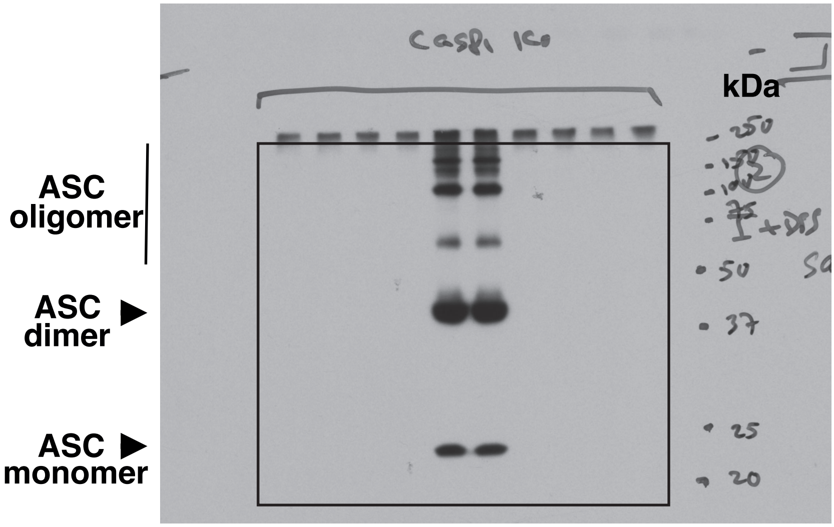

Supplement: Supplementary file 6 — Source Data for Figure 4 [file EMBR-22-e50743-s003.pdf]
