# Supplementary material for: IKKβ is required for the formation of the NLRP3 inflammasome
Source: EMBO Rep. 2021 Aug 17;22(10):e50743. doi: 10.15252/embr.202050743 (PMC8490994; doi:10.15252/embr.202050743)
Supplement: Supplementary file 8 [file EMBR-22-e50743-s004.pdf]

EMBO reports

IKKbeta is required for the formation of the NLRP3 inflammasome

Sambit Nanda, Alan Prescott, Clara Figueras-Vadillo, and Philip Cohen
DOI: 10.15252/embr.202050743

Corresponding author(s): Sambit Nanda (s.k.nanda@dundee.ac.uk) , Philip Cohen (p.cohen@dundee.ac.uk)

Review Timeline:

Submission Date:	27th Apr 20
Editorial Decision:	26th May 20
Revision Received:	28th May 21
Editorial Decision:	7th Jul 21
Revision Received:	26th Jul 21
Accepted:	30th Jul 21

Editor: Achim Breiling

Transaction Report:

(Note: With the exception of the correction of typographical or spelling errors that could be a source of ambiguity, letters and reports are not edited. Depending on transfer agreements, referee reports obtained elsewhere may or may not be included in this compilation. Referee reports are anonymous unless the Referee chooses to sign their reports.)

1st Editorial Decision — 26th May 2020

Dear Dr. Nanda,

Thank you for the submission of your research manuscript to EMBO reports. We have now received reports from the three referees that were asked to evaluate your study, which can be found at the end of this email.

As you will see, all referees think that the findings are of interest, but they also have several comments, concerns and suggestions, indicating that a major revision of the manuscript is necessary to allow publication in EMBO reports. As the reports are below, and I think all points need to be addressed, I will not detail them here.

Given the constructive referee comments, we would like to invite you to revise your manuscript with the understanding that all referee concerns must be addressed in the revised manuscript and in a detailed point-by-point response. Acceptance of your manuscript will depend on a positive outcome of a second round of review. It is EMBO reports policy to allow a single round of revision only and acceptance of the manuscript will therefore depend on the completeness of your responses included in the next, final version of the manuscript.

Revised manuscripts should be submitted within three months of a request for revision. We are aware that many laboratories cannot function at full efficiency during the current COVID-19/SARS-CoV-2 pandemic and we have therefore extended our 'scooping protection policy' to cover the period required for full revision. Please contact me to discuss the revision should you need additional time, and also if you see a paper with related content published elsewhere.

When submitting your revised manuscript, please also carefully review the instructions that follow below.

PLEASE NOTE THAT upon resubmission revised manuscripts are subjected to an initial quality control prior to exposition to re-review. Upon failure in the initial quality control, the manuscripts are sent back to the authors, which may lead to delays. Frequent reasons for such a failure are the lack of the data availability section (please see below) and the presence of statistics based on n=2 (the authors are then asked to present scatter plots or provide more data points).

When submitting your revised manuscript, we will require:

1) a .docx formatted version of the final manuscript text (including legends for main figures, EV figures and tables), but without the figures included. Please make sure that changes are highlighted to be clearly visible. Figure legends should be compiled at the end of the manuscript text.

2) individual production quality figure files as .eps, .tif, .jpg (one file per figure), of main figures and EV figures. Please upload these as separate, individual files upon re-submission.

The Expanded View format, which will be displayed in the main HTML of the paper in a collapsible format, has replaced the Supplementary information. You can submit up to 5 images as Expanded View. Please follow the nomenclature Figure EV1, Figure EV2 etc. The figure legend for these should be included in the main manuscript document file in a section called Expanded View Figure Legends after the main Figure Legends section. Additional Supplementary material should be supplied as a single pdf file labeled Appendix. The Appendix should have page numbers and needs

to include a table of content on the first page (with page numbers) and legends for all content. Please follow the nomenclature Appendix Figure Sx, Appendix Table Sx etc. throughout the text, and also label the figures and tables according to this nomenclature.

For more details please refer to our guide to authors:
http://www.embopress.org/page/journal/14693178/authorguide#manuscriptpreparation

See also our guide for figure preparation:
http://wol-prod-cdn.literatumonline.com/pb-assets/embo-site/EMBOPress_Figure_Guidelines_061115-1561436025777.pdf

3) a .docx formatted letter INCLUDING the reviewers' reports and your detailed point-by-point responses to their comments. As part of the EMBO Press transparent editorial process, the point-by-point response is part of the Review Process File (RPF), which will be published alongside your paper.

4) a complete author checklist, which you can download from our author guidelines (https://www.embopress.org/page/journal/14693178/authorguide). Please insert page numbers in the checklist to indicate where the requested information can be found in the manuscript. The completed author checklist will also be part of the RPF.

Please also follow our guidelines for the use of living organisms, and the respective reporting guidelines: http://www.embopress.org/page/journal/14693178/authorguide#livingorganisms

5) that primary datasets produced in this study (e.g. RNA-seq, ChIP-seq and array data) are deposited in an appropriate public database. This is now mandatory (like the COI statement). If no primary datasets have been deposited in any database, please state this in this section (e.g. 'No primary datasets have been generated and deposited').

See also: http://embor.embopress.org/authorguide#datadeposition

Please remember to provide a reviewer password if the datasets are not yet public.

The accession numbers and database should be listed in a formal "Data Availability " section (placed after Materials & Methods) that follows the model below. Please note that the Data Availability Section is restricted to new primary data that are part of this study.

Data availability

The datasets produced in this study are available in the following databases:

- RNA-Seq data: Gene Expression Omnibus GSE46843 (https://www.ncbi.nlm.nih.gov/geo/query/acc.cgi?acc=GSE46843)
- [data type]: [name of the resource] [accession number/identifier/doi] ([URL or identifiers.org/DATABASE:ACCESSION])

*** Note - All links should resolve to a page where the data can be accessed. ***

Moreover, I have these editorial requests:

6) We strongly encourage the publication of original source data with the aim of making primary data more accessible and transparent to the reader. The source data will be published in a separate source data file online along with the accepted manuscript and will be linked to the relevant figure. If you would like to use this opportunity, please submit the source data (for example scans of entire gels or blots, data points of graphs in an excel sheet, additional images, etc.) of your key experiments together with the revised manuscript. If you want to provide source data, please include size markers for scans of entire gels, label the scans with figure and panel number, and send one PDF file per figure.

7) Our journal encourages inclusion of *data citations in the reference list* to directly cite datasets that were re-used and obtained from public databases. Data citations in the article text are distinct from normal bibliographical citations and should directly link to the database records from which the data can be accessed. In the main text, data citations are formatted as follows: "Data ref: Smith et al, 2001" or "Data ref: NCBI Sequence Read Archive PRJNA342805, 2017". In the Reference list, data citations must be labeled with "[DATASET]". A data reference must provide the database name, accession number/identifiers and a resolvable link to the landing page from which the data can be accessed at the end of the reference. Further instructions are available at:
http://www.embopress.org/page/journal/14693178/authorguide#referencesformat

8) Regarding data quantification and statistics, can you please specify, where applicable, the number "n" for how many independent experiments (biological replicates) were performed, the bars and error bars (e.g. SEM, SD) and the test used to calculate p-values in the respective figure legends. Please provide statistical testing where applicable, and also add a paragraph detailing this to the methods section. See:
http://www.embopress.org/page/journal/14693178/authorguide#statisticalanalysis

Please note that all corresponding authors are required to supply an ORCID ID for their name upon submission of a revised manuscript. Please find instructions on how to link the ORCID ID to the account in our manuscript tracking system in our Author guidelines:
http://www.embopress.org/page/journal/14693178/authorguide#authorshipguidelines

I look forward to seeing a revised version of your manuscript when it is ready. Please let me know if you have questions or comments regarding the revision.

Yours sincerely,

Achim Breiling
Editor
EMBO Reports

Referee #1:

In the manuscript entitled "IKKβ is required for dispersion of the trans-Golgi network and activation of the NLRP3 inflammasome" the authors described an unusual role of IKKβ in the LPS-mediated rapid NLRP3 inflammasome formation and caspase-1 activation. They showed that IKKβ activity is required for the TNG38-positive puncta and that the role of IKKβ is independent of NFκB or protein kinases activated by IKKβ.

In addition to clearly presented experiments and well written manuscript, the authors provided

some new findings. However, this study is preliminary. The major issues are related to the fact that majority of conclusions are based on the experiments in which inhibitors were used. There are also some major concerns about the mechanism part, which need to be addressed.

Below are specific comments:

1. In addition to the inhibitors used in this study the authors should do more experiments with IKKβ KO cells (now IKKβ KD or KO is only used in Fig. 2 to show impaired caspase-1 processing).

2. Authors claimed that IKKβ activity is required for the TNG38-positive puncta (based on inhibitor use) - to confirm these results they should generate IKKβ kinase inactive cells or do put back experiments to IKKβ KO cells (WT and kinase mutant).

3. The study did not address in vivo biological importance of their findings.

4. The data in Fig. 5 is not sufficient to conclude that IKKβ is required for the dispersion of TGN. More sophisticated assay is required here, such as electron microscope analysis. Furthermore, also shown in Fig. 5C and in the original report from Dr. Chen's group (Chen & Chen, 2018), ATP or nigericin stimulation is the major signaling for the dispersion of TGN, not LPS. Then, what is role of IKKβ under ATP/nigericin stimulation? Would IKKβ inhibitor block ATP/nigericin-induced TGN dispersion? Is IKKβ required for the interaction between NLRP3 and PtdIns4P? Does IKKβ translocate to TGN?

5. This study aims to elucidate the role of IKKβ in rapid inflammasome activation (LPS+ATP combine treatment for 30 min), not classical/chronic inflammasome activation (4h LPS priming plus ATP or nigericin for another 30 min). However, LPS 4h priming plus 60 min nigericin treatment condition was used in Fig. 5. It raises more concern about the TGN dispersion story. Does LPS+ATP combine treatment for 30 min induce TGN dispersion at all?

6. Recent studies show that TAK1 deficiency results in spontaneous inflammasome activation (J Exp Med (2018) 215 (4): 1023-1034.) and TAK1 inhibition leads to alternative Caspase-8 activation (PNAS November 13, 2018 115 (46)). In Fig.1, blocking IKKβ upstream kinase TAK1 abolishes Caspase-1 activation. Is Caspase-8 cleaved here? The author should also examine pro-IL-1 cleavage by western blot. Is pyroptosis activated in the rapid inflammasome activation condition?

7. The authors were trying to investigate whether IKKβ facilitate TGN dispersion by activating small GTPase. Using bacterial enzyme CN04/CN03 to activate GTPase activity allows ATP or nigericin to activate inflammasome without LPS stimulation. Does it suggest that LPS leads GTPase activation through IKKβ? Does IKKβ inhibitor block CN04/CN03-induced caspase-1 cleavage? Does CN04/CN03 treatment lead to TGN dispersion or IKKβ activation?

Referee #2:

In this manuscript the authors investigate the role of IKKβ in controlling the formation and function of the NLRP3 inflammasome. Using a panel of pharmacological inhibitors they demonstrate that formation of the inflammasome does not require transcription or translation; however, the findings indicate that IKKβ plays a key role in this process. They further show that IKKβ regulates the dispersion of the TGN and they conclude that this role of IKKβ is separate from its function in the

canonical NF-κB pathway. Finally, the data suggest that IKKβ might regulate caspase-1 activity by targeting proteins that lock Rho-GTPases into their active state. Overall the experiments are extremely well performed and the biochemical data are of the highest quality. However, in its current form the findings are quite preliminary and the mechanism proposed is not fully supported by the data presented. There are a number of weaknesses outlined below that detract from the impact and significance of the study; however, the central finding that inhibiting IKKβ blocks the formation and function of the inflammasome is exciting and of wide interest in the field

Major points:

1. The study relies very heavily on pharmacological inhibitors of IKKβ and other kinases. Further use of the genetic models available to the authors would add strong support to the overall conclusions. Moreover, the first two figures in the paper employ three separate inhibitors (one TAK1 inhibitor and two distinct IKKβ blockers) and this significantly strengthens the validity of these experiments. However, the remainder of the manuscript relies only on one compound (BI605906) thereby lessening the confidence that the effects are indeed specific for the TAK1-IKKβ signaling pathway.
2. The data shown in Figure 3 do not add any mechanistic insight to the study. As noted above, the experiments are performed using only one inhibitor if IKKβ so any conclusions regarding the relative activity of the kinase in controlling NLRP3 v NF-κB activation are dependent on the results of only one pharmacological approach. Moreover, the authors use phosphorylation of NF-kB1 as the means to assess activation of the NF-κB pathway. The central mechanism controlling canonical NF-κB activation that is the primary function of IKKβ in the pathway, is phosphorylation of IkBa. Without extensive further pharmacokinetic analysis of IkB phosphorylation and NF-κB activation it is impossible to conclude from the experiments shown that there is a discernible difference in the activity of IKKβ in controlling NF-κB activation versus NLRP3.
3. The data shown in Figure 5 are interesting but do not definitively establish the link between IKKβ activity and activation of the inflammasome.
4. The potential role of IKKβ in regulating Rho-GTPase function is implied but not established.

Minor:
1. The last section of the results describing the lack of effects on the AIM2 inflammasome would be better placed earlier in the manuscript.
2. The final paragraph of the results section is confusing and belongs in the discussion.
3. The labeling of Fig EV1A does not appear to be correct. Lanes 7 and 8 should be + for LPS.

Referee #3:

This article by Nanda and colleagues extends on the recent work of James Chen's laboratory to report that IKKb is required for the dispersion of the trans-Golgi network, and resultant activation of the NLRP3 inflammasome. While the article raises several interesting points, there are several major and minor points that currently preclude publication in EMBO reports.

Major concerns:
1. Throughout the document, the authors analyse cell extracts for signs of inflammasome signaling (i.e. generation of p20 caspase-1) and presume that their absence means that inflammasome signaling was blocked. However, p20 is released from the cell extract into the cell culture media when cells lyse by pyroptosis. Thus, lack of p20 in cell extracts could be coupled to more p20 in cell supernatants, which would indicate augmented pyroptosis, rather than decreased inflammasome

signaling. The authors should thus probe for caspase-1 in cell supernatants for all immunoblot experiments, to show that supernatants show a similar profile to cell extracts.
2. Figure 5 is the key figure supporting the authors assertions. It shows that LPS+nigericin (but not control stimulations) trigger the redistribution of TGN38 into large puncta, which is blocked by the IKKbeta inhibitor BI605906. This figure would lend much stronger support to the authors' assertions that IKKb is required for TGN dispersal if it included a more specific method of IKKb antagonism (e.g. IKKb siRNA versus control siRNA, which the authors already use elsewhere). The authors also state that CN03 and CN04 can perform the same function as IKKb in triggering TGN dispersal; if this is the case, then adding CN03 and 04 to IKKb-silenced cells should restore dTGN puncta. Inclusion of these extra samples (with reagents and methods already at hand) would greatly strengthen the authors' hypotheses.
3. The significance/interpretation of Figure EV5 is unclear. Are the authors suggesting that the "perinuclear vesicles" represent dTGN? Is it not possible that these are ASC specks? Without IF staining accompanying the bright field images, it is difficult to assess what these structures are, and how to interpret these data.
4. Figure EV1 actD and cyx experiments should display NLRP3 blots so that the reader can see that NLRP3 levels are affected by these treatments.
5. Throughout the document there are statements like (p3) "the NLRP3 inflammasome, whose activation requires both a PAMP and a DAMP". This is incorrect. Indeed, NLRP3 activation follows a 2 step mechanism, but there is no requirement for signal 1 and 2 to be fulfilled by a PAMP followed by a DAMP. Both signal 1 and signal 2 can be PAMPs (e.g. LPS + nigericin, LPS+LLO). Signal 1 can be also be a cytokine (which can be a DAMP/alarmin such as IL-1a, or may not be, such as TNF, or IL-1b). So cytokine + DAMP (e.g. TNF+ATP), DAMP+DAMP (e.g. IL-1a + ATP) and cytokine + PAMP (e.g. TNF + nigericin) combinations of signal 1 and 2 are also possible. Please fix all of these sentences as they are confusing and incorrect. A related point (p10) "PAMP plus DAMP activation of the NLRP3 inflammasome that we have studied in this paper" refers to LPS+nigericin, which is PAMP+PAMP not PAMP+DAMP. Would be simpler to just say "LPS plus nigericin activation of the NLRP3 inflammasome that we have studied in this paper".
6. Some statements referring to "inflammasome priming" are clearly referring to transcriptional upregulation of NLRP3. Instead of using the term priming, the authors should instead just say "transcriptional upregulation of NLRP3", because it is well appreciated in the field that there are other mechanisms of non-transcriptional priming (e.g. NLRP3 post-translational modifications) that poise NLRP3 for signaling. For example (p4) "However, it is now clear that TLR signalling is also required for the rapid phase of caspase-1 activation, which does not require priming (Fernandes-Alnemri et al, 2013; Lin et al, 2014)." Incidentally, I believe that PMID22898390 was the first article to show rapid TLR-mediated effects on NLRP3 activity (before the cited references were published).
7. Several western blot figures demonstrate cleavage of inflammasome-related proteins (e.g. caspase-1). These should be annotated with molecular weight markers so that the reader can assess for themselves that the cleavage fragments are indeed the correct size.
8. Please clarify the figure captions: (1) If signal 1 and 2 are co-administrated (and not sequentially administered) throughout the article's experiments, please change "administered" to "coadministered". (2) For some of captions it is unclear how many independent biological replicate data sets were performed.

Minor concerns:
1. Throughout the document nigericin is referred to as a pore-forming toxin, which is not strictly true (it is a potassium ionophore).
2. Abstract line 4: "allowing it to cleave IL-1" should be "allowing it to cleave IL-1beta".
3. Page 3 "The CARD domain can then recruit caspase-1 through CARD/CARD interactions, leading to the "autocleavage" of caspase-1", should be "The CARD domain can then recruit caspase-1

through CARD/CARD interactions, leading to the dimerization and autocleavage of caspase-1". Dimerization is important because this confers protease activity to caspase-1, such is needed for autocleavage. I do not see why autocleavage should be in inverted commas.

4. Page 7 referring to full length caspase-1 in triton insoluble fraction: "as well as aggregates of uncleaved caspase-1; this did not occur in cells treated with BI605906 (Fig 4A)". Inclusion of full length caspase-1 in the insoluble fraction does not necessarily mean that caspase-1 is aggregated (for example, it could be attached to the cytosolic face of an organelle, or the purity of the fraction may be questionable). In fact, it is likely that this fraction has captured monomeric caspase-1, as caspase-1 p45 is present at similar levels throughout all stimulations (including inflammasome negative controls).

5. Figure EV1a labels suggest that caspase-1 p20 is generated in unprimed cells treated with ATP and ActD. I presume this is a mistake? Should the LPS row be marked + for these samples?

6. Figure EV1 data would be more compelling if LPS-induced (inflammasome-unrelated) control proteins were shown to be blocked by ActD/Cyx.

EMBO Reports Manuscript Number (EMBOR-2020-50743V1)

Dear Editor,

Thank you for sending the three reviews of our paper and for inviting resubmission of a revised version of the article We thank the Editorial team for providing the additional time needed for revision during the difficult situation created by the COVID-19 pandemic. We are pleased that the reviewers found our paper to be interesting, clearly presented and well written. We have found their comments valuable, which has enabled us to strengthen the manuscript significantly. We are now re-submitting a revised version, in which we believe that we have addressed all of the concerns of reviewers 2 and 3 and most of the concerns of reviewer 1. The revised manuscript contains six new figures (the new Figs 3, 5 and 6, EV5, and appendix Figures 1 and 2). Fig 4 also has additional new data presented in panels C and D. Fig EV1 has additional new data presented in panels C, D, E and F and Fig EV4 has an additional panel E. Two figures in the version that was originally submitted (the original Figs 3 and 6) have been deleted for reasons explained in the detailed responses to the reviewers below. The major additions and changes to the paper, which are following, have been highlighted in the text, are as follows:-

1. We have changed the title to "IKKβ is required for the rapid activation of the NLRP3 inflammasome". Our paper shows that IKKβ is required for bringing NLRP3 to the close proximity of TGN38 protein on the dispersed TGN and for the formation and activation of the NLRP3 inflammasome and its consequences. It is not required for the nigericin-induced dispersion of the TGN.
2. We have carried out additional experiments with immortalized macrophages from IKKβ-CXCR3-Cre (flox/flox) mice in which the expression of IKKβ is reduced (Figs 3C, 4C, 4D, 5 (panels I-L and N), 6 (panels I-L), and which support the results obtained using IKKβ inhibitors. We have also carried out additional experiments with a third structurally unrelated inhibitor of IKKβ (TPCA-1) with similar results (Figs 3A, 3B, 5 and 6; Figs EV5, and Appendix Figures 1 and 2).
3. We have found that the rapid activation of the NLRP3 inflammasome triggers the conversion of gasdermin D to its active, cleaved form and that this is also prevented by IKKβ inhibitors and in immortalized BMDM from mice with reduced expression of IKKβ (Figs 3 and EV5).
4. We have performed further experimentation which shows that the secretion of the p20 and p10 fragments of caspase-1, and the secretion of IL-18 is suppressed by IKKβ inhibition and in immortalized BMDM from mice in which the expression of IKKβ is reduced (Figs 3 and EV5). These experiments show that the inhibition of IKKβ does not enhance the secretion of the p20 and p10 fragments of caspase-1.
5. We have performed new proximity ligation assays, which show that co-stimulation with LPS and nigericin for one hour brings NLRP3 to the close proximity of TGN38 protein on dispersed TGN, which is suppressed by inhibitors of IKKβ and in immortalized macrophages from mice in which the expression of IKKβ is reduced (Figure 5).

Our point-by-point response to each of the reviewer's comments is detailed below. The main changes are highlighted in yellow.

Referee #1:

In the manuscript entitled "IKKβ is required for dispersion of the trans-Golgi network and

activation of the NLRP3 inflammasome" the authors described an unusual role of IKKβ in the LPS-mediated rapid NLRP3 inflammasome formation and caspase-1 activation. They showed that IKKβ activity is required for the TGN38-positive puncta and that the role of IKKβ is independent of NFκB or protein kinases activated by IKKβ.

In addition to clearly presented experiments and well written manuscript, the authors provided some new findings. However, this study is preliminary. The major issues are related to the fact that majority of conclusions are based on the experiments in which inhibitors were used. There are also some major concerns about the mechanism part, which need to be addressed.

Below are specific comments:

1. In addition to the inhibitors used in this study the authors should do more experiments with IKKβ KO cells (now IKKβ KD or KO is only used in Fig. 2 to show impaired caspase-1 processing).
We have performed a number of additional experiments using immortalised macrophages from mice in which IKKβ expression is reduced, and which support the results obtained using IKKβ inhibitors (Figs 3C, 4C, 4D, 5 (panels I-L and 5N), Fig 6 (panels I-L). We have also performed some experiments using a third structurally unrelated IKKβ inhibitor (TPCA-1) again with similar results (Figs 3A, 3B, 5 (panels C, G and 5M), 6 (panels C and G), EV5 and Appendix Figures 1 and 2).
2. Authors claimed that IKKβ activity is required for the TNG38-positive puncta (based on inhibitor use) - to confirm these results they should generate IKKβ kinase inactive cells or do put back experiments to IKKβ KO cells (WT and kinase mutant).
We have performed new Proximity Ligation Assays, which have established that NLRP3 comes to close proximity of TGN38 protein during the rapid activation of the NLRP3 inflammasome induced by co-stimulation with LPS and nigericin, but not by stimulation with LPS or nigericin alone. The co-localisation of NLRP3 and TGN38 in primary BMDM is prevented by inhibitors of IKKβ and reduced in immortalised BMDM from mice expressing decreased levels of IKKβ (new figure 5). In the new Figure 6, studying the rapid activation of the NLRP3 inflammasome only, we show that IKKβ inhibitors have no effect on the dispersion of the TGN triggered by nigericin alone (Fig 6, panels G and H), but suppress the formation of the brighter puncta produced by co-stimulation with LPS plus nigericin (Fig. 6 panels B, C and D); i.e. the same conditions needed to observe co-localisation of NLRP3 with the dispersed TGN (Fig 5). The formation of these brighter and larger puncta is reduced in immortalised BMDM from IKKβ-CXCR3-Cre (flox/flox) mice.

Prolonged stimulation for 4 h with LPS in order to transcriptionally upregulate NLRP3, followed by stimulation for 1 h with nigericin in the continued presence of LPS, increases both the size and number of bright TGN-positive puncta, compared to those observed during the rapid activation of the inflammasome triggered by co-stimulation with for 1 h with LPS and nigericin (compare Fig 6 panel B with EV4 panel C). IKKβ inhibitors added after prolonged 4 h stimulation with LPS, but before stimulation with nigericin, suppressed the formation of the large bright puncta (Fig EV4, panels C-E).

3. The study did not address in vivo biological importance of their findings.
Our paper is focused on establishing an essential role for IKKβ in the formation and rapid activation of the NLRP3 inflammasome, which we believe we have achieved. The next stage of our project will be to identify the novel substrate(s) of IKKβ whose phosphorylation is required for the formation and activation of the NLRP3 inflammasome. Only when that has been accomplished it will be possible to investigate the phosphorylation of that protein(s) in vivo, whether it is triggered by the rapid activation of the NLRP3 inflammasome in vivo, and whether

it is prevented by IKKβ-inhibiting drugs with pharmaco-kinetic properties suitable for in vivo studies. With all due respect to the reviewer, we think that such experiments go well beyond the scope of the present study.

4. The data in Fig. 5 is not sufficient to conclude that IKKβ is required for the dispersion of TGN. More sophisticated assay is required here, such as electron microscope analysis. Furthermore, also shown in Fig. 5C and in the original report from Dr. Chen's group (Chen & Chen, 2018), ATP or nigericin stimulation is the major signalling for the dispersion of TGN, not LPS. Then, what is role of IKKβ under ATP/nigericin stimulation? Would IKKβ inhibitor block ATP/nigericin-induced TGN dispersion? Is IKKβ required for the interaction between NLRP3 and PtdIns4P? Does IKKβ translocate to TGN?

The new Proximity Ligation assays (PLA) that we have performed clearly show that IKKβ activity is required for the association of NLRP3 with TGN38 during the rapid activation of the NLRP3 inflammasome (Fig 5). We show in the new Fig 6 that IKKβ activity is not required for dispersion of the TGN induced by stimulation with nigericin alone. It is the formation of the brighter TGN positive foci formed consistently during co-stimulation with LPS and nigericin that is decreased by IKKβ inhibitors or in immortalised macrophages from mice expressing reduced levels of IKKβ (Fig 6). In the Discussion we briefly consider how IKKβ might exert these effects, but the precise molecular mechanism will require identification of the relevant IKKβ substrate(s) in future studies.

5. This study aims to elucidate the role of IKKβ in rapid inflammasome activation (LPS+ATP combine treatment for 30 min), not classical/chronic inflammasome activation (4h LPS priming plus ATP or nigericin for another 30 min). However, LPS 4h priming plus 60 min nigericin treatment condition was used in Fig. 5. It raises more concern about the TGN dispersion story. Does LPS+ATP combine treatment for 30 min induce TGN dispersion at all?

We have now used the PLA assay to show that NLRP3 co-localises with the TGN during the rapid activation of the NLRP3 inflammasome induced by co-stimulation with LPS and nigericin (Fig 5). We also show that nigericin alone causes rapid dispersion of the TGN, without any need for prolonged LPS-stimulated transcriptional upregulation of NLRP3. IKKβ is required to form the brighter and larger TGN38-positive foci induced by co-stimulation with LPS and nigericin (Fig 6). The new title of the resubmitted paper focuses on the key findings of our paper. The results section of the original version of the paper never showed that IKKβ was required for the dispersion of the Golgi. We apologise for the title of the original version of the paper, which was incorrect and misleading.

6. Recent studies show that TAK1 deficiency results in spontaneous inflammasome activation (J Exp Med (2018) 215 (4): 1023-1034.) and TAK1 inhibition leads to alternative Caspase-8 activation (PNAS November 13, 2018 115 (46)). In Fig.1, blocking IKKβ upstream kinase TAK1 abolishes Caspase-1 activation. Is Caspase-8 cleaved here? The author should also examine pro-IL-1 cleavage by western blot. Is pyroptosis activated in the rapid inflammasome activation condition?

The reviewer asks whether pyroptosis is activated during the rapid activation of the NLRP3 inflammasome. We assessed pyroptosis by studying the cleavage of gasdermin D to its active, cleaved fragment. We found that gasdermin D was activated during the rapid activation of the NLRP3 inflammasome by co-stimulation with LPS and either ATP or nigericin, and that this was blocked by inhibition of IKKβ or in immortalised BMDM from mice expressing reduced levels of IKKβ (Fig 3). The activation of gasdermin under these conditions is presumably mediated by caspase-1, as reported by others (see new references, Kayagaki et al, 2015; Shi et al, 2015).

There is no pro-IL-1β present during the rapid activation of the NLRP3 inflammasome so pro-IL-1 cleavage cannot be studied. Instead, we studied the secretion of IL-18 during the rapid activation of the NLRP3 inflammasome, which requires activation of the pore-forming protein gasdermin D. As expected, the activation of caspase-1 and gasdermin D and the secretion of IL-18

were all suppressed by the inhibition of IKKβ and reduced in immortalised BMDM from mice expressing reduced levels of IKKβ. We thank the reviewer for these questions which have prompted us to make some additional interesting findings that have strengthened the paper. These results are shown in Figure 3 of the revised paper.

To measure the secretion of IL-1β we first induced its expression by prolonged stimulation with LPS alone, incubated the cells with or without IKKβ inhibitors and then stimulated with ATP or nigericin in the continued presence of LPS to activate the NLRP3 inflammasome. The results (Figs EV5) were similar to those obtained while studying the rapid activation of the NLRP3 inflammasome (Fig 3), except that the secretion of IL-1β into the culture medium could also be studied. The formation of p20, the activation of gasdermin D and the secretion of IL-1β and IL-18 were all suppressed by the inhibition of IKKβ (Fig EV5). These experiments could not be performed using IKKβ-deficient macrophages because the expression of pro-IL-1β is suppressed. Instead, we included a third structurally unrelated inhibitor of IKKβ in these studies (TPCA-1) added prior to stimulation with nigericin (Fig EV5).

As requested by the reviewer, we studied the activation of caspase-8 during the rapid activation of the NLRP3 inflammasome. We found that co-stimulation with LPS and nigericin activated the NLRP3 inflammasome without causing any activation of caspase-8. Incubation with the TAK1 inhibitor NG25, followed by co-stimulation with LPS and nigericin, did induce activation of caspase-8, but the activation of the NLRP3 inflammasome and gasdermin D was suppressed. The incubation of BMDM with any of three structurally unrelated inhibitors of IKKβ, followed by co-stimulation with LPS and nigericin, did not induce any activation of caspase-8 but suppressed the activation of the NLRP3 inflammasome and gasdermin D (**Appendix Fig 2**). Our results clearly exclude any involvement of caspase-8 in the rapid activation of the NLRP3 inflammasome or gasdermin D, which are IKKβ-dependent.

We now mention in the Discussion of the resubmitted paper the very slow spontaneous activation of the NLRP3 inflammasome observed from four hours onwards in TAK1 and IKKβ-deficient cells, or in the presence of TAK1 and IKKβ inhibitors. The mechanism is entirely different from the rapid activation of the NLRP3 inflammasome studied in our paper.

7. The authors were trying to investigate whether IKKβ facilitate TGN dispersion by activating small GTPase. Using bacterial enzyme CN04/CN03 to activate GTPase activity allows ATP or nigericin to activate inflammasome without LPS stimulation. Does it suggest that LPS leads GTPase activation through IKKβ? Does IKKβ inhibitor block CN04/CN03-induced caspase-1 cleavage? Does CN04/CN03 treatment lead to TGN dispersion or IKKβ activation?
We agree with the reviewer and with reviewers 2 and 3 that the observations made with CN03 and CN04, although interesting, are too preliminary and we have therefore removed them from the results section of the paper (Fig 6 of the original paper). It will be more appropriate to include more extensive experiments on CN03/CN04 in a future paper once we have identified the substrate(s) of IKKβ, whose phosphorylation is required for the formation and activation of the inflammasome.

Referee #2:

In this manuscript the authors investigate the role of IKKβ in controlling the formation and function of the NLRP3 inflammasome. Using a panel of pharmacological inhibitors they demonstrate that formation of the inflammasome does not require transcription or translation; however, the findings indicate that IKKβ plays a key role in this process. They further show that IKKβ regulates the dispersion of the TGN and they conclude that this role of IKKβ is separate from its function in the canonical NF-κB pathway. Finally, the data suggest that IKKβ might regulate caspase-1 activity by targeting proteins that lock Rho-GTPases into their active state. Overall the experiments are extremely well performed and the biochemical data are of the

highest quality. However, in its current form the findings are quite preliminary and the mechanism proposed is not fully supported by the data presented. There are a number of weaknesses outlined below that detract from the impact and significance of the study; however, the central finding that inhibiting IKKβ blocks the formation and function of the inflammasome is exciting and of wide interest in the field.

Major points:

1. The study relies very heavily on pharmacological inhibitors of IKKβ and other kinases. Further use of the genetic models available to the authors would add strong support to the overall conclusions. Moreover, the first two figures in the paper employ three separate inhibitors (one TAK1 inhibitor and two distinct IKKβ blockers) and this significantly strengthens the validity of these experiments. However, the remainder of the manuscript relies only on one compound (BI605906) thereby lessening the confidence that the effects are indeed specific for the TAK1-IKKβ signaling pathway.

As detailed at the start of our general response to all the reviewer's comments, we have carried out many additional experiments to address this issue, using immortalised macrophages from IKKβ-CXCR3-Cre (flox/flox) mice which express reduced levels of IKKβ. The experiments have also been repeated using more than one IKKβ inhibitor and with TPCA-1 an additional IKKβ inhibitor structurally unrelated to BI605906. All of these studies confirmed that IKKβ is required for the activation of gasdermin D and secretion of IL-18 (Fig 3), the oligomerisation of ASC (Fig 4), the association of NLRP3 with the TGN (Fig 5) and the formation of brighter and larger TGN positive puncta (Fig 6).

2. The data shown in Figure 3 do not add any mechanistic insight to the study. As noted above, the experiments are performed using only one inhibitor of IKKβ so any conclusions regarding the relative activity of the kinase in controlling NLRP3 v NF-κB activation are dependent on the results of only one pharmacological approach. Moreover, the authors use phosphorylation of NF-κB1 as the means to assess activation of the NF-κB pathway. The central mechanism controlling canonical NF-κB activation that is the primary function of IKKβ in the pathway, is phosphorylation of IkBa. Without extensive further pharmacokinetic analysis of IkB phosphorylation and NF-κB activation it is impossible to conclude from the experiments shown that there is a discernible difference in the activity of IKKβ in controlling NF-κB activation versus NLRP3.
We agree with the reviewer and have removed this figure from the revised manuscript, because it does not add any mechanistic insight to the study

3. The data shown in Figure 5 are interesting but do not definitively establish the link between IKKβ activity and activation of the inflammasome.
We have performed new Proximity Ligation Assays, which have established that NLRP3 co-localises with TGN38 during the rapid activation of the NLRP3 inflammasome induced by co-stimulation with LPS and nigericin, but not by stimulation with LPS or nigericin alone. The co-localisation of NLRP3 and TGN38 in primary BMDM is prevented by inhibitors of IKKβ and reduced in immortalised BMDM from mice expressing decreased levels of IKKβ (new figure 5). In the new Figure 6, studying the rapid activation of the NLRP3 inflammasome only, we show that IKKβ inhibitors have no effect on the dispersion of the TGN triggered by nigericin alone (Fig 6, panels G and H), but suppress the formation of the brighter puncta produced by co-stimulation with LPS plus nigericin (Fig. 6 panels B, C and D); i.e. the same conditions needed to observe co-localisation of NLRP3 with the dispersed TGN (Fig 5). The formation of these brighter and larger puncta is reduced in immortalised BMDM from IKKβ-CXCR3-Cre (flox/flox) mice.

4. The potential role of IKKβ in regulating Rho-GTPase function is implied but not established.
We agree with the reviewer and with reviewer 1 who made a similar comment. Although the observations made with CNO3 and CNO4 are interesting, we agree that they are too preliminary to include in this paper, and we have therefore removed them from the results section (Fig 6 of the original paper).

Minor:
1. The last section of the results describing the lack of effects on the AIM2 inflammasome would be better placed earlier in the manuscript.
We agree with the reviewer; this section is now placed earlier in the manuscript (Fig EV2C of the resubmitted paper).

2. The final paragraph of the results section is confusing and belongs in the discussion.
We have moved the last paragraph of Results to Discussion.

3. The labelling of Fig EV1A does not appear to be correct. Lanes 7 and 8 should be + for LPS.
We thank the reviewer for pointing out this mistake, which has been corrected in the revised paper

----
Referee #3:

This article by Nanda and colleagues extends on the recent work of James Chen's laboratory to report that IKKb is required for the dispersion of the trans-Golgi network, and resultant activation of the NLRP3 inflammasome. While the article raises several interesting points, there are several major and minor points that currently preclude publication in EMBO reports.

Major concerns:
1. Throughout the document, the authors analyse cell extracts for signs of inflammasome signaling (i.e. generation of p20 caspase-1) and presume that their absence means that inflammasome signaling was blocked. However, p20 is released from the cell extract into the cell culture media when cells lyse by pyroptosis. Thus, lack of p20 in cell extracts could be coupled to more p20 in cell supernatants, which would indicate augmented pyroptosis, rather than decreased inflammasome signaling. The authors should thus probe for caspase-1 in cell supernatants for all immunoblot experiments, to show that supernatants show a similar profile to cell extracts.
We thank the reviewer for making this important point. We have performed the experiments requested by immunoblotting for caspase-1 p20 and IL-18 in the cell culture medium. Our experiments show that the inhibition of IKKβ (using both pharmacological inhibitors or immortalised macrophages from mice expressing reduced levels of IKKβ) suppresses the appearance of p20, p10 and IL-18 in the cell culture medium, similar to the observations made in the cell extracts. This establishes that the reduced amounts of p20 and p10 in the extracts do not result from the augmented secretion of these components. We further show that gasdermin, which mediates pyroptosis, is produced during the rapid activation of the NLRP3 inflammasome and that this is also dependent on IKKβ. These data are now shown in the new Figure 3 and new Figure EV5.

2. Figure 5 is the key figure supporting the authors assertions. It shows that LPS+nigericin (but not control stimulations) trigger the redistribution of TGN38 into large puncta, which is blocked by the IKKbeta inhibitor BI605906. This figure would lend much stronger support to the

authors' assertions that IKKβ is required for TGN dispersal if it included a more specific method of IKKb antagonism (e.g. IKKb siRNA versus control siRNA, which the authors already use elsewhere). The authors also state that CN03 and CN04 can perform the same function as IKKb in triggering TGN dispersal; if this is the case, then adding CN03 and 04 to IKKb-silenced cells should restore dTGN puncta. Inclusion of these extra samples (with reagents and methods already at hand) would greatly strengthen the authors' hypotheses.

We have performed new Proximity Ligation Assays, which have established that NLRP3 co-localises with TGN38 during the rapid activation of the NLRP3 inflammasome induced by co-stimulation with LPS and nigericin, but not by stimulation with LPS or nigericin alone. The co-localisation of NLRP3 and TGN38 in primary BMDM is prevented by inhibitors of IKKβ and reduced in immortalised BMDM from mice expressing decreased levels of IKKβ (new figure 5). In the new Figure 6, studying the rapid activation of the NLRP3 inflammasome only, we show that IKKβ inhibitors have no effect on the dispersion of the TGN triggered by nigericin alone (Fig 6, panels G and H), but suppress the formation of the brighter puncta produced by co-stimulation with LPS plus nigericin (Fig. 6 panels B, C and D); i.e. the same conditions needed to observe co-localisation of NLRP3 with the dispersed TGN (Fig 5). The formation of these brighter puncta is reduced in immortalised BMDM from IKKβ-CXCR3-Cre (flox/flox) mice.

We agree with the Reviewer 3, and with Reviewer 1 and 2, that the experiments with CN03 and CN04 are too preliminary to include in the paper, and we have therefore removed them from the results (Fig 6 of the original paper). We agree that more extensive studies with CN03 and CN04 would be worthwhile and, depending on their outcome, plan to included them in a subsequent paper once the key substrate(s) of IKKβ, whose phosphorylation mediates the rapid activation of the NLRP3 inflammasome, has been identified.

3. The significance/interpretation of Figure EV5 is unclear. Are the authors suggesting that the "perinuclear vesicles" represent dTGN? Is it not possible that these are ASC specks? Without IF staining accompanying the bright field images, it is difficult to assess what these structures are, and how to interpret these data.

We agree with the reviewer that it is difficult to interpret the data in Fig EV5 of the original submitted version of the paper. Because this figure did not add any mechanistic insight, it has been deleted and replaced by the more definitive Proximity Ligation Assay, which has demonstrated an IKKβ-dependent colocalization of NLRP3 with TGN38 following co-stimulation with LPS and nigericin (new Fig 5).

4. Figure EV1 actD and cyx experiments should display NLRP3 blots so that the reader can see that NLRP3 levels are affected by these treatments.

We now present immunoblots showing that actinomycin and cycloheximide block the induction of NLRP3 observed after 4 h stimulation with LPS (Fig EV1C). We also show that actinomycin and cycloheximide block the LPS-stimulated induction of the immediate early gene DUSP1 observed after 30-60 min, which is inflammasome-independent (Fig EV1D). These experiments confirm that actinomycin and cycloheximide block transcription and translation, respectively, at the same concentrations used to demonstrate their lack of effect on the rapid activation of the NLRP3 inflammasome during this 30-60 minute experiment. Figures EV1E and EV1F also show that the expression of NLRP3 does not change during the rapid activation of the NLRP3 inflammasome in the presence or absence of actinomycin D and cycloheximide.

Please note that in Figs EV1C-EV1D we stimulated with LPS alone and did not co-stimulate with LPS and nigericin because we found that, unexpectedly, the presence of both nigericin and LPS strongly suppresses the induction of DUSP1 even in the absence of actinomycin and cycloheximide. This suggests that gene transcription may be inhibited when pyroptosis is triggered.

5. Throughout the document there are statements like (p3) "the NLRP3 inflammasome, whose activation requires both a PAMP and a DAMP". This is incorrect. Indeed, NLRP3 activation follows a 2 step mechanism, but there is no requirement for signal 1 and 2 to be fulfilled by a

PAMP followed by a DAMP. Both signal 1 and signal 2 can be PAMPs (e.g. LPS + nigericin, LPS+LLO). Signal 1 can be also be a cytokine (which can be a DAMP/alarmin such as IL-1a, or may not be, such as TNF, or IL-1b). So cytokine + DAMP (e.g. TNF+ATP), DAMP+DAMP (e.g. IL-1a + ATP) and cytokine + PAMP (e.g. TNF + nigericin) combinations of signal 1 and 2 are also possible. Please fix all of these sentences as they are confusing and incorrect. A related point (p10) "PAMP plus DAMP activation of the NLRP3 inflammasome that we have studied in this paper" refers to LPS+nigericin, which is PAMP+PAMP not PAMP+DAMP. Would be simpler to just say "LPS plus nigericin activation of the NLRP3 inflammasome that we have studied in this paper".
We thank the reviewer for pointing out the inaccurate wording, which has been rectified in the revised manuscript.

6. Some statements referring to "inflammasome priming" are clearly referring to transcriptional upregulation of NLRP3. Instead of using the term priming, the authors should instead just say "transcriptional upregulation of NLRP3", because it is well appreciated in the field that there are other mechanisms of non-transcriptional priming (e.g. NLRP3 post-translational modifications) that poise NLRP3 for signaling. For example (p4) "However, it is now clear that TLR signalling is also required for the rapid phase of caspase-1 activation, which does not require priming (Fernandes-Alnemri et al, 2013; Lin et al, 2014)." Incidentally, I believe that PMID22898390 was the first article to show rapid TLR-mediated effects on NLRP3 activity (before the cited references were published).
We now use the term "transcriptional upregulation of NLRP3" instead of "inflammasome priming". We have corrected the referencing in the Introduction to cite the original paper which demonstrated the rapid TLR-mediated effects on NLRP3 expression.

7. Several western blot figures demonstrate cleavage of inflammasome-related proteins (e.g. caspase-1). These should be annotated with molecular weight markers so that the reader can assess for themselves that the cleavage fragments are indeed the correct size.
We now include the positions of molecular mass markers in all the immunoblots presented in the paper.

8. Please clarify the figure captions: (1) If signal 1 and 2 are co-administrated (and not sequentially administered) throughout the article's experiments, please change "administered" to "co-administered". (2) For some of captions it is unclear how many independent biological replicate data sets were performed.
We agree with the reviewer and now use the term "co-stimulated" instead of "administered" in the text and figure legends of the paper. The number of independent biological data sets performed for each experiment is now mentioned in each figure legend.

Minor concerns:

1. Throughout the document nigericin is referred to as a pore-forming toxin, which is not strictly true (it is a potassium ionophore).
This error has been rectified.

2. Abstract line 4: "allowing it to cleave IL-1" should be "allowing it to cleave IL-1beta".
IL-1 has been changed to IL-1β as requested.

3. Page 3 "The CARD domain can then recruit caspase-1 through CARD/CARD interactions, leading to the "autocleavage" of caspase-1", should be "The CARD domain can then recruit caspase-1 through CARD/CARD interactions, leading to the dimerization and autocleavage of caspase-1". Dimerization is important because this confers protease activity to caspase-1, such is needed for autocleavage. I do not see why autocleavage should be in inverted commas.
We have used the wording suggested by the reviewer in the revised, re-submitted version of the paper.

4. Page 7 referring to full length caspase-1 in triton insoluble fraction: "as well as aggregates of uncleaved caspase-1; this did not occur in cells treated with BI605906 (Fig 4A)". Inclusion of full length caspase-1 in the insoluble fraction does not necessarily mean that caspase-1 is aggregated (for example, it could be attached to the cytosolic face of an organelle, or the purity of the fraction may be questionable). In fact, it is likely that this fraction has captured monomeric caspase-1, as caspase-1 p45 is present at similar levels throughout all stimulations (including inflammasome negative controls).
We agree with the reviewer and have reworded the text appropriately. We have modified the figure and focussed on formation of ASC oligomer in this figure. We have also added an additional experiment using immortalised BMDM from IKKβ-deficient mice, which provides genetic evidence that the translocation of ASC to the Triton-X-100-insoluble fraction is IKKβ-dependent.

5.Figure EV1a labels suggest that caspase-1 p20 is generated in unprimed cells treated with ATP and ActD. I presume this is a mistake? Should the LPS row be marked + for these samples?
We thank the reviewer for noticing this error, which has been corrected in the revised paper.

6. Figure EV1 data would be more compelling if LPS-induced (inflammasome-unrelated) control proteins were shown to be blocked by ActD/Cyx.
Please see our response to Major point 4.

1st Revision - Editorial Decision 7th Jul 2021

Dear Dr. Nanda,

Thank you for the submission of your revised manuscript to our editorial offices. We have now received the reports from the three referees that were asked to re-evaluate your study, you will find below. As you will see, referees #1 and #2 are satisfied by the revision, whereas referees #3 has some further concerns and suggestions to improve the manuscript, we ask you to address in a final revised version (either experimentally, or with good arguments). Please also provide a detailed point-by-point response regarding the remaining referee concerns.

Please note that as the study indicated by referee #3 to take away novelty was published while the present manuscript was under revision, the EMBO press scooping protection applies, i.e. your work is protected, and the other work is not considered relevant to the editorial assessment of the conceptual advance/novelty of your revised manuscript.

Moreover, I have these editorial requests I also ask you to address:

- Could you add a few introductory words to the abstract, or partly re-write it that it does not start with 'We report that ... '? But please take care that the final abstract has not more than 175 words.

- Please make sure that each figure panel is called out and that the panels are called out sequentially. Presently Fig. 6E and 6F are called out before 6B, and Fig EV4A and B are called out after EV4E. Please fix this or change the order of the panels in the respective figures.

- I seems that the two Appendix figures could also be added to the EV figures. Some of them (e.g. EV4) are not very crowded. I would suggest doing that, and then update the callouts and the legends in the manuscript text. We then would not need and Appendix file.

- If you prefer to show the two figures in an Appendix, please add a table of contents to the Appendix, including page numbers. Please also move the legends for each figure below the specific figure. I think that is easier to comprehend. Please also correct the names and callouts for the Appendix figure to 'Appendix Figure Sx'.

- Please add scale bars of similar style and thickness to all the images, using clearly visible black or white bars (depending on the background). Please place these in the lower right corner of the images. Please do not write on or near the bars in the image but define the size only in the respective figure legend.

- Please use abbreviations of the author names in the author contributions.

- Please format the references according to journal style (journal names need to be italicized). See: http://www.embopress.org/page/journal/14693178/authorguide#referencesformat

- As part of the Western blots shown are significantly cropped, please provide the source data for all the blots (main, EV and Appendix figures). The source data will be published in a separate source data file online along with the accepted manuscript and will be linked to the relevant figure. Please submit the source data (scans of entire blots) together with the revised manuscript. Please include size markers for scans of entire blots, label the scans with figure and panel number and send one PDF file per figure (main and EV figures). Please send one PDF file for Appendix figures.

- Please note that all corresponding authors are required to supply an ORCID ID for their name upon submission of a revised manuscript. We will not proceed with publication if this is not done. The ORCID of Philip Cohen is still missing. Please find instructions on how to link the ORCID ID to the account in our manuscript tracking system in our Author guidelines:
http://www.embopress.org/page/journal/14693178/authorguide#authorshipguidelines

- Finally, please find attached a word file of the manuscript text (provided by our publisher) with changes we ask you to include in your final manuscript text, and some queries, we ask you to address. Please provide your final manuscript file with track changes, in order that we can see any modifications done.

In addition, I would need from you:
- a short, two-sentence summary of the manuscript (35 words).
- three to four bullet points highlighting the key findings of your study.
- a schematic summary figure (synopsis image) in jpeg or tiff format with the exact width of 550 pixels and a height of not more than 400 pixels that can be used as a visual synopsis on our website.

I look forward to seeing the final revised version of your manuscript when it is ready. Please let me know if you have questions regarding the revision.

Kind regards,

Achim Breiling
Editor
EMBO Reports

Referee #1:

This authors have addressed most of my concerns very well. The Data quality looks decent and clear. No more revisions are suggested.

Referee #2:

The authors have expertly addressed all of the critique points from all three referees.

My main concerns were that the origin study was quite preliminary and that the mechanism proposed was not fully supported by the data presented. The authors have greatly strengthened the conclusions by the inclusion of extensive new data and the removal of figures that did not add to the overall significance. In particular, the new experiments using the IKKb-CXCR3-Cre (flox/flox) mice, and the added experiments using additional IKKb inhibitors support the conclusions. The new proximity ligation assay is also very strong and establishes that the colocalization of NLRP3 and TGN38 is inhibited when IKKb activity is suppressed. Taken together with other substantial revisions, these new approaches and figures substantially improve the impact and significance of this manuscript

----
Referee #3:

The manuscript from Nanda et al was improved by study revision, particularly through the greater use of knockdown/knockout techniques to support inhibitors findings, and the inclusion of a third (structurally distinct) inhibitor, TPCA-1.

Manuscript revision appears (understandably) to have been delayed as a consequence of the COVID19 pandemic. Unfortunately, during revision, another study was published (Feb 2021) by Novartis that overlaps substantially with the current study's findings both in their finding that IKKb facilitates NLRP3 inflammasome signalling, and their use of the inhibitor TPCA-1 (https://doi.org/10.1016/j.bbrc.2021.01.051, 'Pharmacological inhibition of IKKβ dampens NLRP3 inflammasome activation after priming in the human myeloid cell line THP-1'). This publication (not cited by the authors) reports that IKKb is partially required for NLRP3-induced ASC speck formation, and is required for full caspase-1 activation by the NLRP3 inflammasome. While there are some slight differences in the findings and approaches of the two studies, the published study substantially decreases the novelty of the submitted manuscript. Essentially all of the early figures (Figs 1-4) in the revised manuscript report the same findings as the Novartis report, and one figure panel (Fig 2A) overlaps substantially with findings from the Martin et al study cited in the references. Thus the novelty of the first 4 figures is rather limited.

The final two main figures (Fig 5-6) aim to provide some understanding of the mechanism(s) by which IKK supports NLRP3 inflammasome signalling. The authors' interpretation of these data is that IKKb is required for dispersal of the TGN (Fig 6) and recruitment of NLRP3 to the dTGN (Fig 5). On the face of it, these figures bring interesting mechanistic detail to the function of IKKb in NLRP3 signalling. However there are some issues. The authors measure TGN dispersal and NLRP3 recruitment to the dTGN, using the TGN marker TGN38. Fig 6 and EV4 shows that indeed (as previously published by Chen & Chen), TGN38 redistributes to bright small puncta upon cell treatment with LPS+Nigericin. Strangely, however, application of IKKb inhibitors (or IKKb knockout) does not revert the TGN38 staining back to a phenotype similar to the untreated control cells; instead TGN38 staining is essentially lost from the cell. So while these inhibitors 'block' the formation of the dTGN, this appears to be a result of the cell losing all TGN staining (dispersed or otherwise). From this, it is extremely unclear exactly what IKKb does in this setting - it seems to support the TGN (or TGN38 specifically) rather than dispersal. Following on from this, Fig 5 uses PLA to detect interactions between NLRP3 and TGN38, and reports that IKKb inhibition or deficiency blocks this interaction. However, as TGN38 staining is essentially lost from IKKb inhibited/deficient cells, it is unsurprising that PLA does not detect TGN38-NLRP3 interactions in this setting.

For the reasons above, it is the view of this Reviewer that the current study has limited novelty, and lacks clarity about whether and how IKKbeta might contribute to TGN dispersal. Recommendation for EMBO Reports: reject.

Minor typographical issues:
1. The cleaved form of GSDMD is usually referred to as GSDMD p30 or GSDMD-NT, rather than CL
2. Page 5 "(i)" in the sentence calling out Fig 3C seems unnecessary.
3. Figure 6 call outs are not in order.

EMBOR-2020-50743V2

Dear Achim,
Thank you for your e.mail below inviting submission of a final version of our paper, which we are now submitting on line. The final version complies with all of the Editorial requests made in your e.mail. It also contains a Synopsis, comprising a 35 word summary of the paper, three bullet points highlighting the key findings of the study and a figure for the synopsis. We have incorporated the two Appendix figures into Figure EV4, as you suggested.

Point by point response to the comments made by reviewer 3 about the revised version of paper.

Reviewer 3's comments "The final two main figures (Fig 5-6) aim to provide some understanding of the mechanism(s) by which IKK supports NLRP3 inflammasome signalling. The authors' interpretation of these data is that IKKβ is required for dispersal of the TGN (Fig 6) and recruitment of NLRP3 to the dTGN (Fig 5). On the face of it, these figures bring interesting mechanistic detail to the function of IKKβ in NLRP3 signalling. However there are some issues. The authors measure TGN dispersal and NLRP3 recruitment to the dTGN, using the TGN marker TGN38. Fig 6 and EV4 shows that indeed (as previously published by Chen & Chen), TGN38 redistributes to bright small puncta upon cell treatment with LPS+Nigericin. Strangely, however, application of IKKβ inhibitors (or IKKβ knockout) does not revert the TGN38 staining back to a phenotype similar to the untreated control cells; instead TGN38 staining is essentially lost from the cell. So while these inhibitors 'block' the formation of the dTGN, this appears to be a result of the cell losing all TGN staining (dispersed or otherwise). From this, it is extremely unclear exactly what IKKβ does in this setting - it seems to support the TGN (or TGN38 specifically) rather than dispersal. Following on from this, Fig 5 uses PLA to detect interactions between NLRP3 and TGN38, and reports that IKKβ inhibition or deficiency blocks this interaction. However, as TGN38 staining is essentially lost from IKKβ inhibited/deficient cells, it is unsurprising that PLA does not detect TGN38-NLRP3 interactions in this setting. For the reasons above, it is the view of this Reviewer that the current study lacks clarity about whether and how IKKbeta might contribute to TGN dispersal."

Our response. We realize from the comments made by Reviewer 3 that we did not present and explain the results in figures 5 and 6 clearly enough, which has led the reviewer to misunderstand some of the results presented.

Figure 5 is a proximity ligation assay. It does not measure TGN38 staining but whether NLRP3 and TGN38 come into close proximity with one another. The experiment employs two different antibodies, one recognising NLRP3 and the other TGN38, and a positive PLA signal is seen only when both NLRP3 and TGN38 come into close proximity. Figure 5 shows that stimulation of primary macrophages with both LPS and nigericin is required for NLRP3 and TGN38 to come into proximity and that this is prevented by suppression of IKKβ activity. We think that the results presented are clear and establish that IKKβ activity is required for interaction between NLRP3 and TGN38, strengthening the evidence presented in Figure 4 that IKKβ activity is critical for NLRP3 assembly and making a novel mechanistic point. We have rewritten the text that describes these results more carefully.

Figure 6. We have prepared a new Figure 6 using different fields from the same experiment, that we think illustrate the points we are making more clearly. We have also rearranged the "Call-Outs" in chronological order, we think think has also aided clarity. The key findings presented in this figure are that (a) nigericin alone stimulates dispersion of the TGN by an IKKβ-independent mechanism, forming multiple small TGN38-positive puncta, which mostly retain a perinuclear location (panels A-D); stimulation with LPS alone does not cause any dispersal of the TGN (panel E); (c) co-stimulation with LPS and nigericin increases the extent of TGN dispersal (panel F) compared to that observed after stimulation with nigericin alone, (panel B) with many TGN-positive puncta no longer retaining a perinuclear location (panel F). Incubation with IKKβ inhibitors prevents the effect seen after co-stimulation with LPS and nigericin, but not the effect of nigericin alone (panels G and H). For this reason IKKβ inhibitors do not restablish the TGN as in the control cells (panel A), but only change the extent of dispersion of the puncta to that seen with nigerin alone (compare panels G and H with panel B). We have rewritten the relevant section of the text to improve clarity. Taken together, the results establish that LPS (which activates IKKβ) has an additional effect on TGN38-positive puncta formation to that mediated by nigericin alone. With respect, we did not conclude in the revised version of the paper that "IKKβ is required for dispersal of the TGN" and this was not our interpretation of the results.

Disappearance of TGN38 staining.

With respect, stimulation with LPS and nigericin has not caused TGN38 to "disappear". The TGN38 staining only appears weaker than in unstimulated cells, because TGN38 is no longer localised to intact golgi, but instead dispersed throughout the cell into a huge number of small puncta. This effect is exaggerated by confocal microscopy which takes only a two-dimensional image of a thin section of a cell.

Transcription independent vs transcription-dependent activation of the inflammasome.

To our knowledge, the results presented in Fig 6 are first to study puncta formation during the rapid, transcription-independent activation of the NLRP3 inflammasome. Previous studies in primary macrophages were performed after prolonged stimulation with LPS to increase NLRP3 expression, followed by stimulation with nigericin. This induces the formation of much larger, brighter punta than those observed during the rapid transcription-independent activation of the NLRP3 inflammasome, as shown in the new Fig EV4E, but the independent effects of LPS and nigericin cannot be studied independently. Studying puncta formation during the rapid activation of the inflammasome is more challenging due to the low levels of NLRP3 expression, but an advantage is that the additional effect of co-stimulation with LPS and nigericin, compared to stimulation with nigericin alone, could be detected for the first time. We sincerely thank the reviewer for the comments made, which have enabled us to improve the results and explain them more clearly.

Reviewer 2's cross commenting on Reviewer 3's report. 'The 3rd reviewer's point regarding Fig 6 is valid as the data presented are quite difficult to interpret. The distinction between puncta and dispersal of the TGN is not clear although differences are evident. Quantitative analysis of staining intensity / puncta formation might help strengthen these experiments and the authors should provide an explanation for the failure to re-establish the TGN in the presence of IKKβ inhibition**.**

Our response.

We have prepared a new Figure 6 using different fields from the same experiment and rearranged the panels in the order in which they are described in the text.. We have also rewritten the relevant section of the text to spell out the findings more carefully A key finding is that nigericin alone stimulates dispersion of the TGN by an IKKβ-independent mechanism, forming multiple small TGN38-positive puncta, which mostly retained in a perinuclear location (panels A-D). We would therefore **not** expect IKKβ inhibitors to restablish the TGN as in the control cells (panel A), but only to change the extent of dispersion of the puncta to that seen with nigerin alone, which is exactly what we do see (compare panels G and H with panel B). Taken together, we think that the results now show convincingly that LPS (which activates IKKβ) has an additional effect on the extent of dispersal of the TGN38-positive puncta to that mediated by nigericin alone.

At the reviewer's suggestion, we tried to come up with a way of quantitating the data in Fig 6, but after exploring a few ideas, decided that it was not possible. However, we believe that the improvements we have now made to Figure 6 are sufficient to clarify the major observations made.

Yours sincerely

Sambit Nanda and Philip Cohen

2nd Revision - Editorial Decision 30th Jul 2021

Dr. Sambit Nanda
University of Dundee
MRC Protein Phosphorylation and Ubiquitylation Unit
Sir James Black Center, Dow Street
School of Life Sciences
Dundee DD1 5EH
United Kingdom

Dear Dr. Nanda,

I am very pleased to accept your manuscript for publication in the next available issue of EMBO reports. Thank you for your contribution to our journal.

At the end of this email I include important information about how to proceed. Please ensure that you take the time to read the information and complete and return the necessary forms to allow us to publish your manuscript as quickly as possible.

As part of the EMBO publication's Transparent Editorial Process, EMBO reports publishes online a Review Process File to accompany accepted manuscripts. As you are aware, this File will be published in conjunction with your paper and will include the referee reports, your point-by-point response and all pertinent correspondence relating to the manuscript.

If you do NOT want this File to be published, please inform the editorial office within 2 days, if you have not done so already, otherwise the File will be published by default [contact: emboreports@embo.org]. If you do opt out, the Review Process File link will point to the following statement: "No Review Process File is available with this article, as the authors have chosen not to make the review process public in this case."

Should you be planning a Press Release on your article, please get in contact with emboreports@wiley.com as early as possible, in order to coordinate publication and release dates.

Thank you again for your contribution to EMBO reports and congratulations on a successful publication. Please consider us again in the future for your most exciting work.

Yours sincerely,

Achim Breiling
Editor
EMBO Reports

****

THINGS TO DO NOW:

You will receive proofs by e-mail approximately 2-3 weeks after all relevant files have been sent to our Production Office; you should return your corrections within 2 days of receiving the proofs.

Please inform us if there is likely to be any difficulty in reaching you at the above address at that time. Failure to meet our deadlines may result in a delay of publication, or publication without your corrections.

All further communications concerning your paper should quote reference number EMBOR-2020-50743V3 and be addressed to emboreports@wiley.com.

Should you be planning a Press Release on your article, please get in contact with emboreports@wiley.com as early as possible, in order to coordinate publication and release dates.

EMBO PRESS

YOU MUST COMPLETE ALL CELLS WITH A PINK BACKGROUND ➔

PLEASE NOTE THAT THIS CHECKLIST WILL BE PUBLISHED ALONGSIDE YOUR PAPER

Corresponding Author Name: Sambit Kumar Nanda

Journal Submitted to: EMBO Reports

Manuscript Number: EMBOR-2020-50743V1

Reporting Checklist For Life Sciences Articles (Rev. June 2017)

This checklist is used to ensure good reporting standards and to improve the reproducibility of published results. These guidelines are consistent with the Principles and Guidelines for Reporting Preclinical Research issued by the NIH in 2014. Please follow the journal's authorship guidelines in preparing your manuscript.

A- Figures
1. Data

The data shown in figures should satisfy the following conditions:

- the data were obtained and processed according to the field's best practice and are presented to reflect the results of the experiments in an accurate and unbiased manner.
- figure panels include only data points, measurements or observations that can be compared to each other in a scientifically meaningful way.
- graphs include clearly labeled error bars for independent experiments and sample sizes. Unless justified, error bars should not be shown for technical replicates.
- if n< 5, the individual data points from each experiment should be plotted and any statistical test employed should be justified
- Source Data should be included to report the data underlying graphs. Please follow the guidelines set out in the author ship guidelines on Data Presentation.

2. Captions

Each figure caption should contain the following information, for each panel where they are relevant:

- a specification of the experimental system investigated (eg cell line, species name).
- the assay(s) and method(s) used to carry out the reported observations and measurements
- an explicit mention of the biological and chemical entity(ies) that are being measured.
- an explicit mention of the biological and chemical entity(ies) that are altered/varied/perturbed in a controlled manner.

- the exact sample size (n) for each experimental group/condition, given as a number, not a range;
- a description of the sample collection allowing the reader to understand whether the samples represent technical or biological replicates (including how many animals, litters, cultures, etc.).
- a statement of how many times the experiment shown was independently replicated in the laboratory.
- definitions of statistical methods and measures:
  - common tests, such as t-test (please specify whether paired vs. unpaired), simple χ2 tests, Wilcoxon and Mann-Whitney tests, can be unambiguously identified by name only, but more complex techniques should be described in the methods section;
  - are tests one-sided or two-sided?
  - are there adjustments for multiple comparisons?
  - exact statistical test results, e.g., P values = x but not P values < x;
  - definition of 'center values' as median or average;
  - definition of error bars as s.d. or s.e.m.

Any descriptions too long for the figure legend should be included in the methods section and/or with the source data.

In the pink boxes below, please ensure that the answers to the following questions are reported in the manuscript itself. Every question should be answered. If the question is not relevant to your research, please write NA (non applicable). We encourage you to include a specific subsection in the methods section for statistics, reagents, animal models and human subjects.

B- Statistics and general methods

Please fill out these boxes ➔ (Do not worry if you cannot see all your text once you press return)

1.a. How was the sample size chosen to ensure adequate power to detect a pre-specified effect size?	For PLA quantitation: The number of fields choosen for quantitation (10 fields per sample) was based on previous studies.
1.b. For animal studies, include a statement about sample size estimate even if no statistical methods were used.	This paper does not contain any animal studies, although cells isolated from animals were used in the study.
2. Describe inclusion/exclusion criteria if samples or animals were excluded from the analysis. Were the criteria pre-established?	No sample were excluded from the analysis
3. Were any steps taken to minimize the effects of subjective bias when allocating animals/samples to treatment (e.g. randomization procedure)? If yes, please describe.	The fields choosen for microscopy imaging (TGN38 dispersion and Proximity Ligation Assay (PLA)) were randomly selected. The fields of view and plane of focus were chosen using the DAPI channel without viewing the TGN38 or the PLA signal. A statement detailing this is included in the Materials and Methods section (page 15-16).
For animal studies, include a statement about randomization even if no randomization was used.	Not applicable
4.a. Were any steps taken to minimize the effects of subjective bias during group allocation or/and when assessing results (e.g. blinding of the investigator)? If yes please describe.	Not applicable
4.b. For animal studies, include a statement about blinding even if no blinding was done	Not applicable
5. For every figure, are statistical tests justified as appropriate?	Yes (Please see page 16, 24 and 27)
Do the data meet the assumptions of the tests (e.g., normal distribution)? Describe any methods used to assess it.	We have used Shapiro-Wilk test to determine if the data have normal distribution. Where the distribution of data wasn't normal, non-parametric test such as Kruskal Wallis test with Dunn's multiple comparison was used to calculate significance between groups (Figure 5M). For figure 5N, where the data has normal distribution, two-way ANOVA with Sidak's multiple comparison test has been used.
Is there an estimate of variation within each group of data?	We included std. deviation and std error of mean in the source data provided.

USEFUL LINKS FOR COMPLETING THIS FORM

http://www.antibodypedia.com
http://1degreebio.org
http://www.equator-network.org/reporting-guidelines/improving-bioscience-research-repor
http://grants.nih.gov/grants/olaw/olaw.htm
http://www.mrc.ac.uk/Ourresearch/Ethicsresearchguidance/Useofanimals/index.htm
http://ClinicalTrials.gov
http://www.consort-statement.org
http://www.consort-statement.org/checklists/view/32-consort/66-title
http://www.equator-network.org/reporting-guidelines/reporting-recommendations-for-tumo
http://datadryad.org
http://figshare.com
http://www.ncbi.nlm.nih.gov/gap
http://www.ebi.ac.uk/ega
http://biomodels.net/
http://biomodels.net/miriam/
http://jjj.biochem.sun.ac.za
http://oba.od.nih.gov/biosecurity/biosecurity_documents.html
http://www.selectagents.gov/

Is the variance similar between the groups that are being statistically compared?	We have included co-efficient of variance in the source data provided.

C- Reagents

6. To show that antibodies were profiled for use in the system under study (assay and species), provide a citation, catalog number and/or clone number, supplementary information or reference to an antibody validation profile. e.g., Antibodypedia (see link list at top right), 1DegreeBio (see link list at top right).	Catalog number or clone number of each antibody used have been provided in the Method section.
7. Identify the source of cell lines and report if they were recently authenticated (e.g., by STR profiling) and tested for mycoplasma contamination.	All cell lines used in ths study were mycoplasma negative. A statement stating this has been included in the Materials and Methods section (page 13)

* for all hyperlinks, please see the table at the top right of the document

D- Animal Models

8. Report species, strain, gender, age of animals and genetic modification status where applicable. Please detail housing and husbandry conditions and the source of animals.	We have reported strain of mice used including genetic modifications. Details housing and husbandry conditions of wild type animals used as the source of bone marrow cells in this study has been described in the Materials and Method section. Please see page 12 and 13).
9. For experiments involving live vertebrates, include a statement of compliance with ethical regulations and identify the committee(s) approving the experiments.	A statement of compliance with ethical regulations and committee approving breeding and maintenance of wild type animals has been described in the Method section (page 12-13)
10. We recommend consulting the ARRIVE guidelines (see link list at top right) (PLoS Biol. 8(6), e1000412, 2010) to ensure that other relevant aspects of animal studies are adequately reported. See author guidelines, under 'Reporting Guidelines'. See also: NIH (see link list at top right) and MRC (see link list at top right) recommendations. Please confirm compliance.	Maintence and breeding of wild type animals used in this study have been carried out as per the ARRIVE guidelines (page 12-13)

E- Human Subjects

11. Identify the committee(s) approving the study protocol.	Not applicable.
12. Include a statement confirming that informed consent was obtained from all subjects and that the experiments conformed to the principles set out in the WMA Declaration of Helsinki and the Department of Health and Human Services Belmont Report.	Not applicable.
13. For publication of patient photos, include a statement confirming that consent to publish was obtained.	Not applicable.
14. Report any restrictions on the availability (and/or on the use) of human data or samples.	Not applicable.
15. Report the clinical trial registration number (at ClinicalTrials.gov or equivalent), where applicable.	Not applicable.
16. For phase II and III randomized controlled trials, please refer to the CONSORT flow diagram (see link list at top right) and submit the CONSORT checklist (see link list at top right) with your submission. See author guidelines, under 'Reporting Guidelines'. Please confirm you have submitted this list.	Not applicable.
17. For tumor marker prognostic studies, we recommend that you follow the REMARK reporting guidelines (see link list at top right). See author guidelines, under 'Reporting Guidelines'. Please confirm you have followed these guidelines.	Not applicable.

F- Data Accessibility

18: Provide a "Data Availability" section at the end of the Materials & Methods, listing the accession codes for data generated in this study and deposited in a public database (e.g. RNA-Seq data: Gene Expression Omnibus GSE39462, Proteomics data: PRIDE PXD000208 etc.) Please refer to our author guidelines for 'Data Deposition'.<br><br>Data deposition in a public repository is mandatory for:<br>a. Protein, DNA and RNA sequences<br>b. Macromolecular structures<br>c. Crystallographic data for small molecules<br>d. Functional genomics data<br>e. Proteomics and molecular interactions	Not applicable. Therefore we have added a sentence at the end of Materials and Method section: No primary data set have been generated and deposited (page 16).
19. Deposition is strongly recommended for any datasets that are central and integral to the study; please consider the journal's data policy. If no structured public repository exists for a given data type, we encourage the provision of datasets in the manuscript as a Supplementary Document (see author guidelines under 'Expanded View' or in unstructured repositories such as Dryad (see link list at top right) or Figshare (see link list at top right).	Not applicable.
20. Access to human clinical and genomic datasets should be provided with as few restrictions as possible while respecting ethical obligations to the patients and relevant medical and legal issues. If practically possible and compatible with the individual consent agreement used in the study, such data should be deposited in one of the major public access-controlled repositories such as dbGAP (see link list at top right) or EGA (see link list at top right).	Not Applicable
21. Computational models that are central and integral to a study should be shared without restrictions and provided in a machine-readable form. The relevant accession numbers or links should be provided. When possible, standardized format (SBML, CellML) should be used instead of scripts (e.g. MATLAB). Authors are strongly encouraged to follow the MIRIAM guidelines (see link list at top right) and deposit their model in a public database such as Biomodels (see link list at top right) or JWS Online (see link list at top right). If computer source code is provided with the paper, it should be deposited in a public repository or included in supplementary information.	Not Applicable.

G- Dual use research of concern

22. Could your study fall under dual use research restrictions? Please check biosecurity documents (see link list at top right) and list of select agents and toxins (APHIS/CDC) (see link list at top right). According to our biosecurity guidelines, provide a statement only if it could.	Not applicable
